# Performance of a multi-cancer early detection test in the randomized controlled NHS-Galleri trial

**DOI:** 10.1038/s41591-026-04652-8

**Published:** 2026-09-22

**Authors:** Richard D. Neal, Saoirse Dolly, Peter Johnson, Helen Jones, Sir Harpal Kumar, Lennard Y. W. Lee, Yujin Lee, Wei Liang, Thomas Round, Rebecca Smittenaar, Jane Warwick, Peter Sasieni, Charles Swanton

**Affiliations:** 1 University of Exeter, Exeter, UK; 2 Guy’s and St. Thomas’ NHS Foundation Trust, London, UK; 3 NHS England, London, UK; 4 EMS Healthcare, Cheshire, UK; 5 GRAIL Bio UK Ltd., London, UK; 6 University of Oxford, Oxford, UK; 7 Queen Mary University of London, London, UK; 8 King’s College London, London, UK; 9 The Francis Crick Institute, London, UK; 10 University College London, London, UK

**Keywords:** Cancer screening, Biotechnology

## Abstract

The NHS-Galleri trial was a randomized controlled trial evaluating a multi-cancer early detection (MCED) test added to usual care. We reported elsewhere that the primary endpoint of a reduction in the incidence of stage III/IV cancer diagnoses in the intervention arm versus control arm was not met. Here we report prespecified secondary test performance endpoints among evaluable intervention arm participants across three annual screening rounds. These analyses were descriptive; there was no hypothesis testing. Participants aged 50−77 years (*N* = 142,250) were randomized 1:1 into intervention (MCED) or control arms. Intervention arm participants with positive MCED results were referred to National Health Service (NHS) standard-of-care pathways for diagnostic workup, with referrals informed by the predicted cancer signal origin (CSO). Positive test results were returned for 722 of 70,325 (1.03%), 518 of 64,498 (0.80%) and 561 of 62,323 (0.90%) participants in rounds 1−3, respectively. In aggregate, 937 participants had MCED-detected primary cancers. Respective by-round cancer detection rates were 0.60%, 0.40% and 0.41%; positive predictive values were 58.0% (419/722), 50.4% (261/518) and 45.8% (257/561); and negative predictive values were 98.98% (68,895/69,603), 98.90% (63,278/63,980) and 98.86% (61,058/61,762). Across rounds, specificity ranged from 99.50% to 99.60%; episode sensitivity ranged from 26.7% to 37.2% for all cancers and from 47.6% to 63.4% for 12 prespecified types; and CSO accuracy ranged from 91.1% to 93.6%. These data provide insights on the performance of the MCED test in population screening within the NHS setting. ClinicalTrials.gov: NCT05611632; ISRCTN: ISRCTN91431511.

## Abstract (web summary)

Secondary endpoint analyses in the NHS-Galleri randomized controlled trial, testing a multi-cancer early detection test added to usual care, provide insights on the performance of the blood-based test in more than 142,000 participants without suspicion of cancer.

## Main

Cancer screening is associated with improved outcomes, driven partly by earlier-stage diagnosis that can result in a meaningful extension of survival^1–4^. Earlier detection of cancer may also increase opportunities for treatment with curative intent for certain cancer types, even in those with stage III disease^5–8^. However, screening is currently recommended only for limited cancer types (for example, breast, cervical, colorectal and lung (high-risk individuals))^9,10^, leaving most cancers unscreened. As a result, only approximately 6% of cancers in England (in 2020) and approximately 14% of cancers in the United States (in 2017) are detected through guideline-recommended population screening^4,11^.

Population-wide implementation of many additional single-cancer screening programs to detect the majority of cancer types for which screening is not currently recommended is not feasible given the low prevalence of individual cancer types, cumulative burden of false positives, high cost, logistical complications and the lack of candidate technologies. Blood-based MCED tests, however, can screen for multiple cancer types concurrently by detecting a shared signal while maintaining a low false-positive rate^12–16^.

One MCED test, Galleri (GRAIL Inc.), detects tumor methylation patterns in cell-free DNA isolated from peripheral blood and returns a positive (cancer signal detected) result with predicted CSO(s) to guide diagnostic evaluations^14,17^. Previous large-scale case−control studies^14,17,18^, prospective studies^15,16,19^ and real-world analyses^20–24^ have demonstrated the test’s performance (positive predictive value (PPV) 43.1−78.6%, specificity 99.3−99.6%, top-1 CSO prediction accuracy 85.7−93.3%)^14–17,19,21,22,24^, safety in intended-use populations^15,16,19^ and feasibility in clinical practice in the United States^20–24^.

NHS-Galleri (NCT05611632, registration date 28 October 2022; ISRCTN91431511, registration date 16 September 2021) is a randomized controlled trial evaluating the clinical utility and performance of an MCED test added to usual care^25^ within the NHS in England. Participants were assigned to an intervention arm, in which blood samples were tested using the MCED test, or to a usual care control arm, in which samples were collected and stored, across three annual screening rounds. The primary endpoints, selected secondary endpoints and safety data are reported separately^26^. Here we report MCED test performance—a prespecified secondary endpoint—and related exploratory endpoints in the intervention arm across three rounds of annual screening.

## Results

### Trial endpoints

The primary endpoint of the NHS-Galleri trial—reduction in the incidence rate of stage III/IV cancers in the intervention arm versus the control arm—was not met and is reported elsewhere^26^. Key secondary endpoints reported in that publication include the reduction in the incidence rate of stage IV cancers in the intervention arm versus the control arm and an evaluation of safety, including adverse events. Here we report the secondary endpoint of MCED test performance in the intervention arm and related exploratory endpoints. Other secondary endpoints, including assessing the impact of MCED test usage on healthcare resource utilization and on participant-reported psychological outcomes, comparing cancer-specific mortality between the intervention and control arms and assessing the potential impact of overdiagnosis, will be reported in future publications.

### Participant characteristics

**Fig. 1:**
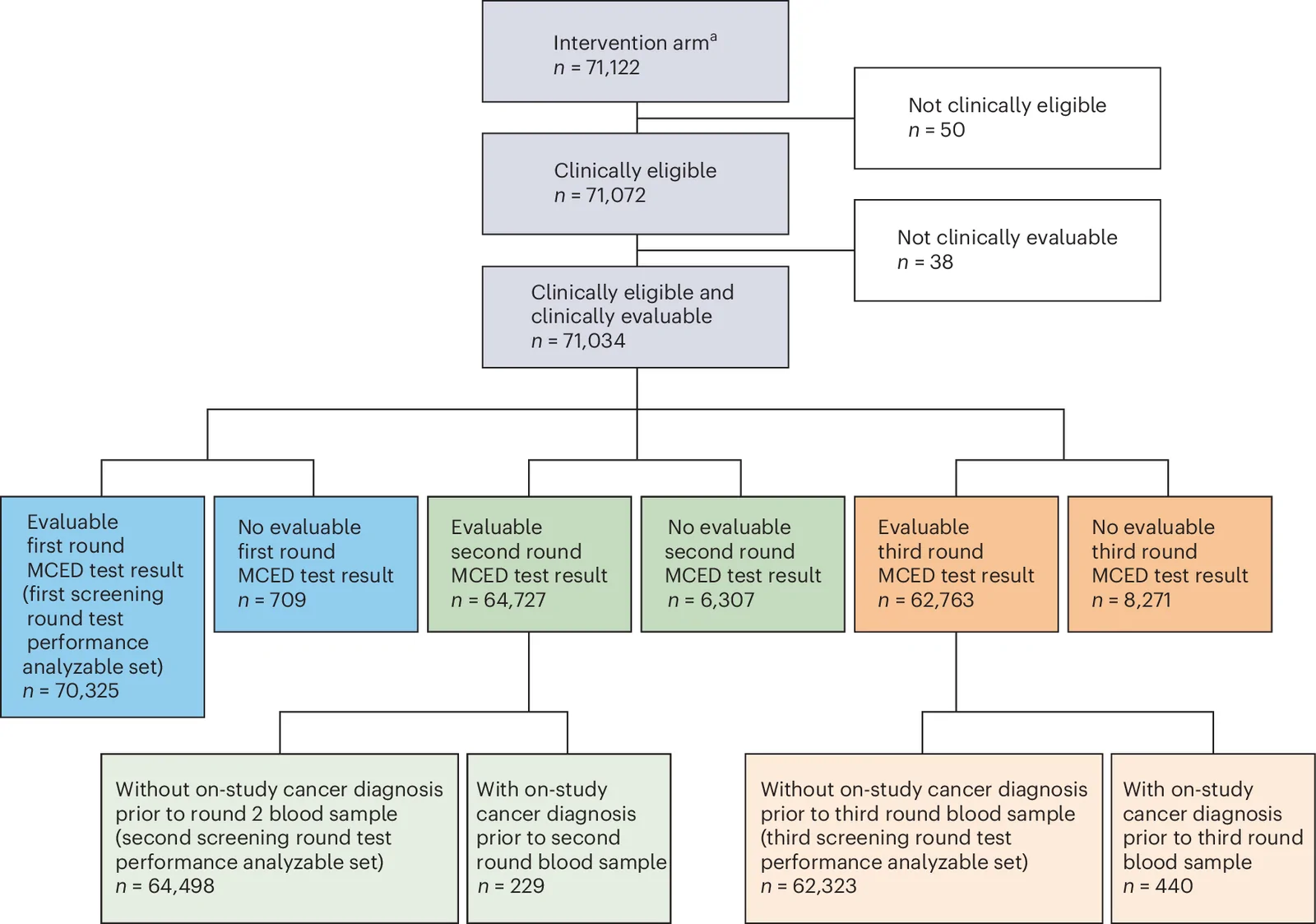
Participant disposition for round-specific test performance analysis sets. Clinically eligible participants successfully traced in the NCRAS were included in the clinically eligible and clinically evaluable analysis set. Participants who were not successfully traced in the NCRAS were excluded from the clinically eligible and clinically evaluable analysis set. ‘Successfully traced in NCRAS’ was defined as having a confirmed cancer/non-cancer status. ^a^One participant aged 49 years was initially enrolled but was subsequently withdrawn for failure to meet inclusion criteria.

A total of 142,826 participants aged 50−77 years (at invitation; 50−78 years at enrollment) were consented over 10.5 months; 142,250 were randomized after baseline blood collection, with 71,122 assigned to the intervention arm and 71,128 assigned to the control arm (see Fig. 1 for full participant disposition). The test performance analyzable set, defined as intervention arm participants who were clinically eligible and evaluable (that is, evaluable MCED test result and successfully traced in England’s population-based cancer registry—the National Cancer Registration and Analysis Service (NCRAS)—to confirm cancer status), comprised 70,325 participants in the first round, 64,498 in the second round and 62,323 in the third round. Despite the challenges of the global COVID-19 pandemic (trial recruitment between 31 August 2021 and 16 July 2022), the trial consented participants largely reflective of the English population of that age group^27^ with high retention (approximately 88%) across the trial’s three annual screening rounds^26^.

Demographics and baseline characteristics, by screening round, are shown in Table 1. The median age of test performance analyzable participants at enrollment was 66 years (interquartile range (IQR): 59−71), and 35,332 (50.2%) were female. Most participants were White (65,827 (93.6%)), reflecting England’s population of the relevant age group^27^. There was greater representation in the test performance analyzable set from the more deprived quintile of the Index of Multiple Deprivation (IMD) distribution: 22.6% (15,863) were most deprived (quintile 1), and 16.1% (11,348) were least deprived (quintile 5). Most participants reported their smoking status as never smoked (38,574 (54.9%)), and 4,680 (6.7%) reported that they currently smoke. Based on body mass index (BMI) calculated using self-reported height and weight at enrollment, 27,708 (39.4%) participants had overweight (25 to <30 kg m^−^^2^) and 19,492 (27.7%) had obesity (≥30 kg m^−^^2^). Baseline demographics of participants who returned for the second and third screening rounds were broadly similar to those of the first round analyzable set.

**Table 1:** Demographics and baseline characteristics of participants included in the MCED test performance analyzable set by screening round

	First screening round set (*N* = 70,325)	Second screening round set (*N* = 64,498)	Third screening round set (*N* = 62,323)
Age at baseline (years)			
Mean (s.d.)	65.0 (7.6)	65.1 (7.5)	65.0 (7.5)
Median (IQR)	66 (59−71)	66 (59−71)	66 (59−71)
Min, Max	50, 78	50, 78	50, 78
Age group, *n* (%)			
50−59 years	18,592 (26.4)	16,610 (25.8)	16,173 (26.0)
60−69 years	28,246 (40.2)	26,251 (40.7)	25,490 (40.9)
70−79 years	23,487 (33.4)	21,637 (33.5)	20,660 (33.1)
Biological sex			
Female	35,332 (50.2)	32,558 (50.5)	31,551 (50.6)
Male	34,993 (49.8)	31,940 (49.5)	30,772 (49.4)
Race/ethnicity (UK), *n* (%)			
White	65,827 (93.6)	60,760 (94.2)	58,643 (94.1)
Mixed or multiple ethnic groups	765 (1.1)	640 (1.0)	640 (1.0)
Black, African, Caribbean or Black British	1,003 (1.4)	804 (1.2)	794 (1.3)
Asian or Asian British	2,334 (3.3)	1,955 (3.0)	1,913 (3.1)
Other ethnic group	311 (0.4)	250 (0.4)	249 (0.4)
Missing	85 (0.1)	89 (0.1)	84 (0.1)
IMD^a^ (quintile), *n* (%)			
1 (most deprived)	15,863 (22.6)	14,030 (21.8)	13,547 (21.7)
2	13,780 (19.6)	12,531 (19.4)	12,059 (19.3)
3	14,887 (21.2)	13,774 (21.4)	13,350 (21.4)
4	14,215 (20.2)	13,285 (20.6)	12,840 (20.6)
5 (least deprived)	11,348 (16.1)	10,675 (16.6)	10,324 (16.6)
Missing	232 (0.3)	203 (0.3)	203 (0.3)
Smoking status at baseline, *n* (%)			
Currently smokes	4,680 (6.7)	3,943 (6.1)	3,690 (5.9)
Formerly smoked	26,984 (38.4)	24,728 (38.3)	23,778 (38.2)
Never smoked	38,574 (54.9)	35,736 (55.4)	34,769 (55.8)
Missing	87 (0.1)	91 (0.1)	86 (0.1)
Alcohol use, *n* (%)			
Heavy drinker	8,726 (12.4)	8,018 (12.4)	7,756 (12.4)
Moderate drinker	22,838 (32.5)	21,168 (32.8)	20,443 (32.8)
Light drinker	23,694 (33.7)	21,923 (34.0)	21,266 (34.1)
Former drinker	10,773 (15.3)	9,705 (15.0)	9,297 (14.9)
Never drinker	4,004 (5.7)	3,408 (5.3)	3,298 (5.3)
Missing	290 (0.4)	276 (0.4)	263 (0.4)
Prior cancer history, *n* (%)			
Yes	5,229 (7.4)	4,746 (7.4)	4,523 (7.3)
No	65,096 (92.6)	59,752 (92.6)	57,800 (92.7)
Number of years since prior cancer diagnosis at baseline^b^			
* N*	4,113	3,748	3,568
Mean (s.d.)	11.49 (5.80)	11.49 (5.78)	11.50 (5.78)
Median (IQR)	10.25 (6.80−15.05)	10.27 (6.82−15.07)	10.30 (6.83−15.06)
Min, Max	3.01, 27.30	3.01, 27.30	3.01, 27.30
Family history of cancer in first-degree relative^c^, *n* (%)			
Yes	41,002 (58.3)	37,742 (58.5)	36,426 (58.4)
No	29,235 (41.6)	26,664 (41.3)	25,810 (41.4)
Missing	88 (0.1)	92 (0.1)	87 (0.1)
BMI category, *n* (%)			
<25 kg m^−^^2^	22,825 (32.5)	21,089 (32.7)	20,408 (32.7)
25 to <30 kg m^−^^2^	27,708 (39.4)	25,359 (39.3)	24,547 (39.4)
≥30 kg m^−^^2^	19,492 (27.7)	17,757 (27.5)	17,090 (27.4)
Missing	300 (0.4)	293 (0.5)	278 (0.4)
Prior history of selected medical conditions, *n* (%)			
Barrett’s esophagus	797 (1.1)	751 (1.2)	711 (1.1)
Chronic bowel disease (for example, Crohn’s disease or ulcerative colitis)	1,636 (2.3)	1,500 (2.3)	1,431 (2.3)
Chronic liver disease (for example, chronic hepatitis infection or fatty liver disease)	1,153 (1.6)	1,013 (1.6)	975 (1.6)
Diabetes	6,998 (10.0)	6,282 (9.7)	6,050 (9.7)
Chronic pancreas disease (for example, pancreatitis)	299 (0.4)	267 (0.4)	256 (0.4)
Systemic lupus erythematosus (SLE, ‘lupus’)	128 (0.2)	114 (0.2)	103 (0.2)
Education, *n* (%)			
School at or before the age of 15	12,537 (17.8)	11,231 (17.4)	10,787 (17.3)
CSEs, O-levels or equivalent^d^	19,051 (27.1)	17,511 (27.1)	16,965 (27.2)
A-levels or equivalent^e^	6,440 (9.2)	5,895 (9.1)	5,671 (9.1)
Further education without a degree	14,610 (20.8)	13,517 (21.0)	13,082 (21.0)
Bachelor’s degree or equivalent	11,836 (16.8)	10,961 (17.0)	10,617 (17.0)
Further degree (for example, masterʼs or PhD)	5,766 (8.2)	5,294 (8.2)	5,117 (8.2)
Missing	85 (0.1)	89 (0.1)	84 (0.1)

^a^IMD measures relative deprivation within small areas of the UK based broadly on income, employment, education, health, crime, barriers to housing and services and the living environment in those areas.

^b^If a participant had multiple prior cancers, the number of years since the most recent prior cancer to randomization date is used.

^c^First-degree relatives include an individualʼs parents, siblings and offspring.

^d^CSEs (Certificates of Secondary Education); O-levels (Ordinary Levels) are the predecessor examinations of the General Certificate of Secondary Education currently used in the UK.

^e^A-levels (Advanced Levels) are the qualifications conferred as part of the General Certificate of Education to students completing secondary, pre-university education.

### MCED test results, outcomes and performance

Performance metrics are presented from the prevalent (first) screening round, followed by the two incident (second and third) screening rounds. Cancer detection in the prevalent screening round is influenced by the pre-existing, undiagnosed cancers in the population for which participants may have never been previously screened^28^. Cancer detection in incident screening rounds is more reflective of what would be expected with a stable annual population screening program, with detected cancers having developed or become detectable in the interval since the previous screen or having been missed by the previous screen or diagnostic follow-up. Aggregate results across all three screening rounds are included in tables and figures.

The cancer signal detection rate was 1.03% (95% confidence interval (CI): 0.95−1.10%) in the first round (722/70,325 participants had a positive MCED test result; Table 2). This decreased to 0.80% (518/64,498, 95% CI: 0.74−0.87%) in the second round and to 0.90% (561/62,323, 95% CI: 0.83−0.98%) in the third round. Results for the second and third rounds excluded participants who had cancer previously diagnosed during the trial.

**Table 2:** MCED test performance in the MCED test analyzable set by screening round and aggregate

		Round of screening							
		First (*N* = 70,325)		Second (*N* = 64,498)		Third (*N* = 62,323)		Aggregate^a^ (*N* = 197,146)	
Clinical outcome									
		Cancer	No cancer	Cancer	No cancer	Cancer	No cancer	Cancer	No cancer
MCED test result	Positive	419	303	261	257	257	304	937	864
	Negative	708	68,895	702	63,278	704	61,058	2,114	193,231
Performance metrics									
Cancer signal detection rate^b^, % (*n*/*N*) (95% CI)		1.03 (722/70,325) (0.95−1.10)		0.80 (518/64,498) (0.74−0.87)		0.90 (561/62,323) (0.83−0.98)		0.91 (1,801/197,146) (0.87−0.96)	
Cancer detection rate^c^, % (*n*/*N*) (95% CI)		0.60 (419/70,325) (0.54−0.66)		0.40 (261/64,498) (0.36−0.46)		0.41 (257/62,323) (0.37−0.47)		0.48 (937/197,146) (0.45−0.51)	
Number needed to screen^d^, *n* (95% CI)		168 (153−185)		247 (219−279)		243 (215−274)		210 (197−224)	
PPV, % (*n*/*N*) (95% CI)		58.0 (419/722) (54.4−61.6)		50.4 (261/518) (46.1−54.7)		45.8 (257/561) (41.7−49.9)		52.0 (937/1,801) (49.7−54.3)	
NPV, % (*n*/*N*) (95% CI)		98.98 (68,895/69,603) (98.91−99.05)		98.90 (63,278/63,980) (98.82−98.98)		98.86 (61,058/61,762) (98.77−98.94)		98.92 (193,231/195,345)(98.87−98.96)	
Specificity, % (*n*/*N*) (95% CI)		99.56 (68,895/69,198) (99.51−99.61)		99.60 (63,278/63,535) (99.54−99.64)		99.50 (61,058/61,362) (99.45−99.56)		99.55 (193,231/194,095) (99.52−99.58)	
False-positive rate, % (*n*/*N*) (95% CI)		0.44 (303/69,198) (0.39−0.49)		0.40 (257/63,535) (0.36−0.46)		0.50 (304/61,362) (0.44−0.55)		0.45 (864/194,095) (0.42−0.48)	
Episode sensitivity, % (*n*/*N*) (95% CI)		37.2 (419/1,127) (34.4−40.0)		27.1 (261/963) (24.4−30.0)		26.7 (257/961) (24.0−29.6)		30.7 (937/3,051) (29.1−32.4)	
Positive likelihood ratio^e^ (95% CI)		84.9 (74.1−97.2)		67.0 (57.1−78.6)		54.0 (46.3−62.9)		69.0 (63.4−75.1)	
Negative likelihood ratio^f^ (95% CI)		0.63 (0.60−0.66)		0.73 (0.70−0.76)		0.74 (0.71−0.76)		0.70 (0.68−0.71)	

^a^Aggregate calculations were based on all evaluable MCED test results all three rounds of screening. *N*=197,146 is the number of evaluable tests, not the number of unique participants.

^b^Cancer signal detection rate was defined as the proportion of positive MCED tests out of all tests in a given screening round.

^c^The cancer detection rate was the proportion of participants with true positives out of all tests in a given screening round.

^d^Number needed to screen to detect one cancer was the inverse of the cancer detection rate.

^e^The positive likelihood ratio is the ratio of the probability that a participant with cancer diagnosis has a positive test result versus the probability that a participant with no cancer diagnosis has a positive test result.

^f^The negative likelihood ratio is the ratio of the probability that a participant with cancer diagnosis has a negative test result versus the probability that a participant with no cancer diagnosis has a negative test result.

Among the 722 positive MCED test results in the first round, 419 (58.0%) were true-positive results with a primary cancer diagnosed during the follow-up window (Table 2), yielding a cancer detection rate of 0.60% (419/70,325, 95% CI: 0.54−0.66%) and a corresponding number needed to screen to detect one primary cancer of 168 (95% CI: 153−185). In the second and third rounds, the cancer detection rates were lower at 0.40% (261/64,498, 95% CI: 0.36−0.46%) and 0.41% (257/62,323, 95% CI: 0.37−0.47%), corresponding to numbers needed to screen of 247 (95% CI: 219−279) and 243 (95% CI: 215−274), respectively.

PPV was 58.0% (95% CI: 54.4−61.6%) in the first round when calculated based on primary cancers only (See ‘Exploratory analyses’ section below for metrics including recurrent cancers). PPV was 50.4% (95% CI: 46.1−54.7%) in the second round and 45.8% (95% CI: 41.7−49.9%) in the third round. For each of the three rounds, specificity was 99.56% (95% CI: 99.51−99.61%), 99.60% (95% CI: 99.54−99.64%) and 99.50% (95% CI: 99.45−99.56%), corresponding to a false-positive rate of 0.44% (95% CI: 0.39−0.49%), 0.40% (95% CI: 0.36−0.46%) and 0.50% (95% CI: 0.44−0.55%), respectively. Negative predictive value (NPV) was 98.98% (95% CI: 98.91−99.05%) in the first round, 98.90% (95% CI: 98.82−98.98%) in the second round and 98.86% (95% CI: 98.77−98.94%) in the third round (Table 2).

Episode sensitivity, defined as the proportion of participants diagnosed with a primary cancer within the prespecified follow-up window (within 12 months of the corresponding blood collection) who had a positive MCED test result for that screening episode, was 37.2% (95% CI: 34.4−40.0%) in the first round, 27.1% (95% CI: 24.4−30.0%) in the second round and 26.7% (95% CI: 24.0−29.6%) in the third round (Table 2). Episode sensitivity was also calculated for a subgroup of 12 prespecified cancer types responsible for more than 60% of cancer deaths^14,25^ (anus, bladder, colon/rectum, esophagus, head and neck, liver/bile duct, lung, lymphoma, ovary, pancreas, plasma cell neoplasm (myeloma) and stomach). For this subgroup, episode sensitivity was 63.4% (95% CI: 58.9−67.7%), 47.6% (95% CI: 42.5−52.8%) and 50.7% (95% CI: 45.6−55.7%) in the first, second and third rounds, respectively (Supplementary Table 1). Across all individual cancer types, aggregate episode sensitivity for the three rounds ranged from 88.9% (32/36, 95% CI: 74.7−95.6%) for liver/bile duct cancers to 2.5% (4/157, 95% CI: 1.0−6.4%) for melanoma (Supplementary Table 1 and Supplementary Fig. 1).

**Fig. 2:**
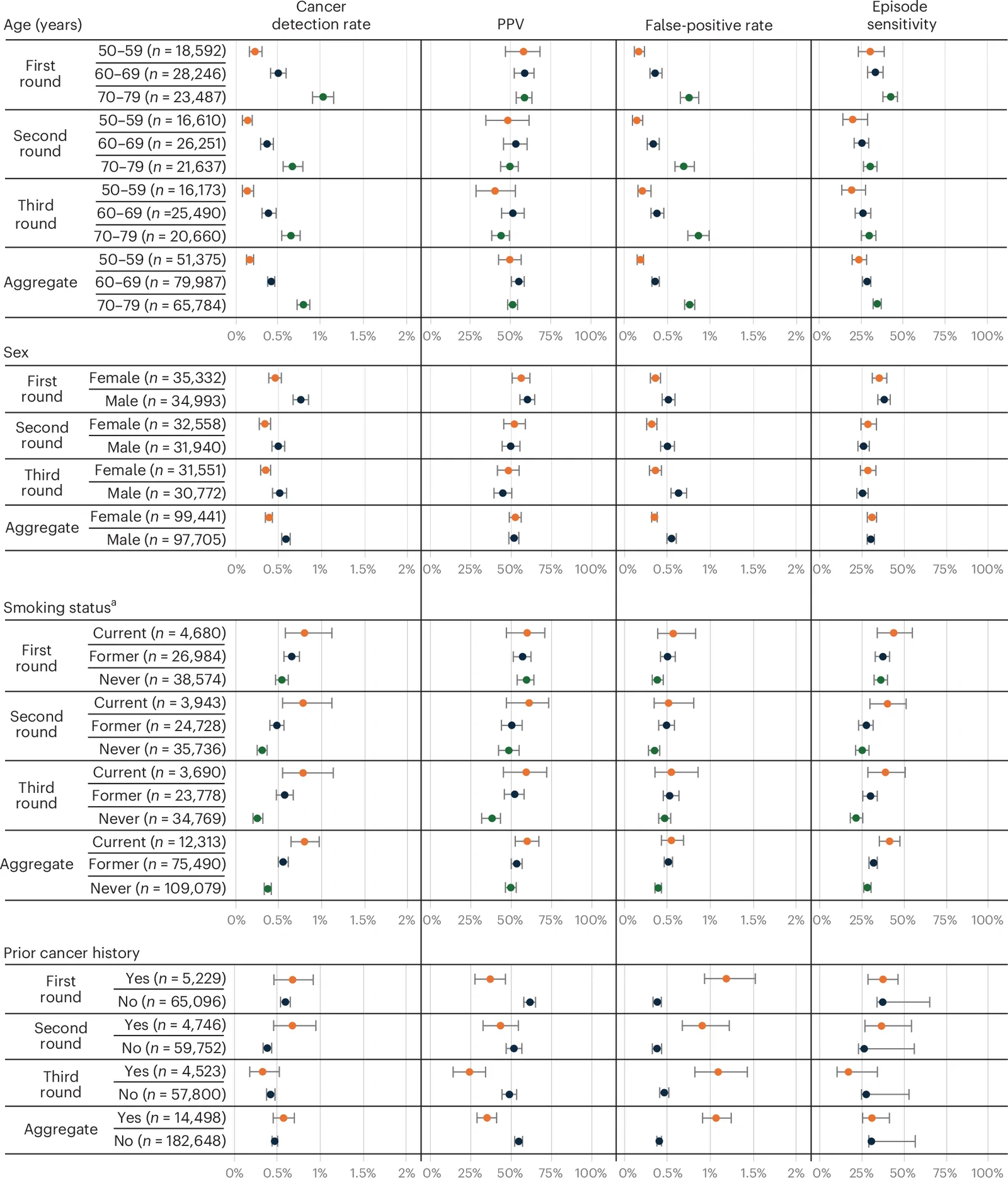
Forest plot for the subgroup analysis on test performance metrics by round and in aggregate. Performance metric data are presented as observed percentages for each subgroup; error bars denote two-sided 95% CIs. Performance metric estimates were calculated as defined in Supplementary Table 15 based on MCED test results and new cancer diagnoses received within the follow-up period for that round. The *n*/*N* used to calculate the estimates can be found in Supplementary Table 2 for Subgroup: Age; in Supplementary Table 3 for Subgroup: Sex; in Supplementary Table 4 for Subgroup: Smoking Status; and in Supplementary Table 5 for Subgroup: Prior Cancer History. Subgroup categories (shown on the *y* axis) are based on participant characteristics at the time of enrollment. ^a^Participants with missing smoking category are not included in calculations (87 participants in round 1, 91 participants in round 2 and 86 participants in round 3).

Exploratory prespecified subgroup analyses by age, sex, smoking status, prior cancer history, race/ethnicity, IMD quintile and education were conducted (Fig. 2 and Supplementary Tables 2–8). The aggregate cancer detection rate increased with age (at enrollment) from 0.18% (95% CI: 0.14−0.22%) for participants aged 50−59 years to 0.78% (95% CI: 0.71−0.85%) in those aged 70−79 years; the age-specific aggregate false-positive rate also increased with age from 0.18% (95% CI: 0.15−0.22%) to 0.76% (95% CI: 0.70−0.83%) (Fig. 2 and Supplementary Table 2). Compared to female participants, male participants had a higher aggregate cancer detection rate (0.58% (95% CI: 0.53−0.63%) versus 0.38% (95% CI: 0.34−0.42%)) and a higher aggregate false-positive rate (0.55% (95% CI: 0.50−0.60%) versus 0.35% (95% CI: 0.31−0.38%)) (Fig. 2 and Supplementary Table 3). Thus, the PPV was relatively homogeneous across age groups and sex. Aggregate cancer detection rate and episode sensitivity were higher for participants who currently smoke than for those who never smoked, although imbalance in the subgroup sizes should be noted (Fig. 2 and Supplementary Table 4). Aggregate PPV was lower and false-positive rate was higher for participants with prior cancer history compared to those without prior cancers, but these metrics were calculated based on primary cancers, and differences were reduced when MCED-detected recurrent cancers were included as true positives (see ‘Exploratory analyses’ section; Fig. 2 and Supplementary Table 5).

**Fig. 3:**
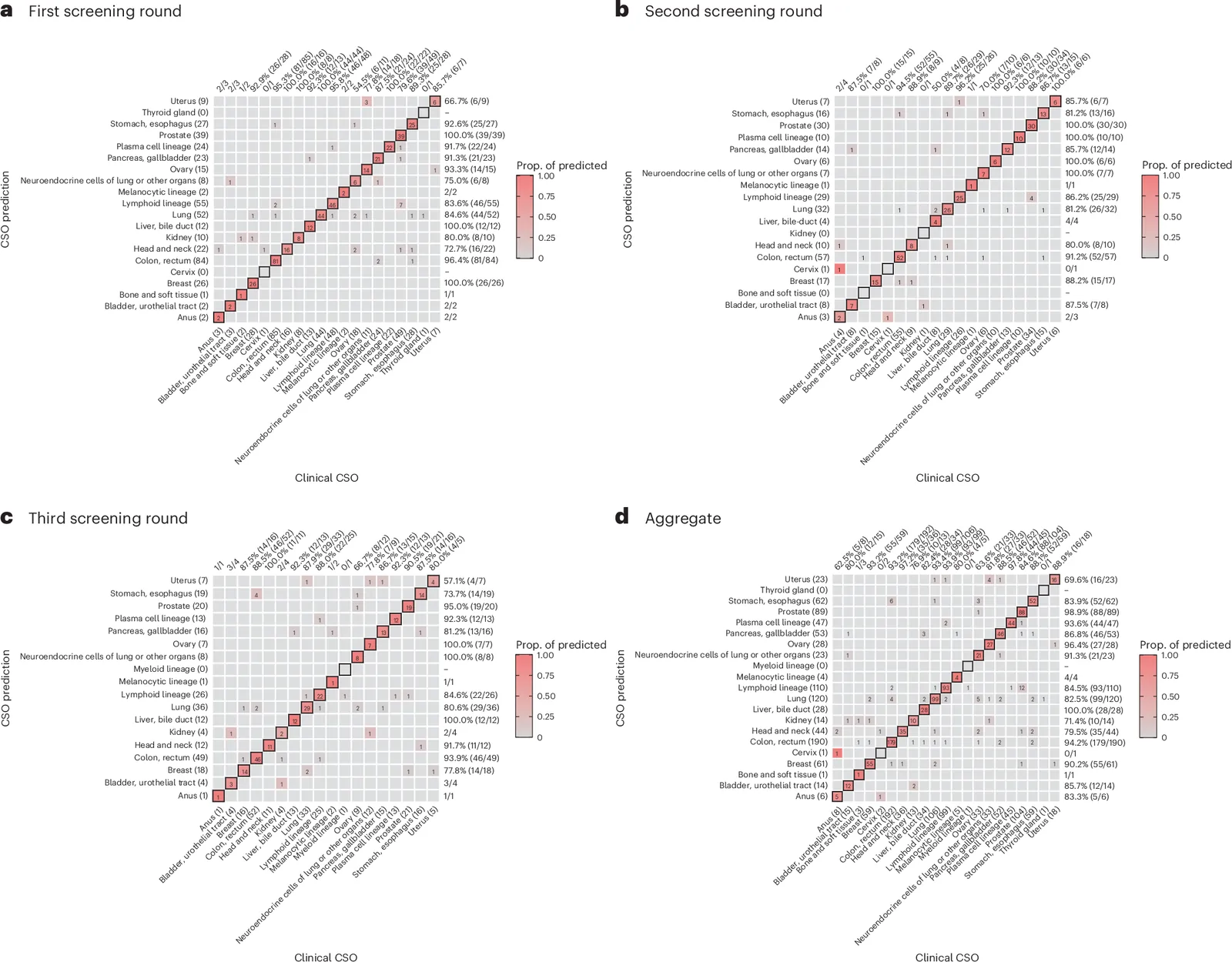
Confusion matrix predicted CSO versus clinical CSO by round and in aggregate. **a**, First screening round. **b**, Second screening round. **c**, Third screening round. **d**, Aggregate. The confusion matrix for each round and in aggregate plots the number of participants for each combination of CSO prediction (*y* axis) and clinical CSO (*x* axis). Participants diagnosed with cancer types without CSOs and unassigned clinical CSO (NCRAS CUP) are not visualized. Participants with multiple primary cancers are included in the confusion matrix if one cancer has an assigned clinical CSO. Diagonal cells indicate correct CSO1 assignments (accuracy), and off-diagonal cells indicate misclassifications (errors). CSO1 prediction accuracy by clinical CSO (estimated by the proportion of the diagonal cell in each column) is indicated at the top of the matrix, and precision of each CSO prediction (estimated by the proportion of the diagonal cell in each row) is indicated along the right side. Accuracy and precision are not estimated for subgroups with fewer than five cases. The color is associated with the proportions of matched predicted and clinical CSOs. Only CSOs with at least one prediction or diagnosed cancer are included in each matrix. CUP, cancer of unknown primary; Prop, proportion.

Predicted CSOs are returned with positive MCED test results to guide diagnosis by providing the anatomical site or cellular lineage most likely to be the source of the detected cancer signal. In the first round, a correct first or second CSO (CSO1/CSO2) prediction was returned for 93.6% (392/419, 95% CI: 90.8−95.5%) of participants with true-positive test results. CSO1/CSO2 prediction accuracy was consistent across rounds: 92.3% (241/261, 95% CI: 88.5−95.0%) in the second round and 91.1% (234/257, 95% CI: 86.9−94.0%) in the third round (Fig. 3 and Table 3). In aggregate across the three screening rounds, the accuracy for CSO1 was 87.0% (815/937, 95% CI: 84.7−89.0%); the accuracy for CSO2 prediction was 46.8% (52/111, 95% CI: 37.8−56.1%) for true-positive test results with two CSOs returned when CSO1 was inaccurate. CSO performance was also consistent by sex across rounds (Supplementary Fig. 2).

**Table 3:** CSO accuracy for participants with true-positive results and ≥1 CSO prediction returned: summary by screening round and in aggregate

Category	Participants with true-positive results and ≥1 CSO prediction returned			
	First screening round (*N* = 419)	Second screening round (*N* = 261)	Third screening round (*N* = 257)	Aggregate (*N* = 937)
Accuracy of CSO1/CSO2 prediction, %, (*n*/*N*), (95% CI)	93.6 (392/419) (90.8−95.5)	92.3 (241/261) (88.5−95.0)	91.1 (234/257) (86.9−94.0)	92.5 (867/937) (90.7−94.0)
Accuracy of CSO1 prediction, %, (*n*/*N*), (95% CI)	89.0 (373/419) (85.7−91.7)	85.8 (224/261) (81.1−89.5)	84.8 (218/257) (79.9−88.7)	87.0 (815/937) (84.7−89.0)
Participants with two CSOs returned, *n* (%)	237 (56.6)	170 (65.1)	179 (69.6)	586 (62.5)
Participants with two CSOs and inaccurate CSO1 prediction, *n* (% of those with two CSOs)	42 (17.7)	33 (19.4)	36 (20.1)	111 (18.9)
Accuracy of CSO2 prediction when CSO1 was inaccurate, %, (*n*/*N*), (95% CI)^a^	45.2 (19/42) (31.2−60.1)	51.5 (17/33) (35.2−67.5)	44.4 (16/36) (29.5−60.4)	46.8 (52/111) (37.8−56.1)

^a^CSO2 accuracy was calculated only among true-positive cases in which CSO1 was inaccurate and a second CSO was returned. In aggregate, of 122 true-positive cases with inaccurate CSO1, 111 had a CSO2 returned.

PPV stratified by CSO prediction varied across CSO predictions and by round (Supplementary Fig. 3 and Supplementary Table 9). This was typically a result of small numbers of some CSO predictions leading to unstable point estimates and wide CIs. In aggregate across all three screening rounds, the highest PPVs were generally observed for CSOs corresponding to the subgroup of 12 prespecified cancer types, including ovary (93.3%), stomach/esophagus (64.6%), pancreas/gallbladder (64.3%) and liver/bile duct (63.8%); neuroendocrine also had very high PPV (82.8%). The lowest PPV was for bone and soft tissue, which was based on just five samples with that CSO and one actual diagnosis (20.0%, 95% CI: 3.6−62.4%) (Supplementary Fig. 3 and Supplementary Table 9).

### Diagnosed cancer stage and type

Among the 419 participants with MCED-detected cancers in the first round, most were diagnosed prior to stage IV disease: stage I−II, 166 (39.6%); stage I−III, 289 (69.0%) (with 27 (6.4%) being either unstageable or having missing stage) (Supplementary Table 10). There were 261 participants with MCED-detected cancers in the second round, with 105 (40.2%) being diagnosed with stage I−II cancers and 180 (69.0%) being diagnosed with stage I−III cancers. Among the 257 participants with MCED-detected cancers in the third round, 95 (37.0%) were diagnosed with stage I−II cancers and 171 (66.5%) with stage I−III cancers. In aggregate, 366 of 937 (39.1%) of MCED-detected cancers were diagnosed at stage I−II and 640 of 937 (68.3%) at stage I−III.

In total, of the 937 participants with at least one MCED-detected cancer, 646 (68.9%) were of the 12 prespecified cancer types. The most common MCED-detected cancer types were colon/rectum (186 (19.9%)), lung (127 (13.6%)), prostate (103 (11.0%)), lymphoma (71 (7.6%)), breast (56 (6.0%)), plasma cell neoplasm (43 (4.6%)), esophagus (42 (4.5%)), pancreas (36 (3.8%)), ovary (34 (3.6%)), liver/bile duct (32 (3.4%)) and head and neck (32 (3.4%)) (Supplementary Table 1, in which anyone with more than one cancer diagnosis was classified as ‘multiple cancers’). The proportion of true-positive MCED test results attributable to lung cancer increased slightly across rounds, from 11.7% (49/419) in the first round to 13.8% (36/261) in the second round and to 16.3% (42/257) in the third round, despite broadly stable absolute numbers. A prespecified descriptive summary of the 937 first primary cancers detected by the MCED test across all three rounds (Supplementary Table 10, in which anyone with more than one cancer diagnosis was classified according to their first diagnosis) showed that most were types that do not have recommended screening programs in the UK, with 682 of 937 (72.8%) MCED-detected cancers excluding breast, cervix and colon/rectum and 550 of 937 (58.7%) excluding breast, cervix, colon/rectum and lung. In a post hoc analysis based on American Joint Committee on Cancer (AJCC) classifications^29^, 41 unique cancer types were diagnosed after a positive MCED test result (Supplementary Table 11).

Among the 708 participants with false-negative test results in the first round, 478 of 708 (67.5%) had stage I−II disease diagnosed within 12 months of testing (Supplementary Table 12). Similarly, most participants with false-negative test results in the second and third rounds were eventually diagnosed with stage I−II disease (462/702 (65.8%) and 473/704 (67.2%), respectively). Among all 2,114 participants with false-negative test results across all three rounds, the most common types of cancers with which they were diagnosed were prostate (713 (33.7%)), breast (301 (14.2%)), melanoma (153 (7.2%)), colon/rectum (130 (6.1%)) and lung (126 (6.0%)) (Supplementary Fig. 4 and Supplementary Table 1).

### Exploratory analyses

A caveat with the PPV analysis in this study is that the NCRAS registers only primary cancers, such that positive MCED test results for those diagnosed with recurrent cancers were classified as false positive. Although only 7.4% of participants had a prior cancer history, a sensitivity analysis for PPV was conducted that included recurrent cancers in those with a positive MCED test result. Of participants with false-positive test results when considering only primary cancers, 5.6% (17/303) were determined to have recurrent cancers in the first round, 2.7% (7/257) in the second round and 3.9% (12/304) in the third round. After reclassifying adjudicated recurrences from false positive to true positive (Methods), PPV increased from 58.0% (419/722) to 60.4% (436/722) in the first round, from 50.4% (261/518) to 51.7% (268/518) in the second round and from 45.8% (257/561) to 48.0% (269/561) in the third round (Supplementary Table 13). The aggregate PPV in participants with a prior cancer history rose from 34.9 (95% CI: 29.1−41.2%) to 50.4% (95% CI: 44.0−56.8%) with the inclusion of recurrences, which is similar to the PPV in participants without prior cancer history (54.6% (95% CI: 52.1−57.0%)) (Supplementary Tables 5 and 13).

In a post hoc analysis of participants with results classified as false positive in the first round, 17.8% (54/303) were subsequently diagnosed with cancer during the second or third round follow-up windows, including 36 cases in the second round and 18 cases in the third round (Supplementary Table 14). Of these, 81.5% (44/54) had cancers concordant with the first round CSO1/CSO2 prediction and 75.9% (41/54) with the CSO1 prediction, meaning that only 3.3% of the participants with false-positive test results (10/303) were later diagnosed with a cancer discordant with the original CSO prediction. Similar patterns were observed for participants with false-positive test results in the second round: 9.7% (25/257) of participants who received false-positive results in the second round were diagnosed with cancer in the third round, and 72.0% (18/25) of these participants had received a correct CSO1/CSO2 prediction (Supplementary Table 14).

## Discussion

NHS-Galleri is a randomized controlled trial evaluating an MCED test when added to standard-of-care cancer screening across multiple rounds of annual screening, with high retention throughout the trial, allowing for comparison of performance across prevalent and incident screening rounds.

The MCED test demonstrated performance with first (prevalent) round metrics consistent with other prospective clinical studies of this test that had only a single screening round and were performed in different health systems^15,16^. PATHFINDER 2, a large, prospective, interventional study of the MCED test in North America, showed similar cancer detection rates (0.54%), PPV (60.3%), NPV (99.2%), 12-month episode sensitivity (39.3%) and specificity (99.64%)^16^. The consistent performance of this MCED test across two different health systems in England and North America is notable, especially given the limited number of prospective studies of any MCED test.

In NHS-Galleri, the MCED test aggregate PPV (52.0%) was higher than the proportion of urgent 2-week wait referrals for suspected cancers in England that resulted in a cancer diagnosis (6%)^30^. Furthermore, the majority (68%) of MCED-detected cancers were diagnosed at stages I−III. This comparably higher proportion of diagnoses and at earlier stages with the MCED test is notable given that urgent referrals rely on red flag symptoms, with approximately 40% of new cancer diagnoses in rapid diagnostic clinics being diagnosed at stage IV^31^. The aggregate false-positive rate (0.45%) for the MCED test was also below what has been reported for single-cancer screening tests, which range from 0.98% to 19.9%^32–35^. Improvements in the MCED test’s sensitivity, particularly for early-stage cancers and cancer types with lower observed sensitivity, could increase the potential clinical benefit of MCED screening. However, any gains in sensitivity would need to preserve the high specificity required for population screening and be evaluated for their effects on overdiagnosis. The MCED test performance results from NHS-Galleri reported here should be interpreted alongside the trial’s randomized clinical utility analyses and evaluation of safety, including adverse events, reported in Sasieni et al.^26^. In brief, this paper reports that a low rate of participants (<1%) experienced adverse events and also discusses the potential impact of lead time on results and the need for additional time before any mortality endpoint can be assessed.

Both CSO1/CSO2 and CSO1 predictions in NHS-Galleri were accurate (>91% and >84%, respectively), indicating that the MCED test offers information that can be used to guide diagnostic evaluation. A previous study of this MCED test showed that using predicted CSO(s) to guide diagnostic referrals could result in potentially meaningful improvements in diagnostic efficiency for 19% of all cancers^36^. It should be noted that, even in the cases for which CSO1 was not accurate, the diagnosed cancer was often for a related or adjacent tissue, such as a colon/rectum prediction for stomach/esophagus or a uterus prediction for ovary, supporting the biological driver of the signal.

As expected, the PPV, cancer detection rate and episode sensitivity decreased in the second and third (incident) rounds. In the first, prevalent round of screening, the population is naive to MCED screening, and there is a prevalent pool of pre-existing undetected cancers available for detection^28,37^. Subsequent incident rounds are expected to capture cancers that become detectable during the inter-screening interval, including emergent cancers and those missed by prior screenings or diagnostic workup, which may be less advanced or of types more difficult to detect. Changes in performance across rounds have been reported in other multi-round screening studies^38,39^. Performance metrics such as specificity, which is estimated among participants without cancer, and CSO accuracy, which is estimated only in those with an MCED-detected cancer, remained consistent across rounds, as these are less affected by cancer prevalence.

The performance metrics were generally consistent across demographic subgroups. PPV was similar across age, sex, race/ethnicity, quintile of IMD, education and prior history of cancer (once recurrent cancers were included in the metric calculations for those with a prior cancer history). There were some expected differences, such as higher cancer detection rates in older age groups, males, participants who currently smoke or formerly smoked and those with a prior cancer history, driven by higher cancer risk in these groups^1,3,40,41^. The aggregate false-positive rate increased with age but remains low for each group, in absolute terms. The biological source of this signal remains uncertain; however, it is important to note that the PPV, which reflects the likelihood that a positive MCED test result represents a true cancer detection, remains high and robust across all age groups. Similarly, although male participants also had a higher proportion of false-positive test results than female participants, in absolute terms the proportion was low and the PPV was similar in male and female participants. The increased episode sensitivity in participants who currently smoke is likely to be reflective of the more adverse mix of types and stage of cancers seen in this population. This also likely contributed to the increase in PPV in this population and highlights that MCED testing may help address an unmet need for non-lung cancer screening in participants at elevated risk of cancer due to smoking. It should be noted that the proportion of participants who currently smoke and the proportion who never smoked are slightly lower than those in the general UK population^27^, which might indicate a degree of volunteer bias and could impact the interpretation of performance across subgroups. Further analyses into differing MCED test performance and cancer detection rates between subgroups may provide insight into the complex risk stratification in these populations.

Among participants with MCED-detected cancer, 41 distinct AJCC cancer types were diagnosed. As would be expected, the most frequently MCED-detected cancers included common types of cancer in England, such as breast, prostate, lung and colon/rectum^3^. Many cancer types with recommended screening programs were detected, but most were types that do not have recommended screening programs in the UK. Episode sensitivity varied by cancer type, and a negative MCED test result should not be interpreted as ruling out any particular cancer. MCED testing is intended to complement, not replace, existing screening programs.

The study strengths include the large number of participants, the high retention across rounds and demographic characteristics that were generally similar to England’s population of the relevant age group^27^. Conducting the trial within the NHS, with positive MCED test results managed through standard urgent suspected pathways rather than protocol-directed pathways, demonstrated the feasibility of integrating MCED screening into routine care. Tracing of participants through the NCRAS to determine participant cancer status also allowed for timely and complete data, including route to cancer diagnosis (see Sasieni et al.^26^).

Limitations of this study include the trial period overlapping with the (end of the) global COVID pandemic, which may have influenced access to and intensity of diagnostic workup. Because participants with a positive MCED test result were referred through NHS urgent referral pathways for diagnostic workup outside the trial, details of the workup and the date of diagnostic resolution were neither prospectively specified nor systematically ascertained for all participants, precluding reliable comparisons of time to diagnostic resolution. Although participants self-reported high levels of screening attendance^26^, there was no collection of individual screening attendance data. This means that MCED-detected cancers for which recommended screening programs exist could not be characterized as either interval cancers or cancers for which a participant had not been screened due to ineligibility for or non-attendance at NHS screening programs. The NCRAS records only primary and not recurrent cancers, and registry stage may correspond to either clinical stage or pathological stage. Episode sensitivity rather than absolute sensitivity of the MCED test is calculated, as a participant’s cancer status is not known at the time of MCED testing, and so it is impossible to know if a cancer was present and not detected or had developed in the interval between testing and diagnosis. Also, episode sensitivity by stage is not calculated, as cancers could progress between MCED testing and diagnosis, making such estimates biased^42^.

Performance metrics reported here are limited to 12 months of follow-up. Previous studies of this MCED test^36^ and exploratory analysis of participants with false-positive test results in this trial showed that some participants were diagnosed with cancer more than 1 year after a positive MCED test result, suggesting that longer periods of follow-up may be informative. Longer-term follow-up data would also be important to demonstrate the potential morbidity and mortality impact of blood-based cancer detection more generally. Further follow-up will reveal whether blood-based testing can detect cancers early enough to affect longer-term clinical outcomes^43,44^. Retrospective MCED testing of stored control arm blood samples may similarly provide insight into performance calculated with longer follow-up.

Overall, the data presented here illustrate the performance of the MCED test across three annual screening rounds and across subgroups within the NHS in England. These findings contribute to the growing body of clinical evidence on this MCED test in intended-use populations.

## Methods

### Ethical approval and consent

The trial was conducted in accordance with the Declaration of Helsinki, Council for International Organizations of Medical Sciences International Ethical Guidelines, applicable International Council for Harmonization Good Clinical Practice guidance and relevant laws and regulations. The protocol was approved by Wales Research Ethics Committee 1 (ref: 21/WA/0141). Health Research Authority approval was granted with support from the Confidentiality Advisory Group (ref: 21/CAG/0056), under Regulation 5 of the Health Service Regulations 2002 (‘Section 251’ support). An independent data monitoring committee oversaw trial progress, reviewed clinical and safety data and provided recommendations to the sponsor regarding trial conduct. All participants provided written informed consent.

### Trial design

NHS-Galleri (NCT05611632; ISRCTN91431511) was a prospective, randomized controlled trial of the Galleri (GRAIL Inc.) MCED test, enrolling adults aged 50−77 years from eight identified NHS Cancer Alliance regions of England.

Inclusion criteria:

- Aged 50−77 years, inclusive, at the time of data extraction from NHS datasets or general practitioner records used to identify potential participants; and
- Capable of giving signed and legally effective informed consent, which includes compliance with the requirements and restrictions listed in the informed consent form and in the trial protocol. Consent provided by a legally authorized representative was not permitted in this protocol.

Exclusion criteria:

- Previous or current participation in another GRAIL-sponsored study.
- Personal history of invasive cancer or hematologic malignancy, diagnosed within the 3 years prior to expected enrollment date. Individuals with a diagnosis of non-melanoma skin cancer or prostate cancer whose only treatment was active surveillance were not excluded.
- Definitive treatment for invasive cancer or hematologic malignancy within the 3 years prior to expected enrollment date, including adjuvant hormone therapy for cancer (for example, for breast or prostate cancer).
- Currently taking demethylating or cytotoxic agents for any condition.
- Undergoing current investigation for suspected cancer, defined as having been referred to a 2-week wait clinic or undergoing investigations at a rapid diagnostic clinic or other clinic with a stated suspicion of cancer.
- Currently on a palliative care pathway.

Invitations were sent primarily through centralized health service lists targeting older and more deprived groups, with some being invited through their general practice surgery (primary care provider), as previously described^25,27^. Other individuals self-referred, many after community engagement events. The trial protocol and statistical analysis plan with amendments are published as supplemental material in Sasieni et al.^26^. Additionally, more details on the trial design, including recruitment approaches and objectives, were previously published^25,27^.

### Procedures

Participants were enrolled between 31 August 2021 and 16 July 2022. Appointments were coordinated in mobile clinics by EMS Healthcare Ltd. Written informed consent was obtained from each participant prior to their first blood sample. One-to-one randomization to either the intervention arm (blood tested with the MCED test) or the control arm (blood stored) occurred after the first blood sample collection. Allocation was concealed at the point of randomization, and both participants and study staff were initially blinded. After baseline blood collection, all participants were invited to two additional blood sample appointments, each approximately 12 months apart (±6 weeks). Unblinded trial nurses notified only intervention arm participants with a positive MCED test result, who were then referred for diagnostic testing via NHS urgent referral pathways, guided by the test’s predicted CSO(s). Thus, these participants were unblinded for the rest of the trial. As diagnostic procedures were outside the scope of the trial protocol, secondary care clinicians followed local, site-specific guidelines for suspected symptomatic cancer, exercising clinical judgment where necessary^45^. As the trial aimed to assess MCED testing added to standard of care, participants in both arms were encouraged to continue routine NHS cancer screenings for which they were eligible and to contact their general practitioner if new symptoms arose.

Additional details on trial procedures for blood collection, 1:1 randomization to the intervention or control arm, blinding, follow-up and post-MCED test result pathways were previously published^25,45^.

### Data collection

This work uses data provided by patients and collected by the NHS as part of their care and support. The data are collated, maintained and quality assured by the NCRAS, which is part of NHS England. The trial outcome data, including cancer diagnoses, staging and mortality, were obtained from NHS sources by the NCRAS. Cancer diagnosis data were passively collected for all participants throughout the trial, regardless of their attendance at subsequent blood collections, unless they withdrew from the passive follow-up. Registry stage at diagnosis was provided in line with the NCRAS’s remit to capture a comprehensive picture of population cancer burden. This is a single registry-derived stage from all relevant clinical, imaging and pathological information available to the NCRAS for cancer registration, using the applicable tumor, node and metastasis (TNM) or site-specific staging system. Although cancers are not staged under the AJCC system, registry stage is typically equivalent to either AJCC clinical stage or pathological stage. NCRAS processes are consistent with European and international best practices.

Other trial data, including baseline characteristics, blood collection information, MCED test results and test result communication, were collected as part of the clinical trial. Participant demographics and characteristics for subgroup analysis were self-reported age, biological sex, race/ethnicity, IMD as determined by postcode, smoking status, prior cancer history and education. Race/ethnicity categories were self-reported at enrollment and grouped according to guidance from the Office for National Statistics as ‘White’, ‘Black, African, Caribbean or Black British’, ‘Asian or Asian British’, ‘Mixed or multiple ethnic groups’ or ‘Other ethnic group’^46^. Prior cancer history data from the NCRAS were used to supplement self-reported data. The event cutoff date for data included in this paper was 12 July 2025, with a lock date of 28 January 2026 for analysis.

### Study objectives and endpoints

This paper reports the NHS-Galleri trial’s secondary objective and endpoint:

- Evaluate the performance (overall episode sensitivity, specificity, PPV, NPV and CSO accuracy) of the MCED test in the intervention arm after each annual round of testing and in aggregate across the three intervals of testing.

It also reports the exploratory endpoint of subgroup analyses:

- Assess the primary and secondary objectives in clinically meaningful subsets (for example, by age, sex, socioeconomic status, risk factors at enrollment, prior cancer history at enrollment and ethnicity).

Other trial objectives and endpoints not reported here that are reported in Sasieni et al.^26^ include the primary objective and endpoint:

- For cancers that are routinely staged, demonstrate a statistically significant reduction in the incidence rate of stage III and IV cancers diagnosed in the intervention arm as compared to the control arm after three annual tests and an average of 16−18 months of follow-up after the third test, using a fixed-sequence strategy as below:
  - First, evaluate for a statistically significant difference in a prespecified group of primary cancer types: lung, head and neck, colon/rectum, pancreas, myeloma/plasma cell neoplasm, liver/bile duct, stomach, esophagus, anus, lymphoma, ovary and bladder.
  - If a statistically significant reduction in incidence rate is found, continue by evaluating for a difference in all cancer types excluding prostate cancer.
  - If the above evaluations are both significant, evaluate for a difference in all cancer types.

Secondary objectives and endpoints also reported in Sasieni et al.^26^ include:

- For cancers that are routinely staged, demonstrate a statistically significant reduction in the incidence rate of advanced cancers (defined as a cancer diagnosed at stage III or IV or one that results in a cancer-specific death) in the intervention arm as compared to the control arm after three annual tests and an average of 16−18 months of follow-up after the third test, using a fixed-sequence strategy as below:
  - First, evaluate for a statistically significant difference in a prespecified group of primary cancer types: lung, head and neck, colon/rectum, pancreas, myeloma/plasma cell neoplasm, liver/bile duct, stomach, esophagus, anus, lymphoma, ovary and bladder.
  - If a statistically significant reduction in incidence rate is found, continue by evaluating for a difference in all cancer types excluding prostate cancer.
  - If the above evaluations are both significant, evaluate for a difference in all cancer types.
  This objective is a sensitivity analysis of the primary objective, with a broader definition of ‘advanced cancer’.
- For cancers that are routinely staged, demonstrate a difference in the proportion of stage I and II cancers between the two arms of the study in the third testing round (that is, for cancers diagnosed within 12 months of follow-up after the third test).
- Evaluate the safety, including harms, of the testing pathway in the intervention arm among individuals with positive MCED test results. (Note that the safety profile was not fully characterized in the paper, so evaluation of safety as an objective is also listed below.)
- Demonstrate a reduction in the incidence rate of stage IV cancers diagnosed in the intervention arm compared to the control arm, after three annual tests and an average of 16−18 months of follow-up after the third test:
  - For a prespecified group of cancer types: lung, head and neck, colon/rectum, pancreas, myeloma/plasma cell neoplasm, liver/bile duct, stomach, esophagus, anus, lymphoma, ovary and bladder;
  - For all cancer types excluding prostate cancer;
  - For all cancer types.
- Demonstrate a statistically significant difference in cancer detection rates in the two arms of the study with 12 months of follow-up after the first test.
- Summarize stage distribution per cancer type for the two arms and compare the incidence rate of stage IV alone as well as stage III and IV cancers, after the third screening and an average 16−18 months of follow-up.
- Demonstrate a reduction in the incidence rate of stage III and IV cancers, excluding breast, cervix and colon/rectum (that is, those without a standard-of-care screening modality) as compared to the control arm after the third annual screening and an average 16−18 months of follow-up.
- Compare the absolute numbers of stage IV cancers after the second blood draw and 12 months of follow-up, with prevalent cases excluded.
- Compare the absolute numbers of stage III and IV cancers after the third blood draw and an average 16−18 months of follow-up, with cases from the first and second screenings excluded.

Other secondary objectives and endpoints not reported here include:

- Demonstrate a statistically significant reduction in the incidence rate of stage IV cancers diagnosed in the intervention arm compared to the control arm, after the initial screening round and 12 months of follow-up (excluding cancers identified by the test performed at the 12-month visit):
  - For a prespecified group of cancer types: lung, head and neck, colon/rectum, pancreas, myeloma/plasma cell neoplasm, liver/bile duct, stomach, esophagus, anus, lymphoma, ovary and bladder;
  - For all cancer types excluding prostate cancer;
  - For all cancer types.
- Evaluate the safety, including harms, of the testing pathway in the intervention arm among individuals with positive MCED test results.
  - Assess participant-reported psychological impact, including anxiety, among those receiving a positive MCED test result after the initial blood draw at various timepoints.
  - Examine the radiation exposure by participants associated with follow-up diagnostic procedures after a positive MCED test result.
  - Describe the number and type of invasive procedures performed and number of complications and deaths associated with follow-up diagnostic procedures.
- Model the expected differences in cancer-specific mortality at 7 years after randomization based on cancers diagnosed within an average of 40−42 months of randomization.
- Compare cancer-specific mortality in the intervention and control arms after the third screening and an average 16−18 months of follow-up, for a prespecified group of cancer types: lung, head and neck, colorectal, pancreas, myeloma/plasma cell neoplasm, liver/bile duct, stomach, esophagus, anus, lymphoma, ovary and bladder.
- Compare cancer-specific mortality in the intervention and control arms for all cancer types at (1) 3 years and (2) 6 years after the last study visit.
- Assess the impact of use of the MCED test across three annual timepoints on healthcare resource utilization for cancer diagnosis and treatment.
- Assess the potential impact of overdiagnosis by comparing the cumulative incidence (Kaplan−Meier curves) of cancers diagnosed after a positive baseline MCED test up to 36−48 months after randomization between the intervention and control arms (retrospectively testing baseline samples from all participants diagnosed with cancer in the control arm).

For all objectives, follow-up for cancer diagnoses will continue for all participants until 12 months after the third test for the last participant. This will give an average of 16−18 months of follow-up after the third test or 40−42 months from randomization. Note that follow-up of cancer diagnoses for all participants will continue and will be included in the analyses until 12 months after the last participant’s third test even if some participants miss one or both of their second or third blood draws.

### MCED test performance

A detailed description of the analyses presented in this paper is available in the statistical analysis plan, previously published in the supplement of Sasieni et al.^26^.

The test performance analyzable set at each screening round comprised intervention arm participants who were clinically eligible and clinically evaluable, had at least one evaluable MCED test result for that round and had not been diagnosed with cancer during a previous round of the study (Fig. 1). In the invite letter to participants for subsequent screening rounds, those who had received cancer treatment were instructed to contact the trial nurses who advised them regarding their ongoing participation in the trial. Participants who had been diagnosed with cancer but returned for testing in the subsequent screening round were excluded from the test performance analyzable set in the subsequent screening round.

For each screening round, participants were passively followed for approximately 12 months to determine cancer status (cancer or no cancer) based on a confirmed cancer diagnosis of a hematologic malignancy (with behavior code 3 based on International Classification of Diseases for Oncology, 3rd Edition (ICD-O-3)) or an invasive solid tumor, excluding basal cell carcinoma and squamous cell carcinoma of the skin. The prespecified analysis follow-up window ended at 12 months after the participant’s blood collection, at the blood collection for the subsequent screening round or in the event of death, emigration or withdrawal from passive data follow-up, whichever occurred first.

Determinations of true-positive, false-positive, true-negative and false-negative test result status were based on whether a participant had a cancer diagnosis within the follow-up window for the given MCED testing round (definitions in Supplementary Table 15). For each screening round, performance metrics including PPV, NPV, specificity and episode sensitivity, positive likelihood ratio and negative likelihood ratio were calculated using standard definitions based on these classifications (Supplementary Table 15).

The overall accuracy of CSO prediction for each screening round was defined as the proportion of participants with true-positive test results where the CSO prediction (returned on the test report) matched the clinical CSO among all true-positive test results for that round. CSO accuracy was evaluated considering the first and second prediction together (CSO1/CSO2), the first prediction only (CSO1) or the second prediction only (CSO2) when CSO1 was incorrect. For each CSO, PPV by CSO was calculated as the proportion of participants who were diagnosed with cancer within the follow-up window with positive MCED test results and the relevant CSO prediction. Confusion matrices show only CSO1 predictions and clinical CSO among true-positive test results for that round.

Performance was summarized for each annual round and across all three rounds by summing prespecified numerators and denominators.

### Exploratory analyses of false-positive MCED test results

The MCED test was previously shown to detect recurrences^15,16^. It is, therefore, possible that a positive MCED test for participants with a prior cancer history detected a recurrent cancer. However, as the NCRAS registers only primary cancers and not recurrent cancers, these results would be classified as false positive, increasing the reported false-positive rate. To assess this potential bias, recurrences were identified through review of any pathology, radiotherapy, anticancer drug prescribing or multidisciplinary team meeting data in records submitted for all participants with a positive MCED test result and a prior cancer history by an NCRAS histopathologist engaged under contract with, but independent of, GRAIL. With these data, an exploratory PPV analysis was conducted in which MCED-positive recurrences were reclassified from false positive to true positive.

A descriptive long-term follow-up analysis was conducted among results classified as false positive in the first and second screening rounds to identify cancer diagnoses occurring after the prespecified follow-up window. This analysis was motivated by previous work suggesting higher rates of diagnosis with cancer of the initially suspected type in patients with previous urgent suspected cancer referrals that had not resulted in diagnosis^47^. For participants with a later cancer diagnosis, concordance between the initial CSO1/CSO2 and CSO1 predictions reported and the subsequent cancer diagnosis was also assessed.

### Statistics and reproducibility

NHS-Galleri was a double-blind, randomized controlled trial comparing cancer diagnoses in an intervention arm (blood tested by MCED test) to a control arm (blood stored). The sample size of the NHS-Galleri trial was based on microsimulations that indicated that approximately 140,000 participants were necessary for at least 90% power for the between-arm primary endpoint (stage III/IV reduction) after three rounds of screening, with 12 months of follow-up after the last participant’s third visit, including various natural history assumptions.

This paper reports on MCED test performance and cancer diagnoses in the test performance analyzable set, which included only intervention arm participants. The test performance analyzable set was defined as intervention arm participants who were clinically eligible and evaluable (that is, evaluable MCED test result and successfully traced in England’s population-based cancer registry (the NCRAS) to confirm cancer status). As noted above in the ‘Data collection’ section, cancer diagnosis data were passively collected even for participants who did not attend subsequent blood collections unless they withdrew from the passive follow-up, meaning that they remained included in the test analyzable set for the round for which they had an analyzable MCED test result. The number of participants who were not included in a given analysis due to missing subgroup categories are noted in the tables and figures.

As stated above in the ‘Study objectives and endpoints’ section, episode sensitivity, specificity, PPV, NPV and CSO accuracy were secondary endpoints. Estimates were calculated as proportions with two-sided 95% CIs. No formal hypothesis testing or multiplicity adjustment was applied for test performance endpoints. The two-sided 95% CIs reported here were constructed using the Wilson (score) methods^48^. Further details of estimate calculations can be found in the statistical analysis plan available in Sasieni et al.^26^. Estimates were not made for metrics if there were fewer than five cases within a subgroup, although numbers are still reported.

Clinical data were captured in the GRAIL electronic data capture system, a validated and 21 CFR Part 11-compliant system, and supplemented with data from NCRAS datasets.

Analyses were conducted in Stata (version 18)^49^ and independently validated in R (version 4.3.2)^50^.

### Reporting summary

Further information on research design is available in the Nature Portfolio Reporting Summary linked to this article.

## Online content

Any methods, additional references, Nature Portfolio reporting summaries, source data, extended data, supplementary information, acknowledgements, peer review information; details of author contributions and competing interests; and statements of data and code availability are available at https://doi.org/10.1038/s41591-026-04652-8.

## Supplementary information

Supplementary Information Tables 1−15 and Figs. 1−4.

Reporting Summary

## Data Availability

Individual-level participant cancer outcome data are not publicly available and cannot be shared by the authors due to confidentiality requirements and restrictions under the applicable data-sharing agreements with NCRAS data providers. Aggregated, anonymized data supporting the findings are provided in the paper and supplementary materials. The trial protocol and statistical analysis plan with amendments are provided as a supplement in Sasieni et al.^26^.

## References

[1] Siegel, R. L., Kratzer, T. B., Wagle, N. S., Sung, H. & Jemal, A. Cancer statistics, 2026. *CA Cancer J. Clin.* **76**, e70043 (2026). 10.3322/caac.70043 PMC12798275 41528114

[2] Goddard, K. A. B. et al. Estimation of cancer deaths averted from prevention, screening, and treatment efforts, 1975−2020. *JAMA Oncol.* **11**, 162 (2025). 10.1001/jamaoncol.2024.5381 PMC11622066 39636625

[3] Cancer Survival in England, cancers diagnosed 2015 to 2019, followed up to 2020 https://digital.nhs.uk/data-and-information/publications/statistical/cancer-survival-in-england/cancers-diagnosed-2015-to-2019-followed-up-to-2020 (NHS Digital, 2022).

[4] Routes to Diagnosis https://nhsd-ndrs.shinyapps.io/routes_to_diagnosis/ (National Disease Registration Service, 2024).

[5] Sinicrope, F. A. et al. Randomized trial of standard chemotherapy alone or combined with atezolizumab as adjuvant therapy for patients with stage III deficient DNA mismatch repair (dMMR) colon cancer (Alliance A021502; ATOMIC). *J. Clin. Oncol.* **43**, LBA1 (2025).

[6] Adkins, D. et al. Neoadjuvant and adjuvant pembrolizumab plus standard of care (SOC) in resectable locally advanced head and neck squamous cell carcinoma (LA HNSCC): exploratory efficacy analyses of the phase 3 KEYNOTE-689 study. *J. Clin. Oncol.* **43**, 6012–6012 (2025).

[7] Chi, S. A. et al. Trends in survival rates of non–small cell lung cancer with use of molecular testing and targeted therapy in Korea, 2010−2020. *JAMA Netw. Open* **6**, e232002 (2023). 10.1001/jamanetworkopen.2023.2002 PMC10020884 36929402

[8] Cao, S. et al. Recurrence and survival of patients with stage III endometrial cancer after radical surgery followed by adjuvant chemo- or chemoradiotherapy: a systematic review and meta-analysis. *BMC Cancer* **23**, 31 (2023). 10.1186/s12885-022-10482-x PMC9827697 36624407

[9] UK NSC Recommendations https://view-health-screening-recommendations.service.gov.uk/ (UK National Screening Committee, 2026).

[10] U.S. Preventive Services Task Force. A and B Recommendations https://www.uspreventiveservicestaskforce.org/uspstf/recommendation-topics/uspstf-a-and-b-recommendations (2025).

[11] NORC at the University of Chicago. Percent of Cancers Detected by Screening in the U.S. https://cancerdetection.norc.org/ (2022).

[12] Kisiel, J. B. et al. Shifting the cancer screening paradigm: developing a multi-biomarker class approach to multi-cancer early detection testing. *Life (Basel)* **14**, 925 (2024). 10.3390/life14080925 PMC11355654 39202669

[13] Brito-Rocha, T., Constâncio, V., Henrique, R. & Jerónimo, C. Shifting the cancer screening paradigm: the rising potential of blood-based multi-cancer early detection tests. *Cells* **12**, 935 (2023). 10.3390/cells12060935 PMC10047029 36980276

[14] Klein, E. A. et al. Clinical validation of a targeted methylation-based multi-cancer early detection test using an independent validation set. *Ann. Oncol.* **32**, 1167–1177 (2021). 10.1016/j.annonc.2021.05.806 34176681

[15] Schrag, D. et al. Blood-based tests for multicancer early detection (PATHFINDER): a prospective cohort study. *Lancet* **402**, 1251–1260 (2023). 10.1016/S0140-6736(23)01700-2 PMC11027492 37805216

[16] Nabavizadeh, N. et al. Performance and safety of a multi-cancer early detection test: the PATHFINDER 2 study. *Nat. Med.* 10.1038/s41591-026-04618-w (2026). PMC13668646 42773207

[17] Liu, M. C. et al. Sensitive and specific multi-cancer detection and localization using methylation signatures in cell-free DNA. *Ann. Oncol.* **31**, 745–759 (2020). 10.1016/j.annonc.2020.02.011 PMC8274402 33506766

[18] Jamshidi, A. et al. Evaluation of cell-free DNA approaches for multi-cancer early detection. *Cancer Cell* **40**, 1537–1549 (2022). 10.1016/j.ccell.2022.10.022 36400018

[19] Atwood, C. et al. REFLECTION: real-world evidence study of multi-cancer early detection (MCED) among veterans in the Veterans Affairs Healthcare System (VA) https://grail.com/wp-content/uploads/2025/10/Atwood.EDCC-2025.REFLECTION-VA.Slides_Final-1.pdf (2025).

[20] Matrana, M. et al. Real-world data and clinical experience from over 100,000 multi-cancer early detection tests. *Nat. Commun.* **16**, 9625 (2025). 10.1038/s41467-025-64094-7 PMC12578847 41173830

[21] Hurt, R. T. et al. Implementation of a multicancer detection (MCD) test in a tertiary referral center in asymptomatic patients: an 18-month prospective cohort study. *J. Prim. Care Community Health* **16**, 21501319251329290 (2025). 10.1177/21501319251329290 PMC11938867 40132152

[22] O’Donnell, E. K. et al. Diagnostic outcomes among patients with positive multi-cancer early detection test results. *Cancer Res. Commun.* **6**, 511–515 (2026). 10.1158/2767-9764.CRC-25-0723 PMC13012066 41721625

[23] Agarwal, G., Carlson, J. W. & Broyles, D. R. Implementation of a multi-cancer early detection test using a centralized model within a multi-state health system. *J. Clin. Oncol.* **41**, 1526 (2023).

[24] Sue, E. et al. Implementation of a multi-cancer early detection (MCED) test in a private practice: adoption, performance, and repeat-testing patterns. *J. Clin. Oncol.* **44**, 10532 (2026).

[25] Neal, R. D. et al. Cell-free DNA–based multi-cancer early detection test in an asymptomatic screening population (NHS-Galleri): design of a pragmatic, prospective randomised controlled trial. *Cancers* **14**, 4818 (2022). 10.3390/cancers14194818 PMC9564213 36230741

[26] Sasieni, P. et al. Impact of multi-cancer early detection test on late-stage cancer diagnosis. *N. Engl. J. Med.* (in the press).10.1056/NEJMoa250572342776074

[27] Swanton, C. et al. NHS-Galleri trial: enriched enrolment approaches and sociodemographic characteristics of enrolled participants. *Clin. Trials* **22**, 227–238 (2025). 10.1177/17407745241302477 PMC11986080 39862108

[28] Hayward, J. H., Lee, A. Y., Sickles, E. A. & Ray, K. M. Prevalent vs incident screen: why does it matter? *J. Breast Imaging* **6**, 232–237 (2024). 10.1093/jbi/wbad096 38190264

[29] Amin, M. B. et al. ed*s AJCC Cancer Staging Manual* 8th edn (Springer, 2017).

[30] Urgent suspected cancer referrals: referral, conversion and detection rates, April 2021 to March 2023 https://digital.nhs.uk/data-and-information/publications/statistical/mi-urgent-suspected-cancer-referrals-referral-conversion-and-detection-rates/april-2021-to-march-2023 (NHS Digital, 2024).

[31] Dolly, S. O. et al. The effectiveness of the Guy’s Rapid Diagnostic Clinic (RDC) in detecting cancer and serious conditions in vague symptom patients. *Br. J. Cancer* **124**, 1079–1087 (2021). 10.1038/s41416-020-01207-7 PMC7783491 33402736

[32] Raginel, T. et al. A population-based comparison of immunochemical fecal occult blood tests for colorectal cancer screening. *Gastroenterology* **144**, 918–925 (2013). 10.1053/j.gastro.2013.01.042 23376426

[33] Lee, C. I. et al. National performance benchmarks for screening digital breast tomosynthesis: update from the Breast Cancer Surveillance Consortium. *Radiology* **307**, e222499 (2023). 10.1148/radiol.222499 PMC10323294 37039687

[34] Melnikow, J. et al. Screening for cervical cancer with high-risk human papillomavirus testing: updated evidence report and systematic review for the US Preventive Services Task Force. *JAMA* **320**, 687–705 (2018). 10.1001/jama.2018.10400 PMC13387487 30140883

[35] Chung, D. C. et al. A cell-free DNA blood-based test for colorectal cancer screening. *N. Engl. J. Med.* **390**, 973–983 (2024). 10.1056/NEJMoa2304714 38477985

[36] Jackson, A. et al. Diagnostic implications of multi-cancer early detection testing in the investigation of cancer symptoms: an exploratory retrospective analysis of the SYMPLIFY study. *Lancet Reg. Health Eur.* **66**, 101720 (2026). 10.1016/j.lanepe.2026.101720 PMC13235408 42254810

[37] Frankel, S. D. et al. Initial versus subsequent screening mammography: comparison of findings and their prognostic significance. *AJR Am. J. Roentgenol.* **164**, 1107–1109 (1995). 10.2214/ajr.164.5.7717214 7717214

[38] National Lung Screening Trial Research Team. Reduced lung-cancer mortality with low-dose computed tomographic screening. *N. Engl. J. Med.* **365**, 395–409 (2011). 10.1056/NEJMoa1102873 PMC4356534 21714641

[39] de Koning, H. J. et al. Reduced lung-cancer mortality with volume CT screening in a randomized trial. *N. Engl. J. Med.* **382**, 503–513 (2020). 10.1056/NEJMoa1911793 31995683

[40] Wang, Y., Jiang, Y., Bai, Y. & Xu, H. Do cancer survivors have an increased risk of developing subsequent cancer? A population-based study. *J. Transl. Med.* **23**, 355 (2025). 10.1186/s12967-025-06379-5 PMC11927235 40119453

[41] Caturegli, G. et al. Smoking status of the US cancer population and a new perspective from the National Cancer Database. *JAMA Oncol.* **11**, 564−566 (2025). 10.1001/jamaoncol.2025.0247 PMC11950973 40146125

[42] Pinsky, P., Lange, J. & Etzioni, R. Estimating stage-specific sensitivity for cancer screening tests. *J. Med. Screen*. **30**, 69−73 (2023). 10.1177/09691413231154801 PMC10245292 36734139

[43] Alix-Panabières, C. & Pantel, K. From discovery to diagnosis: a perspective for circulating tumor cells in personalized oncology. *Cancer Discov.* **15**, 1985–2001 (2025). 10.1158/2159-8290.CD-25-0164 40825223

[44] Wan, J. C. M., Sasieni, P. & Rosenfeld, N. Promises and pitfalls of multi-cancer early detection using liquid biopsy tests. *Nat. Rev. Clin. Oncol.* **22**, 566–580 (2025). 10.1038/s41571-025-01033-x 40514453

[45] Lowenhoff, I. et al. Clinical referral to the NHS following multi-cancer early detection test results from the NHS-Galleri trial. *Front. Oncol.* **15**, 1511816 (2025). 10.3389/fonc.2025.1511816 PMC12096138 40406252

[46] Ethnic group, national identity and religion https://www.ons.gov.uk/methodology/classificationsandstandards/measuringequality/ethnicgroupnationalidentityandreligion (Office for National Statistics, 2023).

[47] Scott, S. E. et al. Future cancer risk after urgent suspected cancer referral in England when cancer is not found: a national cohort study. *Lancet Oncol.* **24**, 1242–1251 (2023). 10.1016/S1470-2045(23)00435-7 37922929

[48] Wilson, E. Probable inference, the law of succession, and statistical inference. *J. Am. Stat. Assoc.* **22**, 209–212 (1927).

[49] StataCorp. *Stata Statistical Software: Release 19* (StataCorp LLC, 2023).

[50] R Core Team *R: A Language and Environment for Statistical Computing* (R Core Team, 2023).

