# Supplementary material for: Performance of a multi-cancer early detection test in the randomized controlled NHS-Galleri trial
Source: Nat Med. 2026 Sep 22;32(10):3770–80. doi: 10.1038/s41591-026-04652-8 (PMC13668664; doi:10.1038/s41591-026-04652-8)
Supplement: Supplementary file 1 [file 41591_2026_4652_MOESM1_ESM.pdf]

nature medicine

Supplementary information https://doi.org/10.1038/s41591-026-04652-8

Performance of a multi-cancer early detection test in the randomized controlled NHS-Galleri trial

In the format provided by the authors and unedited

Supplementary Information

Table of Contents

Tables **2**

Supplementary Table S1. 12-Month Episode Sensitivity Metrics for the Subgroup of 12 Prespecified Cancer Types and Individual Cancer Types by Round and in Aggregate. 2

Supplementary Table S2. Performance Metrics by Subgroup: Age at Baseline........ 10

Supplementary Table S3. Performance Metrics by Subgroup: Sex. 13

Supplementary Table S4. Performance Metrics by Subgroup: Smoking Status........ 15

Supplementary Table S5. Performance Metrics by Subgroup: Prior Cancer History. 18

Supplementary Table S6. Performance Metrics by Subgroup: Race/Ethnicity........ 20

Supplementary Table S7. Performance Metrics by Subgroup: Index of Multiple Deprivation. 24

Supplementary Table S8. Performance Metrics by Subgroup: Education........ 28

Supplementary Table S9. PPV by CSO Prediction by Round and in Aggregate........ 33

Supplementary Table S10. Characteristics of First Primary Cancers in Participants. 39

Supplementary Table S11. Distribution of MCED-Detected Cancers by AJCC Cancer Types........ 52

Supplementary Table S12. Distribution of Stage for Cancers per First-Cancer Diagnosed. 55

Supplementary Table S13. Sensitivity Analysis for PPV by Screening Round and in Aggregate Accounting for Additionally Identified Recurrent Cancers........ 57

Supplementary Table S14. Exploratory Analysis for Participants With False-positive Results with Additional Follow-up. 58

Supplementary Table S15. Definitions of Screening Performance Metrics........ 59

Figures **61**

Supplementary Figure S1. Aggregate Episode Sensitivity by Cancer Type Diagnosed. 61

Supplementary Figure S2. Confusion Matrix of Predicted CSO vs Clinical CSO by Sex by Round and in Aggregate. 63

Supplementary Figure S3. Aggregate PPV by Predicted CSO. 68

Supplementary Figure S4. Stage and Type of Cancer Diagnosed Among Participants With True-Positive and False-Negative MCED Test Results. 70

Tables

Supplementary Table S1. 12-Month Episode Sensitivity Metrics for the Subgroup of 12 Prespecified Cancer Types and Individual Cancer Types by Round and in Aggregate.

	Participants with a cancer diagnosis		
	**Positive MCED test result,** n (%)	**Negative MCED test result,** n (%)	**Episode sensitivity,**[a] % (n/N) (95% CI)
**Subgroup of 12 prespecified cancer types**[b,c]			
First screening round	288 (68.7)	166 (23.4)	63.4 (288/454) (58.9, 67.7)
Second screening round	171 (65.5)	188 (26.8)	47.6 (171/359) (42.5, 52.8)
Third screening round	187 (72.8)	182 (25.9)	50.7 (187/369) (45.6, 55.7)
Aggregate	646 (68.9)	536 (25.4)	54.7 (646/1182) (51.8, 57.5)
**Cancer type diagnosed**			
**First screening round (n=1127)**			
Total, n	419	708	
Anus	3 (0.7)	2 (0.3)	60.0 (3/5) (23.1, 88.2)
Bladder	1 (0.2)	14 (2.0)	6.7 (1/15) (1.2, 29.8)
Breast	26 (6.2)	118 (16.7)	18.1 (26/144) (12.6, 25.1)
Cervix	1 (0.2)	1 (0.1)	1/2
Colon/Rectum	83 (19.8)	42 (5.9)	66.4 (83/125) (57.7, 74.1)
Gallbladder	5 (1.2)	4 (0.6)	55.6 (5/9) (26.7, 81.1)
Head and Neck	14 (3.3)	11 (1.6)	56.0 (14/25) (37.1, 73.3)
Kidney	8 (1.9)	22 (3.1)	26.7 (8/30) (14.2, 44.4)

	Participants with a cancer diagnosis		
	**Positive MCED test result,** n (%)	**Negative MCED test result,** n (%)	**Episode sensitivity,**[a] % (n/N) (95% CI)
Liver/Bile-duct	12 (2.9)	3 (0.4)	80.0 (12/15) (54.8, 93.0)
Lung	49 (11.7)	28 (4.0)	63.6 (49/77) (52.5, 73.5)
Lymphoid Leukaemia	13 (3.1)	14 (2.0)	48.1 (13/27) (30.7, 66.0)
Lymphoma	33 (7.9)	20 (2.8)	62.3 (33/53) (48.8, 74.1)
Melanoma	2 (0.5)	49 (6.9)	3.9 (2/51) (1.1, 13.2)
Myeloid Neoplasm	1 (0.2)	17 (2.4)	5.6 (1/18) (1.0, 25.8)
Oesophagus	20 (4.8)	7 (1.0)	74.1 (20/27) (55.3, 86.8)
Ovary	18 (4.3)	8 (1.1)	69.2 (18/26) (50.0, 83.5)
Pancreas	18 (4.3)	15 (2.1)	54.5 (18/33) (38.0, 70.2)
Plasma Cell Neoplasm	21 (5.0)	10 (1.4)	67.7 (21/31) (50.1, 81.4)
Prostate	48 (11.5)	236 (33.3)	16.9 (48/284) (13.0, 21.7)
Sarcoma	2 (0.5)	7 (1.0)	22.2 (2/9) (6.3, 54.7)
Stomach	9 (2.1)	5 (0.7)	64.3 (9/14) (38.8, 83.7)
Thyroid	1 (0.2)	4 (0.6)	20.0 (1/5) (3.6, 62.4)
Urothelial Tract	0	2 (0.3)	0/2
Uterus	7 (1.7)	25 (3.5)	21.9 (7/32) (11.0, 38.8)
Other[d]	3 (0.7)	30 (4.2)	9.1 (3/33) (3.1, 23.6)

	Participants with a cancer diagnosis		
	**Positive MCED test result,** n (%)	**Negative MCED test result,** n (%)	**Episode sensitivity,**[a] % (n/N) (95% CI)
Multiple Cancers	19 (4.5)	12 (1.7)	61.3 (19/31) (43.8, 76.3)
NCRAS Cancer of Unknown Primary[e]	2 (0.5)	2 (0.3)	2/4
**Second screening round (n=969)**			
Total, n	261	702	
Anus	3 (1.1)	1 (0.1)	3/4
Bladder	3 (1.1)	14 (2.0)	17.6 (3/17) (6.2, 41.0)
Breast	15 (5.7)	92 (13.1)	14.0 (15/107) (8.7, 21.8)
Cervix	1 (0.4)	1 (0.1)	1/2
Colon/Rectum	54 (20.7)	49 (7.0)	52.4 (54/103) (42.9, 61.8)
Gallbladder	2 (0.8)	1 (0.1)	2/3
Head and Neck	7 (2.7)	15 (2.1)	31.8 (7/22) (16.4, 52.7)
Kidney	1 (0.4)	19 (2.7)	5.0 (1/20) (0.9, 23.6)
Liver/Bile-duct	7 (2.7)	1 (0.1)	87.5 (7/8) (52.9, 97.8)
Lung	36 (13.8)	53 (7.5)	40.4 (36/89) (30.9, 50.8)
Lymphoid Leukaemia	7 (2.7)	13 (1.9)	35.0 (7/20) (18.1, 56.7)
Lymphoma	19 (7.3)	20 (2.8)	48.7 (19/39) (33.9, 63.8)
Melanoma	1 (0.4)	47 (6.7)	2.1 (1/48) (0.4, 10.9)
Myeloid Neoplasm	0	16 (2.3)	0.0 (0/16) (0.0, 19.4)

	Participants with a cancer diagnosis		
	**Positive MCED test result,** n (%)	**Negative MCED test result,** n (%)	**Episode sensitivity,**[a] % (n/N) (95% CI)
Oesophagus	10 (3.8)	4 (0.6)	71.4 (10/14) (45.4, 88.3)
Ovary	6 (2.3)	7 (1.0)	46.2 (6/13) (23.2, 70.9)
Pancreas	10 (3.8)	10 (1.4)	50.0 (10/20) (29.9, 70.1)
Plasma Cell Neoplasm	9 (3.4)	8 (1.1)	52.9 (9/17) (31.0, 73.8)
Prostate	33 (12.6)	242 (34.5)	12.0 (33/275) (8.7, 16.4)
Sarcoma	1 (0.4)	4 (0.6)	20.0 (1/5) (3.6, 62.4)
Stomach	6 (2.3)	4 (0.6)	60.0 (6/10) (31.3, 83.2)
Thyroid	0	7 (1.0)	0.0 (0/7) (0.0, 35.4)
Urothelial Tract	3 (1.1)	6 (0.9)	33.3 (3/9) (12.1, 64.6)
Uterus	6 (2.3)	20 (2.8)	23.1 (6/26) (11.0, 42.1)
Other[f]	8 (3.1)	30 (4.3)	21.1 (8/38) (11.1, 36.3)
Multiple Cancers	11 (4.2)	15 (2.1)	42.3 (11/26) (25.5, 61.1)
NCRAS Cancer of Unknown Primary[e]	2 (0.8)	3 (0.4)	40.0 (2/5) (11.8, 76.9)
**Third screening round (n=961)**			
Total, n	257	704	
Anus	1 (0.4)	0	1/1
Bladder	4 (1.6)	14 (2.0)	22.2 (4/18) (9.0, 45.2)
Breast	15 (5.8)	91 (12.9)	14.2 (15/106)

	Participants with a cancer diagnosis		
	**Positive MCED test result,** n (%)	**Negative MCED test result,** n (%)	**Episode sensitivity,**[a] % (n/N) (95% CI)
			(8.8, 22.0)
Cervix	0	1 (0.1)	0/1
Colon/Rectum	49 (19.1)	39 (5.5)	55.7 (49/88) (45.3, 65.6)
Gallbladder	7 (2.7)	3 (0.4)	70.0 (7/10) (39.7, 89.2)
Head and Neck	11 (4.3)	11 (1.6)	50.0 (11/22) (30.7, 69.3)
Kidney	4 (1.6)	15 (2.1)	21.1 (4/19) (8.5, 43.3)
Liver/Bile-duct	13 (5.1)	0	100 (13/13) (77.2, 100)
Lung	42 (16.3)	45 (6.4)	48.3 (42/87) (38.1, 58.6)
Lymphoid Leukaemia	5 (1.9)	5 (0.7)	50.0 (5/10) (23.7, 76.3)
Lymphoma	19 (7.4)	23 (3.3)	45.2 (19/42) (31.2, 60.1)
Melanoma	1 (0.4)	57 (8.1)	1.7 (1/58) (0.3, 9.1)
Myeloid Neoplasm	1 (0.4)	26 (3.7)	3.7 (1/27) (0.7, 18.3)
Oesophagus	12 (4.7)	10 (1.4)	54.5 (12/22) (34.7, 73.1)
Ovary	10 (3.9)	7 (1.0)	58.8 (10/17) (36.0, 78.4)
Pancreas	8 (3.1)	17 (2.4)	32.0 (8/25) (17.2, 51.6)
Plasma Cell Neoplasm	13 (5.1)	11 (1.6)	54.2 (13/24) (35.1, 72.1)
Prostate	22 (8.6)	235 (33.4)	8.6 (22/257) (5.7, 12.6)

	Participants with a cancer diagnosis		
	**Positive MCED test result,** n (%)	**Negative MCED test result,** n (%)	**Episode sensitivity,**[a] % (n/N) (95% CI)
Sarcoma	0	10 (1.4)	0.0 (0/10)<br>(0.0, 27.8)
Stomach	4 (1.6)	1 (0.1)	80.0 (4/5)<br>(37.6, 96.4)
Thyroid	0	8 (1.1)	0.0 (0/8)<br>(0.0, 32.4)
Urothelial Tract	0	4 (0.6)	0/4
Uterus	4 (1.6)	17 (2.4)	19.0 (4/21)<br>(7.7, 40.0)
Other[g]	3 (1.2)	32 (4.5)	8.6 (3/35)<br>(3.0, 22.4)
Multiple Cancers	8 (3.1)	20 (2.8)	28.6 (8/28)<br>(15.3, 47.1)
NCRAS Cancer of Unknown Primary[e]	1 (0.4)	2 (0.3)	1/3
**Aggregate (n=3051)**			
Total, n	937	2114	
Anus	7 (0.7)	3 (0.1)	70.0 (7/10)<br>(39.7, 89.2)
Bladder	8 (0.9)	42 (2.0)	16.0 (8/50)<br>(8.3, 28.5)
Breast	56 (6.0)	301 (14.2)	15.7 (56/357)<br>(12.3, 19.8)
Cervix	2 (0.2)	3 (0.1)	40.0 (2/5)<br>(11.8, 76.9)
Colon/Rectum	186 (19.9)	130 (6.1)	58.9 (186/316)<br>(53.4, 64.1)
Gallbladder	14 (1.5)	8 (0.4)	63.6 (14/22)<br>(43.0, 80.3)
Head and Neck	32 (3.4)	37 (1.8)	46.4 (32/69)<br>(35.1, 58.0)
Kidney	13 (1.4)	56 (2.6)	18.8 (13/69)

	Participants with a cancer diagnosis		
	**Positive MCED test result,** n (%)	**Negative MCED test result,** n (%)	**Episode sensitivity,**[a] % (n/N) (95% CI)
			(11.4, 29.6)
Liver/Bile-duct	32 (3.4)	4 (0.2)	88.9 (32/36) (74.7, 95.6)
Lung	127 (13.6)	126 (6.0)	50.2 (127/253) (44.1, 56.3)
Lymphoid Leukaemia	25 (2.7)	32 (1.5)	43.9 (25/57) (31.8, 56.7)
Lymphoma	71 (7.6)	63 (3.0)	53.0 (71/134) (44.6, 61.2)
Melanoma	4 (0.4)	153 (7.2)	2.5 (4/157) (1.0, 6.4)
Myeloid Neoplasm	2 (0.2)	59 (2.8)	3.3 (2/61) (0.9, 11.2)
Oesophagus	42 (4.5)	21 (1.0)	66.7 (42/63) (54.4, 77.1)
Ovary	34 (3.6)	22 (1.0)	60.7 (34/56) (47.6, 72.4)
Pancreas	36 (3.8)	42 (2.0)	46.2 (36/78) (35.5, 57.1)
Plasma Cell Neoplasm	43 (4.6)	29 (1.4)	59.7 (43/72) (48.2, 70.3)
Prostate	103 (11.0)	713 (33.7)	12.6 (103/816) (10.5, 15.1)
Sarcoma	3 (0.3)	21 (1.0)	12.5 (3/24) (4.3, 31.0)
Stomach	19 (2.0)	10 (0.5)	65.5 (19/29) (47.3, 80.1)
Thyroid	1 (0.1)	19 (0.9)	5.0 (1/20) (0.9, 23.6)
Urothelial Tract	3 (0.3)	12 (0.6)	20.0 (3/15) (7.0, 45.2)
Uterus	17 (1.8)	62 (2.9)	21.5 (17/79) (13.9, 31.8)

	Participants with a cancer diagnosis		
	**Positive MCED test result,** n (%)	**Negative MCED test result,** n (%)	**Episode sensitivity,**[a] % (n/N) (95% CI)
Other[h]	14 (1.5)	92 (4.4)	13.2 (14/106) (8.0, 21.0)
Multiple Cancers	38 (4.1)	47 (2.2)	44.7 (38/85) (34.6, 55.3)
NCRAS Cancer of Unknown Primary[e]	5 (0.5)	7 (0.3)	41.7 (5/12) (19.3, 68.0)

Participants with multiple cancer diagnoses reported were categorized as “Multiple Cancers.”

[a]Episode sensitivity is not estimated for cancer types with less than 5 cases.

[b]Participants with multiple cancers are included in the subgroup of prespecified cancers only if all cancers belong to the subgroup.

[c]Anus, bladder, colorectal, head and neck, liver/bile-duct, lung, lymphoma, oesophagus, ovary, pancreas, plasma cell neoplasm (myeloma), stomach.

[d]Other cancer types include brain (n=13), mesothelioma (n=7), small intestine (n=4), vulva (n=4), ampulla of Vater (n=1), eyelid (n=1), penis (n=1), thymus (n=1), trachea (n=1).

[e]NCRAS Cancer of Unknown Primary is defined as an invasive cancer with ICD-O-3 behaviour code 6 or 9; or an invasive cancer with behaviour code 3, topographical code C809 and unmapped cancer label.

[f]Other cancer types include brain (n=11), mesothelioma (n=9), small intestine (n=6), germ cell (n=3), vulva (n=3), thymus (n=2), carotid body (n=1), penis (n=1), skin cancer (n=1), vagina (n=1).

[g]Other cancer types include brain (n=8), mesothelioma (n=8), small intestine (n=6), skin cancer (n=3), vulva (n=3), adrenal (n=2), ampulla of Vater (n=2), penis (n=2), vagina (n=1).

[h]Other cancer types include brain (n=32), mesothelioma (n=24), small intestine (n=16), vulva (n=10), penis (n=4), skin cancer (n=4), ampulla of Vater (n=3), germ cell (n=3), thymus (n=3), adrenal (n=2), vagina (n=2), carotid body (n=1), eyelid (n=1), trachea (n=1).

MCED, multi-cancer early detection; NCRAS, National Cancer Registration and Analysis Service.

Supplementary Table S2. Performance Metrics by Subgroup: Age at Baseline.

**Screening round**	**Age 50–59y**<br>Estimate (n/N)<br>(95% CI)	**Age 60–69y**<br>Estimate (n/N)<br>(95% CI)	**Age 70–79y**<br>Estimate (n/N)<br>(95% CI)
**PPV**			
First	57.9 (44/76)<br>(46.7, 68.4)	58.3 (141/242)<br>(52.0, 64.3)	57.9 (234/404)<br>(53.1, 62.6)
Second	47.9 (23/48)<br>(34.5, 61.7)	52.8 (95/180)<br>(45.5, 59.9)	49.3 (143/290)<br>(43.6, 55.0)
Third	40.0 (24/60)<br>(28.6, 52.6)	51.3 (100/195)<br>(44.3, 58.2)	43.5 (133/306)<br>(38.0, 49.1)
Aggregate	49.5 (91/184)<br>(42.3, 56.6)	54.5 (336/617)<br>(50.5, 58.3)	51 (510/1,000)<br>(47.9, 54.1)
**NPV**			
First	99.46 (18,416/18,516)<br>(99.34, 99.56)	99.00 (27,723/28,004)<br>(98.87, 99.11)	98.58 (22,756/23,083)<br>(98.42, 98.73)
Second	99.46 (16,472/16,562)<br>(99.33, 99.56)	98.91 (25,788/26,071)<br>(98.78, 99.03)	98.46 (21,018/21,347)<br>(98.28, 98.62)
Third	99.40 (16,016/16,113)<br>(99.27, 99.51)	98.87 (25,009/25,295)<br>(98.73, 98.99)	98.42 (20,033/20,354)<br>(98.24, 98.59)
Aggregate	99.44 (50,904/51,191)<br>(99.37, 99.50)	98.93 (78,520/79,370)<br>(98.86, 99.00)	98.49 (63,807/64,784)<br>(98.4, 98.58)
**Episode sensitivity**			
First	30.6 (44/144)<br>(23.6, 38.5)	33.4 (141/422)<br>(29.1, 38.0)	41.7 (234/561)<br>(37.7, 45.8)
Second	20.4 (23/113)<br>(14.0, 28.7)	25.1 (95/378)<br>(21.0, 29.7)	30.3 (143/472)<br>(26.3, 34.6)
Third	19.8 (24/121)<br>(13.7, 27.8)	25.9 (100/386)<br>(21.8, 30.5)	29.3 (133/454)<br>(25.3, 33.6)
Aggregate	24.1 (91/378)<br>(20.0, 28.6)	28.3 (336/1186)<br>(25.8, 31.0)	34.3 (510/1487)<br>(31.9, 36.7)
**Specificity**			
First	99.83 (18,416/18,448)<br>(99.76, 99.88)	99.64 (27,723/27,824)<br>(99.56, 99.70)	99.26 (22,756/22,926)<br>(99.14, 99.36)

**Screening round**	**Age 50–59y** Estimate (n/N) (95% CI)	**Age 60–69y** Estimate (n/N) (95% CI)	**Age 70–79y** Estimate (n/N) (95% CI)
Second	99.85 (16,472/16,497) (99.78, 99.90)	99.67 (25,788/25,873) (99.59, 99.73)	99.31 (21,018/21,165) (99.18, 99.41)
Third	99.78 (16,016/16,052) (99.69, 99.84)	99.62 (25,009/25,104) (99.54, 99.69)	99.14 (20,033/20,206) (99.01, 99.26)
Aggregate	99.82 (50,904/50,997) (99.78, 99.85)	99.64 (78,520/78,801) (99.60, 99.68)	99.24 (63,807/64,297) (99.17, 99.30)
**False-positive rate**			
First	0.17 (32/18,448) (0.12, 0.24)	0.36 (101/27,824) (0.30, 0.44)	0.74 (170/22,926) (0.64, 0.86)
Second	0.15 (25/16,497) (0.10, 0.22)	0.33 (85/25,873) (0.27, 0.41)	0.69 (147/21,165) (0.59, 0.82)
Third	0.22 (36/16,052) (0.16, 0.31)	0.38 (95/25,104) (0.31, 0.46)	0.86 (173/2,0206) (0.74, 0.99)
Aggregate	0.18 (93/50,997) (0.15, 0.22)	0.36 (281/78,801) (0.32, 0.40)	0.76 (490/64,297) (0.70, 0.83)
**MCED cancer detection rate**			
First	0.24 (44/18,592) (0.18, 0.32)	0.5 (141/28,246) (0.42, 0.59)	1.00 (234/23,487) (0.88, 1.13)
Second	0.14 (23/16,610) (0.09, 0.21)	0.36 (95/26,251) (0.30, 0.44)	0.66 (143/21,637) (0.56, 0.78)
Third	0.15 (24/16,173) (0.10, 0.22)	0.39 (100/25,490) (0.32, 0.48)	0.64 (133/20,660) (0.54, 0.76)
Aggregate	0.18 (91/51,375) (0.14, 0.22)	0.42 (336/79,987) (0.38, 0.47)	0.78 (510/65,784) (0.71, 0.85)
**MCED cancer signal detection rate**			
First	0.41 (76/18,592) (0.33, 0.51)	0.86 (242/28,246) (0.76, 0.97)	1.72 (404/23,487) (1.56, 1.89)
Second	0.29 (48/16,610) (0.22, 0.38)	0.69 (180/26,251) (0.59, 0.79)	1.34 (290/21,637) (1.20, 1.50)
Third	0.37 (60/16,173) (0.29, 0.48)	0.77 (195/25,490) (0.67, 0.88)	1.48 (306/20,660) (1.33, 1.66)
Aggregate	0.36 (184/51,375)	0.77 (617/79,987)	1.52 (1000/65,784)

**Screening round**	**Age 50–59y** Estimate (n/N) (95% CI)	**Age 60–69y** Estimate (n/N) (95% CI)	**Age 70–79y** Estimate (n/N) (95% CI)
	(0.31, 0.41)	(0.71, 0.83)	(1.43, 1.62)

MCED, multi-cancer early detection; NPV, negative predictive value; PPV, positive predictive value.

Supplementary Table S3. Performance Metrics by Subgroup: Sex.

Screening round	Female<br>Estimate (n/N)<br>(95% CI)	Male<br>Estimate (n/N)<br>(95% CI)
**PPV**		
First	56.1 (162/289) (50.3, 61.7)	59.4 (257/433) (54.7, 63.9)
Second	51.7 (107/207) (44.9, 58.4)	49.5 (154/311) (44.0, 55.0)
Third	48.2 (105/218) (41.6, 54.8)	44.3 (152/343) (39.1, 49.6)
Aggregate	52.4 (374/714) (48.7, 56.0)	51.8 (563/1087) (48.8, 54.8)
**NPV**		
First	99.16 (34,749/35,043)<br>(99.06, 99.25)	98.80 (34,146/34,560)<br>(98.68, 98.91)
Second	99.18 (32,087/32,351)<br>(99.08, 99.28)	98.62 (31,191/31,629)<br>(98.48, 98.74)
Third	99.18 (31,076/31,333)<br>(99.07, 99.27)	98.53 (29,982/30,429)<br>(98.39, 98.66)
Aggregate	99.17 (97,912/98,727)<br>(99.12, 99.23)	98.66 (95,319/96,618)<br>(98.58, 98.73)
**Episode sensitivity**		
First	35.5 (162/456) (31.3, 40.0)	38.3 (257/671) (34.7, 42.0)
Second	28.8 (107/371) (24.5, 33.6)	26.0 (154/592) (22.6, 29.7)
Third	29.0 (105/362) (24.6, 33.9)	25.4 (152/599) (22.1, 29.0)
Aggregate	31.5 (374/1189) (28.9, 34.2)	30.2 (563/1862) (28.2, 32.4)
**Specificity**		
First	99.64 (34,749/34,876)<br>(99.57, 99.69)	99.49 (34,146/34,322)<br>(99.41, 99.56)
Second	99.69 (32,087/32,187)<br>(99.62, 99.74)	99.50 (31,191/31,348)<br>(99.41, 99.57)
Third	99.64 (31,076/31,189)<br>(99.56, 99.70)	99.37 (29,982/30,173)<br>(99.27, 99.45)
Aggregate	99.65 (97,912/98,252)<br>(99.62, 99.69)	99.45 (95,319/95,843)<br>(99.40, 99.50)

**Screening round**	**Female**<br>Estimate (n/N)<br>(95% CI)	**Male**<br>Estimate (n/N)<br>(95% CI)
**False-positive rate**		
First	0.36 (127/34,876) (0.31, 0.43)	0.51 (176/34,322) (0.44, 0.59)
Second	0.31 (100/32,187) (0.26, 0.38)	0.50 (157/31,348) (0.43, 0.59)
Third	0.36 (113/31,189) (0.30, 0.44)	0.63 (191/30,173) (0.55, 0.73)
Aggregate	0.35 (340/98,252) (0.31, 0.38)	0.55 (524/95,843) (0.50, 0.60)
**MCED cancer detection rate**		
First	0.46 (162/35,332) (0.39, 0.53)	0.73 (257/34,993) (0.65, 0.83)
Second	0.33 (107/32,558) (0.27, 0.40)	0.48 (154/31,940) (0.41, 0.56)
Third	0.33 (105/31,551) (0.28, 0.40)	0.49 (152/30,772) (0.42, 0.58)
Aggregate	0.38 (374/99,441) (0.34, 0.42)	0.58 (563/97,705) (0.53, 0.63)
**MCED cancer signal detection rate**		
First	0.82 (289/35,332) (0.73, 0.92)	1.24 (433/34,993) (1.13, 1.36)
Second	0.64 (207/32,558) (0.56, 0.73)	0.97 (311/31,940) (0.87, 1.09)
Third	0.69 (218/31,551) (0.61, 0.79)	1.11 (343/30,772) (1.00, 1.24)
Aggregate	0.72 (714/99,441) (0.67, 0.77)	1.11 (1087/97,705) (1.05, 1.18)

MCED, multi-cancer early detection; NPV, negative predictive value; PPV, positive predictive value.

Supplementary Table S4. Performance Metrics by Subgroup: Smoking Status.

**Screening round**	**Currently smokes** Estimate (n/N) (95% CI)	**Formerly smoked** Estimate (n/N) (95% CI)	**Never smoked** Estimate (n/N) (95% CI)
**PPV**			
First[a]	59.4 (38/64) (47.1, 70.5)	56.7 (174/307) (51.1, 62.1)	59 (207/351) (53.8, 64.0)
Second[b]	60.8 (31/51) (47.1, 73.0)	50.2 (118/235) (43.9, 56.6)	48.3 (112/232) (41.9, 54.7)
Third[c]	59.2 (29/49) (45.2, 71.8)	51.8 (133/257) (45.7, 57.8)	37.4 (95/254) (31.7, 43.5)
Aggregate[d]	59.8 (98/164) (52.1, 67.0)	53.2 (425/799) (49.7, 56.6)	49.5 (414/837) (46.1, 52.8)
**NPV**			
First[a]	98.96 (4568/4616) (98.62, 99.21)	98.90 (26,384/26,677) (98.77, 99.02)	99.04 (37,856/38,223) (98.94, 99.13)
Second[b]	98.82 (3846/3892) (98.43, 99.11)	98.72 (24,180/24,493) (98.57, 98.86)	99.05 (35,165/35,504) (98.94, 99.14)
Third[c]	98.76 (3596/3641) (98.35, 99.08)	98.67 (23,209/23,521) (98.52, 98.81)	99.00 (34,170/34,515) (98.89, 99.10)
Aggregate[d]	98.86 (12,010/12,149) (98.65, 99.03)	98.77 (73,773/74,691) (98.69, 98.85)	99.03 (107,191/108,242) (98.97, 99.09)
**Episode sensitivity**			
First[a]	44.2 (38/86) (34.2, 54.7)	37.3 (174/467) (33.0, 41.7)	36.1 (207/574) (32.2, 40.1)
Second[b]	40.3 (31/77) (30.0, 51.4)	27.4 (118/431) (23.4, 31.8)	24.8 (112/451) (21.1, 29.0)
Third[c]	39.2 (29/74) (28.9, 50.6)	29.9 (133/445) (25.8, 34.3)	21.6 (95/440) (18.0, 25.7)
Aggregate[d]	41.4 (98/237) (35.3, 47.7)	31.6 (425/1343) (29.2, 34.2)	28.3 (414/1465) (26.0, 30.6)
**Specificity**			

Screening round	**Currently smokes** Estimate (n/N) (95% CI)	**Formerly smoked** Estimate (n/N) (95% CI)	**Never smoked** Estimate (n/N) (95% CI)
First[a]	99.43 (4568/4594) (99.17, 99.61)	99.50 (26,384/26,517) (99.41, 99.58)	99.62 (37,856/38,000) (99.55, 99.68)
Second[b]	99.48 (3846/3866) (99.20, 99.66)	99.52 (24,180/24,297) (99.42, 99.60)	99.66 (35,165/35,285) (99.59, 99.72)
Third[c]	99.45 (3596/3616) (99.15, 99.64)	99.47 (23,209/23,333) (99.37, 99.55)	99.54 (34,170/34,329) (99.46, 99.60)
Aggregate[d]	99.45 (12,010/12,076) (99.31, 99.57)	99.50 (73,773/74,147) (99.44, 99.54)	99.61 (107,191/107,614) (99.57, 99.64)
**False-positive rate**			
First[a]	0.57 (26/4594) (0.39, 0.83)	0.50 (133/26,517) (0.42, 0.59)	0.38 (144/38,000) (0.32, 0.45)
Second[b]	0.52 (20/3866) (0.34, 0.80)	0.48 (117/24,297) (0.40, 0.58)	0.34 (120/35,285) (0.28, 0.41)
Third[c]	0.55 (20/3616) (0.36, 0.85)	0.53 (124/23,333) (0.45, 0.63)	0.46 (159/34,329) (0.40, 0.54)
Aggregate[d]	0.55 (66/12,076) (0.43, 0.69)	0.50 (374/74,147) (0.46, 0.56)	0.39 (423/107,614) (0.36, 0.43)
**MCED cancer detection rate**			
First[a]	0.81 (38/4,680) (0.59, 1.11)	0.64 (174/26,984) (0.56, 0.75)	0.54 (207/38,574) (0.47, 0.61)
Second[b]	0.79 (31/3,943) (0.55, 1.11)	0.48 (118/24,728) (0.40, 0.57)	0.31 (112/35,736) (0.26, 0.38)
Third[c]	0.79 (29/3,690) (0.55, 1.13)	0.56 (133/23,778) (0.47, 0.66)	0.27 (95/34,769) (0.22, 0.33)
Aggregate[d]	0.80 (98/12,313) (0.65, 0.97)	0.56 (425/75,490) (0.51, 0.62)	0.38 (414/109,079) (0.34, 0.42)
**MCED cancer signal detection rate**			
First[a]	1.37 (64/4,680) (1.07, 1.74)	1.14 (307/26,984) (1.02, 1.27)	0.91 (351/38,574) (0.82, 1.01)

**Screening round**	**Currently smokes** Estimate (n/N) (95% CI)	**Formerly smoked** Estimate (n/N) (95% CI)	**Never smoked** Estimate (n/N) (95% CI)
Second[b]	1.29 (51/3,943) (0.99, 1.70)	0.95 (235/24,728) (0.84, 1.08)	0.65 (232/35,736) (0.57, 0.74)
Third[c]	1.33 (49/3,690) (1.01, 1.75)	1.08 (257/23,778) (0.96, 1.22)	0.73 (254/34,769) (0.65, 0.83)
Aggregate[d]	1.33 (164/12,313) (1.14, 1.55)	1.06 (799/75,490) (0.99, 1.13)	0.77 (837/109,079) (0.72, 0.82)

[a]87 participants with missing subgroup values are not included.

[b]91 participants with missing subgroup values are not included.

[c]86 participants with missing subgroup values are not included.

[d]Screening episodes from participants with missing subgroup values are not included.

MCED, multi-cancer early detection; NPV, negative predictive value; PPV, positive predictive value.

Supplementary Table S5. Performance Metrics by Subgroup: Prior Cancer History.

Screening round	Prior cancer history	
	**Yes** Estimate (n/N) (95% CI)	**No** Estimate (n/N) (95% CI)
**PPV**		
First	36.5 (35/96) (27.5, 46.4)	61.3 (384/626) (57.5, 65.1)
Second	43.2 (32/74) (32.6, 54.6)	51.6 (229/444) (46.9, 56.2)
Third	22.6 (14/62) (14.0, 34.4)	48.7 (243/499) (44.3, 53.1)
Aggregate	34.9 (81/232) (29.1, 41.2)	54.6 (856/1569) (52.1, 57.0)
**NPV**		
First	98.89 (5076/5133) (98.56, 99.14)	98.99 (63,819/64,470) (98.91, 99.06)
Second	98.80 (4616/4672) (98.45, 99.08)	98.91 (58,662/59,308) (98.82, 98.99)
Third	98.45 (4392/4461) (98.05, 98.78)	98.89 (56,666/57,301) (98.80, 98.97)
Aggregate	98.72 (14,084/14,266) (98.53, 98.90)	98.93 (179,147/181,079) (98.88, 98.98)
**Episode sensitivity**		
First	38.0 (35/92) (28.8, 48.3)	37.1 (384/1035) (34.2, 40.1)
Second	36.4 (32/88) (27.1, 46.8)	26.2 (229/875) (23.4, 29.2)
Third	16.9 (14/83) (10.3, 26.3)	27.7 (243/878) (24.8, 30.7)
Aggregate	30.8 (81/263) (25.5, 36.6)	30.7 (856/2788) (29.0, 32.4)
**Specificity**		
First	98.81 (5076/5137) (98.48, 99.07)	99.62 (63,819/64,061) (99.57, 99.67)
Second	99.10 (4616/4658) (98.78, 99.33)	99.63 (58,662/58,877) (99.58, 99.68)
Third	98.92 (4392/4440) (98.57, 99.18)	99.55 (56,666/56,922) (99.49, 99.60)

Screening round	Prior cancer history	
	**Yes**<br>Estimate (n/N)<br>(95% CI)	**No**<br>Estimate (n/N)<br>(95% CI)
Aggregate	98.94 (14,084/14,235)<br>(98.76, 99.09)	99.60 (179,147/179,860)<br>(99.57, 99.63)
**False-positive rate**		
First	1.19 (61/5137) (0.93, 1.52)	0.38 (242/64,061) (0.33, 0.43)
Second	0.90 (42/4658) (0.67, 1.22)	0.37 (215/58,877) (0.32, 0.42)
Third	1.08 (48/4440) (0.82, 1.43)	0.45 (256/56,922) (0.40, 0.51)
Aggregate	1.06 (151/14,235) (0.91, 1.24)	0.40 (713/179,860) (0.37, 0.43)
**MCED cancer detection rate**		
First	0.67 (35/5229) (0.48, 0.93)	0.59 (384/65,096) (0.53, 0.65)
Second	0.67 (32/4746) (0.48, 0.95)	0.38 (229/59,752) (0.34, 0.44)
Third	0.31 (14/4523) (0.18, 0.52)	0.42 (243/57,800) (0.37, 0.48)
Aggregate	0.56 (81/14,498) (0.45, 0.69)	0.47 (856/18,2648) (0.44, 0.50)
**MCED cancer signal detection rate**		
First	1.84 (96/5229) (1.51, 2.24)	0.96 (626/65,096) (0.89, 1.04)
Second	1.56 (74/4746) (1.24, 1.95)	0.74 (444/59,752) (0.68, 0.82)
Third	1.37 (62/4523) (1.07, 1.75)	0.86 (499/57,800) (0.79, 0.94)
Aggregate	1.60 (232/14,498) (1.41, 1.82)	0.86 (1569/182,648) (0.82, 0.90)

MCED, multi-cancer early detection; NPV, negative predictive value; PPV, positive predictive value.

Supplementary Table S6. Performance Metrics by Subgroup: Race/Ethnicity.

Screening round	**White** Estimate (n/N) (95% CI)	**Mixed or multiple ethnic groups** Estimate (n/N) (95% CI)	**Black, African, Caribbean or Black British** Estimate (n/N) (95% CI)	**Asian or Asian British** Estimate (n/N) (95% CI)	**Other ethnic group** Estimate (n/N) (95% CI)
**PPV[a]**					
First[b]	58.7 (407/693) (55 62.3)	33.3 (2/6) (9.7, 70.0)	42.9 (3/7) (15.8, 75.0)	43.8 (7/16) (23.1, 66.8)	–
Second[c]	50.5 (253/501) (46.1, 54.9)	2/3	1/2	45.5 (5/11) (21.3, 72.0)	0/1
Third[d]	46.7 (249/533) (42.5, 51.0)	42.9 (3/7) (15.8, 75.0)	20.0 (1/5) (3.6, 62.4)	21.4 (3/14) (7.6, 47.6)	1/1
Aggregate[e]	52.6 (909/1727) (50.3, 55.0)	43.8 (7/16) (23.1, 66.8)	35.7 (5/14) (16.3, 61.2)	36.6 (15/41) (23.6, 51.9)	1/2
**NPV**					
First[b]	98.98 (64,470/65,134) (98.90, 99.05)	98.68 (749/759) (97.59, 99.28)	98.69 (983/996) (97.78, 99.24)	99.18 (2299/2318) (98.72, 99.47)	99.36 (309/311) (97.69, 99.82)
Second[c]	98.89 (59,588/60,259) (98.80, 98.97)	99.53 (634/637) (98.62, 99.84)	98.88 (793/802) (97.88, 99.41)	99.23 (1929/1944) (98.73, 99.53)	100 (249/249) (98.48, 100)
Third[d]	98.84 (57,436/58,110) (98.75, 98.92)	99.37 (629/633) (98.39, 99.75)	98.73 (779/789) (97.68, 99.31)	99.26 (1885/1899) (98.77, 99.56)	100 (248/248) (98.47, 100)

Screening round	White<br>Estimate (n/N)<br>(95% CI)	Mixed or multiple ethnic groups<br>Estimate (n/N)<br>(95% CI)	Black, African, Caribbean or Black British<br>Estimate (n/N)<br>(95% CI)	Asian or Asian British<br>Estimate (n/N)<br>(95% CI)	Other ethnic group<br>Estimate (n/N)<br>(95% CI)
Aggregate[e]	98.91 (181,494/183,503) (98.86, 98.95)	99.16 (2012/2029) (98.66, 99.48)	98.76 (2555/2587) (98.26, 99.12)	99.22 (6113/6161) (98.97, 99.41)	99.75 (806/808) (99.10, 99.93)
**Episode sensitivity[a]**					
First[b]	38.0 (407/1071) (35.1, 40.9)	16.7 (2/12) (4.7, 44.8)	18.8 (3/16) (6.6, 43.0)	26.9 (7/26) (13.7, 46.1)	0/2
Second[c]	27.4 (253/924) (24.6, 30.3)	40.0 (2/5) (11.8, 76.9)	10.0 (1/10) (1.8, 40.4)	25.0 (5/20) (11.2, 46.9)	–
Third[d]	27.0 (249/923) (24.2, 29.9)	42.9 (3/7) (15.8, 75.0)	9.1 (1/11) (1.6, 37.7)	17.6 (3/17) (6.2, 41.0)	1/1
Aggregate[e]	31.2 (909/2918) (29.5, 32.9)	29.2 (7/24) (14.9, 49.2)	13.5 (5/37) (5.9, 28.0)	23.8 (15/63) (15.0, 35.6)	1/3
**Specificity**					
First[b]	99.56 (64,470/64,756) (99.50, 99.61)	99.47 (749/753) (98.64, 99.79)	99.59 (983/987) (98.96, 99.84)	99.61 (2299/2308) (99.26, 99.79)	100 (309/309) (98.77, 100)
Second[c]	99.59 (59,588/59,836) (99.53, 99.63)	99.84 (634/635) (99.11, 99.97)	99.87 (793/794) (99.29, 99.98)	99.69 (1929/1935) (99.33, 99.86)	99.60 (249/250) (97.77, 99.93)
Third[d]	99.51 (57,436/57,720) (99.45, 99.56)	99.37 (629/633) (98.39, 99.75)	99.49 (779/783) (98.69, 99.80)	99.42 (1885/1896) (98.96, 99.68)	100 (248/248) (98.47, 100)

Screening round	**White** Estimate (n/N) (95% CI)	**Mixed or multiple ethnic groups** Estimate (n/N) (95% CI)	**Black, African, Caribbean or Black British** Estimate (n/N) (95% CI)	**Asian or Asian British** Estimate (n/N) (95% CI)	**Other ethnic group** Estimate (n/N) (95% CI)
Aggregate[e]	99.55 (181,494/182,312) (99.52, 99.58)	99.55 (2012/2021) (99.16, 99.77)	99.65 (2555/2564) (99.33, 99.82)	99.58 (6113/6139) (99.38, 99.71)	99.88 (806/807) (99.3, 99.98)
**False-positive rate**					
First[b]	0.44 (286/64,756) (0.39, 0.50)	0.53 (4/753) (0.21, 1.36)	0.41 (4/987) (0.16, 1.04)	0.39 (9/2308) (0.21, 0.74)	0 (0/309) (0, 1.23)
Second[c]	0.41 (248/59,836) (0.37, 0.47)	0.16 (1/635) (0.03, 0.89)	0.13 (1/794) (0.02, 0.71)	0.31 (6/1935) (0.14, 0.67)	0.40 (1/250) (0.07, 2.23)
Third[d]	0.49 (284/57,720) (0.44, 0.55)	0.63 (4/633) (0.25, 1.61)	0.51 (4/783) (0.20, 1.31)	0.58 (11/1896) (0.32, 1.04)	0 (0/248) (0, 1.53)
Aggregate[e]	0.45 (818/182,312) (0.42, 0.48)	0.45 (9/2,021) (0.23, 0.84)	0.35 (9/2564) (0.18, 0.67)	0.42 (26/6,139) (0.29, 0.62)	0.12 (1/807) (0.02, 0.70)
**MCED cancer detection rate**					
First[b]	0.62 (407/65,827) (0.56, 0.68)	0.26 (2/765) (0.07, 0.95)	0.30 (3/1003) (0.10, 0.88)	0.30 (7/2,334) (0.15, 0.62)	0 (0/311) (0, 1.22)
Second[c]	0.42 (253/60,760) (0.37, 0.47)	0.31 (2/640) (0.09, 1.13)	0.12 (1/804) (0.02, 0.70)	0.26 (5/1955) (0.11, 0.60)	0 (0/250) (0, 1.51)
Third[d]	0.42 (249/58,643) (0.38, 0.48)	0.47 (3/640) (0.16, 1.37)	0.13 (1/794) (0.02, 0.71)	0.16 (3/1931) (0.05, 0.46)	0.40 (1/249) (0.07, 2.24)
Aggregate[e]	0.49 (909/185,230) (0.46, 0.52)	0.34 (7/2045) (0.17, 0.70)	0.19 (5/2601) (0.08, 0.45)	0.24 (15/6202) (0.15, 0.40)	0.12 (1/810) (0.02, 0.70)

Screening round	**White** Estimate (n/N) (95% CI)	**Mixed or multiple ethnic groups** Estimate (n/N) (95% CI)	**Black, African, Caribbean or Black British** Estimate (n/N) (95% CI)	**Asian or Asian British** Estimate (n/N) (95% CI)	**Other ethnic group** Estimate (n/N) (95% CI)
**MCED cancer signal detection rate**					
First[b]	1.05 (693/65,827) (0.98, 1.13)	0.78 (6/765) (0.36, 1.70)	0.70 (7/1003) (0.34, 1.43)	0.69 (16/2334) (0.42, 1.11)	0 (0/311) (0, 1.22)
Second[c]	0.82 (501/60,760) (0.76, 0.90)	0.47 (3/640) (0.16, 1.37)	0.25 (2/804) (0.07, 0.90)	0.56 (11/1955) (0.31, 1.00)	0.40 (1/250) (0.07, 2.23)
Third[d]	0.91 (533/58,643) (0.84, 0.99)	1.09 (7/640) (0.53, 2.24)	0.63 (5/794) (0.27, 1.47)	0.73 (14/1913) (0.44, 1.22)	0.40 (1/249) (0.07, 2.24)
Aggregate[e]	0.93 (1,727/185,230) (0.89, 0.98)	0.78 (16/2045) (0.48, 1.27)	0.54 (14/2601) (0.32, 0.90)	0.66 (41/6202) (0.49, 0.90)	0.25 (2/810) (0.07, 0.90)

Metrics are not reported if N = 0.

[a]Metrics are not estimated for subgroups with less than 5 cases.

[b]85 participants with missing subgroup values are not included.

[c]89 participants with missing subgroup values are not included.

[d]84 participants with missing subgroup values are not included.

[e]Screening episodes from participants with missing subgroup values are not included.

MCED, multi-cancer early detection; NPV, negative predictive value; PPV, positive predictive value.

Supplementary Table S7. Performance Metrics by Subgroup: Index of Multiple Deprivation.

**Screening round**	**Quintile 1** Estimate (n/N) (95% CI)	**Quintile 2** Estimate (n/N) (95% CI)	**Quintile 3** Estimate (n/N) (95% CI)	**Quintile 4** Estimate (n/N) (95% CI)	**Quintile 5** Estimate (n/N) (95% CI)
**PPV**					
First[a]	56.7 (93/164) (49.1, 64.1)	55.2 (79/143) (47.1, 63.2)	58.8 (80/136) (50.4, 66.7)	61.6 (90/146) (53.6, 69.1)	57.9 (77/133) (49.4, 65.9)
Second[b]	48.2 (54/112) (39.2, 57.4)	52.5 (52/99) (42.8, 62.1)	52.2 (59/113) (43.1, 61.2)	51.0 (53/104) (41.5, 60.4)	48.3 (43/89) (38.2, 58.5)
Third[c]	45.8 (60/131) (37.5, 54.3)	40.2 (47/117) (31.7, 49.2)	51.3 (58/113) (42.2, 60.3)	50.0 (50/100) (40.4, 59.6)	41.8 (41/98) (32.6, 51.7)
Aggregate[d]	50.9 (207/407) (46.0, 55.7)	49.6 (178/359) (44.4, 54.7)	54.4 (197/362) (49.3, 59.5)	55.1 (193/350) (49.9, 60.3)	50.3 (161/320) (44.9, 55.8)
**NPV**					
First[a]	99.04 (15,548/15,699) (98.87, 99.18)	98.89 (13,486/13,637) (98.70, 99.06)	99.11 (14,620/14,751) (98.95, 99.25)	98.99 (13,927/14,069) (98.81, 99.14)	98.84 (11,085/11,215) (98.63, 99.02)
Second[b]	98.97 (13,775/13,918) (98.79, 99.13)	98.92 (12,298/12,432) (98.72, 99.09)	99.00 (13,525/13,661) (98.82, 99.16)	98.80 (13,023/13,181) (98.60, 98.97)	98.77 (10,456/10,586) (98.54, 98.96)
Third[c]	98.95 (13,275/13,416) (98.76, 99.11)	98.89 (11,809/11,942) (98.68, 99.06)	98.75 (13,071/13,237) (98.54, 98.92)	98.87 (12,596/12,740) (98.67, 99.04)	98.83 (10,106/10,226) (98.60, 99.02)
Aggregate[d]	98.99 (42,598/43,033) (98.89, 99.08)	98.90 (37,593/38,011) (98.79, 99.00)	98.96 (41,216/41,649) (98.86, 99.05)	98.89 (39,546/39,990) (98.78, 98.99)	98.81 (31,647/32,027) (98.69, 98.93)

Screening round	**Quintile 1** Estimate (n/N) (95% CI)	**Quintile 2** Estimate (n/N) (95% CI)	**Quintile 3** Estimate (n/N) (95% CI)	**Quintile 4** Estimate (n/N) (95% CI)	**Quintile 5** Estimate (n/N) (95% CI)
**Episode sensitivity**					
First[a]	38.1 (93/244) (32.2, 44.3)	34.3 (79/230) (28.5, 40.7)	37.9 (80/211) (31.6, 44.6)	38.8 (90/232) (32.8, 45.2)	37.2 (77/207) (30.9, 44.0)
Second[b]	27.4 (54/197) (21.7, 34.0)	28 (52/186) (22.0, 34.8)	30.3 (59/195) (24.2, 37.0)	25.1 (53/211) (19.7, 31.4)	24.9 (43/173) (19.0, 31.8)
Third[c]	29.9 (60/201) (24.0, 36.5)	26.1 (47/180) (20.2, 33.0)	25.9 (58/224) (20.6, 32.0)	25.8 (50/194) (20.1, 32.4)	25.5 (41/161) (19.4, 32.7)
Aggregate[d]	32.2 (207/642) (28.7, 36.0)	29.9 (178/596) (26.3, 33.7)	31.3 (197/630) (27.8, 35.0)	30.3 (193/637) (26.9, 34.0)	29.8 (161/541) (26.1, 33.7)
**Specificity**					
First[a]	99.55 (15,548/15,619) (99.43, 99.64)	99.53 (13,486/13,550) (99.40, 99.63)	99.62 (14,620/14,676) (99.50, 99.71)	99.60 (13,927/13,983) (99.48, 99.69)	99.50 (11,085/11,141) (99.35, 99.61)
Second[b]	99.58 (13,775/13,833) (99.46, 99.68)	99.62 (12,298/12,345) (99.49, 99.71)	99.60 (13,525/13,579) (99.48, 99.70)	99.61 (13,023/13,074) (99.49, 99.70)	99.56 (10,456/10,502) (99.42, 99.67)
Third[c]	99.47 (13,275/13,346) (99.33, 99.58)	99.41 (11,809/11,879) (99.26, 99.53)	99.58 (13,071/13,126) (99.46, 99.68)	99.60 (12,596/12,646) (99.48, 99.70)	99.44 (10,106/10,163) (99.27, 99.57)
Aggregate[d]	99.53 (42,598/42,798) (99.46, 99.59)	99.52 (37,593/37,774) (99.45, 99.59)	99.60 (41,216/41,381) (99.54, 99.66)	99.60 (39,546/39,703) (99.54, 99.66)	99.50 (31,647/31,806) (99.42, 99.57)
**False-positive rate**					

Screening round	**Quintile 1** Estimate (n/N) (95% CI)	**Quintile 2** Estimate (n/N) (95% CI)	**Quintile 3** Estimate (n/N) (95% CI)	**Quintile 4** Estimate (n/N) (95% CI)	**Quintile 5** Estimate (n/N) (95% CI)
First[a]	0.45 (71/15,619) (0.36, 0.57)	0.47 (64/13,550) (0.37, 0.60)	0.38 (56/14,676) (0.29, 0.50)	0.40 (56/13,983) (0.31, 0.52)	0.50 (56/11,141) (0.39, 0.65)
Second[b]	0.42 (58/13,833) (0.32, 0.54)	0.38 (47/12,345) (0.29, 0.51)	0.40 (54/13,579) (0.30, 0.52)	0.39 (51/13,074) (0.30, 0.51)	0.44 (46/10,502) (0.33, 0.58)
Third[c]	0.53 (71/13,346) (0.42, 0.67)	0.59 (70/11,879) (0.47, 0.74)	0.42 (55/13,126) (0.32, 0.54)	0.40 (50/12,646) (0.30, 0.52)	0.56 (57/10,163) (0.43, 0.73)
Aggregate[d]	0.47 (200/42,798) (0.41, 0.54)	0.48 (181/37,774) (0.41, 0.55)	0.40 (165/41,381) (0.34, 0.46)	0.40 (157/39,703) (0.34, 0.46)	0.50 (159/31,806) (0.43, 0.58)
**MCED cancer detection rate**					
First[a]	0.59 (93/15,863) (0.48, 0.72)	0.57 (79/13,780) (0.46, 0.71)	0.54 (80/14,887) (0.43, 0.67)	0.63 (90/14,215) (0.52, 0.78)	0.68 (77/11,348) (0.54, 0.85)
Second[b]	0.38 (54/14,030) (0.30, 0.50)	0.41 (52/12,531) (0.32, 0.54)	0.43 (59/13,774) (0.33, 0.55)	0.40 (53/13,285) (0.31, 0.52)	0.40 (43/10,675) (0.30, 0.54)
Third[c]	0.44 (60/13,547) (0.34, 0.57)	0.39 (47/12,059) (0.29, 0.52)	0.43 (58/13,350) (0.34, 0.56)	0.39 (50/12,840) (0.30, 0.51)	0.40 (41/10,324) (0.29, 0.54)
Aggregate[d]	0.48 (207/43,440) (0.42, 0.55)	0.46 (178/38,370) (0.40, 0.54)	0.47 (197/42,011) (0.41, 0.54)	0.48 (193/40,340) (0.42, 0.55)	0.50 (161/32,347) (0.43, 0.58)
**MCED cancer signal detection rate**					
First[a]	1.03 (164/15,863) (0.89, 1.20)	1.04 (143/13,780) (0.88, 1.22)	0.91 (136/14,887) (0.77, 1.08)	1.03 (146/14,215) (0.87, 1.21)	1.17 (133/11,348) (0.99, 1.39)
Second[b]	0.80 (112/14,030) (0.66, 0.96)	0.79 (99/12,531) (0.65, 0.96)	0.82 (113/13,774) (0.68, 0.99)	0.78 (104/13,285) (0.65, 0.95)	0.83 (89/10,675) (0.68, 1.02)

Screening round	**Quintile 1** Estimate (n/N) (95% CI)	**Quintile 2** Estimate (n/N) (95% CI)	**Quintile 3** Estimate (n/N) (95% CI)	**Quintile 4** Estimate (n/N) (95% CI)	**Quintile 5** Estimate (n/N) (95% CI)
Third[c]	0.97 (131/13,547) (0.82, 1.15)	0.97 (117/12,059) (0.81, 1.16)	0.85 (113/13,350) (0.70, 1.02)	0.78 (100/12,840) (0.64, 0.95)	0.95 (98/10,324) (0.78, 1.16)
Aggregate[d]	0.94 (407/43,440) (0.85, 1.03)	0.94 (359/38,370) (0.84, 1.04)	0.86 (362/42,011) (0.78, 0.95)	0.87 (350/40,340) (0.78, 0.96)	0.99 (320/32,347) (0.89, 1.10)

[a]232 participants with missing subgroup values are not included.

[b]203 participants with missing subgroup values are not included.

[c]203 participants with missing subgroup values are not included.

[d]Screening episodes from participants with missing subgroup values are not included.

MCED, multi-cancer early detection; IMD, Index of Multiple Deprivation; NPV, negative predictive value; PPV, positive predictive value.

Supplementary Table S8. Performance Metrics by Subgroup: Education.

**Screening round**	**School at or before the age of 15** Estimate (n/N) (95% CI)	**CSEs, O-levels or equivalent** Estimate (n/N) (95% CI)	**A-levels or equivalent** Estimate (n/N) (95% CI)	**Further education without a degree** Estimate (n/N) (95% CI)	**Bachelor's degree or equivalent** Estimate (n/N) (95% CI)	**Further degree e.g., Masters or PhD** Estimate (n/N) (95% CI)
**PPV**						
First[a]	56.2 (91/162) (48.5, 63.6)	59.1 (94/159) (51.4, 66.5)	60.3 (35/58) (47.5, 71.9)	57.3 (94/164) (49.7, 64.6)	57.3 (67/117) (48.2, 65.9)	61.3 (38/62) (48.8, 72.4)
Second[b]	56.5 (65/115) (47.4, 65.2)	51.8 (57/110) (42.6, 60.9)	50.8 (31/61) (38.6, 62.9)	41.7 (48/115) (33.1, 50.9)	51.3 (40/78) (40.4, 62.1)	51.3 (20/39) (36.2, 66.1)
Third[c]	53.5 (61/114) (44.4, 62.4)	45.6 (62/136) (37.5, 54.0)	46.3 (25/54) (33.7, 59.4)	42.1 (59/140) (34.3, 50.4)	43.2 (32/74) (32.6, 54.6)	42.9 (18/42) (29.1, 57.8)
Aggregate[d]	55.5 (217/391) (50.5, 60.3)	52.6 (213/405) (47.7, 57.4)	52.6 (91/173) (45.2, 59.9)	48.0 (201/419) (43.2, 52.8)	51.7 (139/269) (45.7, 57.6)	53.1 (76/143) (45.0, 61.1)
**NPV**						
First[a]	98.91 (12,240/12,375) (98.71, 99.08)	99.11 (18,724/18,892) (98.97, 99.23)	98.93 (6314/6382) (98.65, 99.16)	98.95 (14,294/14,446) (98.77, 99.10)	98.90 (11,590/11,719) (98.69, 99.07)	99.02 (5648/5704) (98.73, 99.24)
Second[b]	98.52 (10,951/11,116) (98.27, 98.72)	99.10 (17,244/17,401) (98.95, 99.23)	98.78 (5763/5834) (98.47, 99.03)	98.98 (13,265/13,402) (98.79, 99.13)	99.02 (10,776/10,883) (98.81, 99.19)	98.84 (5194/5255) (98.51, 99.10)
Third[c]	98.44 (10,506/10,673)	99.08 (16,675/16,829)	98.79 (5549/5617)	98.86 (12,795/12,942)	98.99 (10,436/10,543)	98.84 (5016/5075)

**Screening round**	**School at or before the age of 15** Estimate (n/N) (95% CI)	**CSEs, O-levels or equivalent** Estimate (n/N) (95% CI)	**A-levels or equivalent** Estimate (n/N) (95% CI)	**Further education without a degree** Estimate (n/N) (95% CI)	**Bachelor's degree or equivalent** Estimate (n/N) (95% CI)	**Further degree e.g., Masters or PhD** Estimate (n/N) (95% CI)
	(98.18, 98.65)	(98.93, 99.22)	(98.47, 99.04)	(98.67, 99.03)	(98.78, 99.16)	(98.50, 99.10)
Aggregate[d]	98.63 (33,697/34,164) (98.50, 98.75)	99.10 (52,643/53,122) (99.01, 99.18)	98.84 (17,626/17,833) (98.67, 98.99)	98.93 (40,354/40,790) (98.83, 99.03)	98.97 (32,802/33,145) (98.85, 99.07)	98.9 (15,858/16,034) (98.73, 99.05)
**Episode sensitivity**						
First[a]	40.3 (91/226) (34.1, 46.8)	35.9 (94/262) (30.3, 41.9)	34.0 (35/103) (25.6, 43.6)	38.2 (94/246) (32.4, 44.4)	34.2 (67/196) (27.9, 41.1)	40.4 (38/94) (31.1, 50.5)
Second[b]	28.3 (65/230) (22.8, 34.4)	26.6 (57/214) (21.2, 32.9)	30.4 (31/102) (22.3, 39.9)	25.9 (48/185) (20.2, 32.7)	27.2 (40/147) (20.7, 34.9)	24.7 (20/81) (16.6, 35.1)
Third[c]	26.8 (61/228) (21.4, 32.9)	28.7 (62/216) (23.1, 35.1)	26.9 (25/93) (18.9, 36.7)	28.6 (59/206) (22.9, 35.2)	23.0 (32/139) (16.8, 30.7)	23.4 (18/77) (15.3, 34.0)
Aggregate[d]	31.7 (217/684) (28.3, 35.3)	30.8 (213/692) (27.5, 34.3)	30.5 (91/298) (25.6, 36.0)	31.6 (201/637) (28.1, 35.3)	28.8 (139/482) (25.0, 33.0)	30.2 (76/252) (24.8, 36.1)
**Specificity**						
First[a]	99.42 (12,240/12,311) (99.27, 99.54)	99.65 (18,724/18,789) (99.56, 99.73)	99.64 (6314/6337) (99.46, 99.76)	99.51 (14,294/14,364) (99.38, 99.61)	99.57 (11,590/11,640) (99.43, 99.67)	99.58 (5648/5672) (99.37, 99.72)
Second[b]	99.55 (10,951/11,001)	99.69 (17,244/17,297)	99.48 (5763/5793)	99.50 (13,265/13,332)	99.65 (10,776/10,814)	99.64 (5194/5213)

**Screening round**	**School at or before the age of 15** Estimate (n/N) (95% CI)	**CSEs, O-levels or equivalent** Estimate (n/N) (95% CI)	**A-levels or equivalent** Estimate (n/N) (95% CI)	**Further education without a degree** Estimate (n/N) (95% CI)	**Bachelor's degree or equivalent** Estimate (n/N) (95% CI)	**Further degree e.g., Masters or PhD** Estimate (n/N) (95% CI)
	(99.40, 99.66)	(99.60, 99.77)	(99.26, 99.64)	(99.36, 99.60)	(99.52, 99.74)	(99.43, 99.77)
Third[c]	99.50 (10,506/10,559) (99.34, 99.62)	99.56 (16,675/16,749) (99.45, 99.65)	99.48 (5549/5578) (99.25, 99.64)	99.37 (12,795/12,876) (99.22, 99.49)	99.60 (10,436/10,478) (99.46, 99.70)	99.52 (5016/5040) (99.29, 99.68)
Aggregate[d]	99.49 (33,697/33,871) (99.40, 99.56)	99.64 (52,643/52,835) (99.58, 99.68)	99.54 (17,626/17,708) (99.43, 99.63)	99.46 (40,354/40,572) (99.39, 99.53)	99.61 (32,802/32,932) (99.53, 99.67)	99.58 (15,858/15,925) (99.47, 99.67)
**False-positive rate**						
First[a]	0.58 (71/12,311) (0.46, 0.73)	0.35 (65/18,789) (0.27, 0.44)	0.36 (23/6337) (0.24, 0.54)	0.49 (70/14,364) (0.39, 0.62)	0.43 (50/11,640) (0.33, 0.57)	0.42 (24/5672) (0.28, 0.63)
Second[b]	0.45 (50/11,001) (0.34, 0.60)	0.31 (53/17,297) (0.23, 0.40)	0.52 (30/5793) (0.36, 0.74)	0.50 (67/13,332) (0.40, 0.64)	0.35 (38/10,814) (0.26, 0.48)	0.36 (19/5213) (0.23, 0.57)
Third[c]	0.50 (53/10,559) (0.38, 0.66)	0.44 (74/16,749) (0.35, 0.55)	0.52 (29/5578) (0.36, 0.75)	0.63 (81/12,876) (0.51, 0.78)	0.40 (42/10,478) (0.30, 0.54)	0.48 (24/5040) (0.32, 0.71)
Aggregate[d]	0.51 (174/33,871) (0.44, 0.60)	0.36 (192/52,835) (0.32, 0.42)	0.46 (82/17,708) (0.37, 0.57)	0.54 (218/40,572) (0.47, 0.61)	0.39 (130/32,932) (0.33, 0.47)	0.42 (67/15,925) (0.33, 0.53)

Screening round	School at or before the age of 15 Estimate (n/N) (95% CI)	CSEs, O-levels or equivalent Estimate (n/N) (95% CI)	A-levels or equivalent Estimate (n/N) (95% CI)	Further education without a degree Estimate (n/N) (95% CI)	Bachelor's degree or equivalent Estimate (n/N) (95% CI)	Further degree e.g., Masters or PhD Estimate (n/N) (95% CI)
**MCED cancer detection rate**						
First[a]	0.73 (91/12,537) (0.59, 0.89)	0.49 (94/19,051) (0.40, 0.60)	0.54 (35/6440) (0.39, 0.75)	0.64 (94/14,610) (0.53, 0.79)	0.57 (67/11,836) (0.45, 0.72)	0.66 (38/5766) (0.48, 0.90)
Second[b]	0.580 (65/11,231) (0.45, 0.74)	0.33 (57/17,511) (0.25, 0.42)	0.53 (31/5,895) (0.37, 0.75)	0.36 (48/13,517) (0.27, 0.47)	0.36 (40/10,961) (0.27, 0.50)	0.38 (20/5,294) (0.24, 0.58)
Third[c]	0.57 (61/10,787) (0.44, 0.73)	0.37 (62/16,965) (0.29, 0.47)	0.44 (25/5671) (0.30, 0.65)	0.45 (59/13,082) (0.35, 0.58)	0.30 (32/10,617) (0.21, 0.43)	0.35 (18/5117) (0.22, 0.56)
Aggregate[d]	0.63 (217/34,555) (0.55, 0.72)	0.40 (213/53,527) (0.35, 0.45)	0.51 (91/18,006) (0.41, 0.62)	0.49 (201/41,209) (0.42, 0.56)	0.42 (139/33,414) (0.35, 0.49)	0.47 (76/16,177) (0.38, 0.59)
**MCED cancer signal detection rate**						
First[a]	1.29 (162/12,537) (1.11, 1.51)	0.83 (159/19,051) (0.71, 0.97)	0.90 (58/6,440) (0.70, 1.16)	1.12 (164/14,610) (0.96, 1.31)	0.99 (117/11,836) (0.83, 1.18)	1.08 (62/5,766) (0.84, 1.38)
Second[b]	1.02 (115/11,231) (0.85, 1.23)	0.63 (110/17,511) (0.52, 0.76)	1.03 (61/5895) (0.81, 1.33)	0.85 (115/13,517) (0.71, 1.02)	0.71 (78/10,961) (0.57, 0.89)	0.74 (39/5294) (0.54, 1.01)

**Screening round**	**School at or before the age of 15** Estimate (n/N) (95% CI)	**CSEs, O-levels or equivalent** Estimate (n/N) (95% CI)	**A-levels or equivalent** Estimate (n/N) (95% CI)	**Further education without a degree** Estimate (n/N) (95% CI)	**Bachelor's degree or equivalent** Estimate (n/N) (95% CI)	**Further degree e.g., Masters or PhD** Estimate (n/N) (95% CI)
Third[c]	1.06 (114/10,787) (0.88, 1.27)	0.80 (136/16,965) (0.68, 0.95)	0.95 (54/5671) (0.73, 1.24)	1.07 (140/13,082) (0.91, 1.26)	0.70 (74/10,617) (0.56, 0.87)	0.82 (42/5117) (0.61, 1.11)
Aggregate[d]	1.13 (391/34,555) (1.03, 1.25)	0.76 (405/53,527) (0.69, 0.83)	0.96 (173/18,006) (0.83, 1.11)	1.02 (419/41,209) (0.92, 1.12)	0.81 (269/33,414) (0.71, 0.91)	0.88 (143/16,177) (0.75, 1.04)

[a]85 participants with missing subgroup values are not included.

[b]89 participants with missing subgroup values are not included.

[c]84 participants with missing subgroup values are not included.

[d]Screening episodes from participants with missing subgroup values are not included.

A-levels, advanced levels; CSE, certificate of secondary education; MCED, multi-cancer early detection; NPV, negative predictive value; O-levels, ordinary levels; PPV, positive predictive value.

Supplementary Table S9. PPV by CSO Prediction by Round and in Aggregate.

MCED CSO1 prediction	Positive MCED result, cancer diagnosis, n (%)	Positive MCED result, no cancer diagnosis, n (%)	PPV,[a] % (n/N) (95% CI)
**First screening round (n=722)**			
Total, n	419	303	
Anus	2 (0.5)	3 (1.0)	40.0 (2/5) (11.8, 76.9)
Bladder, Urothelial Tract	2 (0.5)	2 (0.7)	2/4
Bone and Soft Tissue	1 (0.2)	2 (0.7)	1/3
Breast	26 (6.2)	29 (9.6)	47.3 (26/55) (34.7, 60.2)
Cervix	0	1 (0.3)	0/1
Colon, Rectum	84 (20.0)	79 (26.1)	51.5 (84/163) (43.9, 59.1)
Head and Neck	24 (5.7)	17 (5.6)	58.5 (24/41) (43.4, 72.2)
Kidney	10 (2.4)	2 (0.7)	83.3 (10/12) (55.2, 95.3)
Liver, Bile Duct	13 (3.1)	6 (2.0)	68.4 (13/19) (46.0, 84.6)
Lung	53 (12.6)	28 (9.2)	65.4 (53/81) (54.6, 74.9)
Lymphoid Lineage	57 (13.6)	67 (22.1)	46.0 (57/124) (37.4, 54.7)
Melanocytic Lineage	2 (0.5)	0	2/2
Neuroendocrine Cells of the Lung or Other Organs	8 (1.9)	1 (0.3)	88.9 (8/9) (56.5, 98.0)
Ovary	15 (3.6)	1 (0.3)	93.8 (15/16) (71.7, 98.9)

MCED CSO1 prediction	**Positive MCED result, cancer diagnosis,** n (%)	**Positive MCED result, no cancer diagnosis,** n (%)	**PPV,[a] %** (n/N) (95% CI)
Pancreas, Gallbladder	23 (5.5)	7 (2.3)	76.7 (23/30) (59.1, 88.2)
Plasma Cell Lineage	24 (5.7)	20 (6.6)	54.5 (24/44) (40.1, 68.3)
Prostate	39 (9.3)	22 (7.3)	63.9 (39/61) (51.4, 74.8)
Stomach, Oesophagus	27 (6.4)	13 (4.3)	67.5 (27/40) (52.0, 79.9)
Thyroid Gland	0	1 (0.3)	0/1
Uterus	9 (2.1)	2 (0.7)	81.8 (9/11) (52.3, 94.9)
**Second screening round (n=518)**			
Total, n	261	257	
Anus	3 (1.1)	2 (0.8)	60.0 (3/5) (23.1, 88.2)
Bladder, Urothelial Tract	8 (3.1)	1 (0.4)	88.9 (8/9) (56.5, 98.0)
Bone and Soft Tissue	0	2 (0.8)	0/2
Breast	18 (6.9)	25 (9.7)	41.9 (18/43) (28.4, 56.7)
Cervix	1 (0.4)	0	1/1
Colon, Rectum	58 (22.2)	69 (26.8)	45.7 (58/127) (37.3, 54.3)
Head and Neck	11 (4.2)	14 (5.4)	44.0 (11/25) (26.7, 62.9)
Kidney	0	3 (1.2)	0/3
Liver, Bile Duct	5 (1.9)	2 (0.8)	71.4 (5/7) (35.9, 91.8)
Lung	33 (12.6)	25 (9.7)	56.9 (33/58) (44.1, 68.8)

MCED CSO1 prediction	**Positive MCED result, cancer diagnosis,** n (%)	**Positive MCED result, no cancer diagnosis,** n (%)	**PPV,**[a] **%** (n/N) (95% CI)
Lymphoid Lineage	29 (11.1)	41 (16.0)	41.4 (29/70) (30.6, 53.1)
Melanocytic Lineage	1 (0.4)	0	1/1
Neuroendocrine Cells of Lung or Other Organs	8 (3.1)	2 (0.8)	80.0 (8/10) (49.0, 94.3)
Ovary	6 (2.3)	0	100 (6/6) (61.0, 100)
Pancreas, Gallbladder	15 (5.7)	12 (4.7)	55.6 (15/27) (37.3, 72.4)
Plasma Cell Lineage	10 (3.8)	17 (6.6)	37.0 (10/27) (21.5, 55.8)
Prostate	30 (11.5)	23 (8.9)	56.6 (30/53) (43.3, 69.0)
Stomach, Oesophagus	18 (6.9)	12 (4.7)	60.0 (18/30) (42.3, 75.4)
Uterus	7 (2.7)	7 (2.7)	50.0 (7/14) (26.8, 73.2)
**Third screening round (n=561)**			
Total, n	257	304	
Anus	1 (0.4)	2 (0.7)	1/3
Bladder, Urothelial Tract	4 (1.6)	2 (0.7)	66.7 (4/6) (30.0, 90.3)
Breast	19 (7.4)	26 (8.6)	42.2 (19/45) (29.0, 56.7)
Cervix	0	1 (0.3)	0/1
Colon, Rectum	49 (19.1)	69 (22.7)	41.5 (49/118) (33.0, 50.5)
Head and Neck	13 (5.1)	17 (5.6)	43.3 (13/30) (27.4, 60.8)

MCED CSO1 prediction	Positive MCED result, cancer diagnosis, n (%)	Positive MCED result, no cancer diagnosis, n (%)	PPV,[a] % (n/N) (95% CI)
Kidney	4 (1.6)	3 (1.0)	57.1 (4/7) (25.0, 84.2)
Liver, Bile Duct	12 (4.7)	9 (3.0)	57.1 (12/21) (36.5, 75.5)
Lung	37 (14.4)	31 (10.2)	54.4 (37/68) (42.7, 65.7)
Lymphoid Lineage	26 (10.1)	59 (19.4)	30.6 (26/85) (21.8, 41.0)
Melanocytic Lineage	2 (0.8)	2 (0.7)	2/4
Neuroendocrine Cells of Lung or Other Organs	8 (3.1)	2 (0.7)	80.0 (8/10) (49.0, 94.3)
Ovary	7 (2.7)	1 (0.3)	87.5 (7/8) (52.9, 97.8)
Pancreas, Gallbladder	16 (6.2)	11 (3.6)	59.3 (16/27) (40.7, 75.5)
Plasma Cell Lineage	13 (5.1)	18 (5.9)	41.9 (13/31) (26.4, 59.2)
Prostate	20 (7.8)	23 (7.6)	46.5 (20/43) (32.5, 61.1)
Stomach, Oesophagus	19 (7.4)	10 (3.3)	65.5 (19/29) (47.3, 80.1)
Uterus	7 (2.7)	18 (5.9)	28.0 (7/25) (14.3, 47.6)
**Aggregate (N=1801)**			
Total, n	937	864	
Anus	6 (0.6)	7 (0.8)	46.2 (6/13) (23.2, 70.9)
Bladder, Urothelial Tract	14 (1.5)	5 (0.6)	73.7 (14/19) (51.2, 88.2)

MCED CSO1 prediction	**Positive MCED result, cancer diagnosis,** n (%)	**Positive MCED result, no cancer diagnosis,** n (%)	**PPV,[a] %** (n/N) (95% CI)
Bone and Soft Tissue	1 (0.1)	4 (0.5)	20.0 (1/5) (3.6, 62.4)
Breast	63 (6.7)	80 (9.3)	44.1 (63/143) (36.2, 52.2)
Cervix	1 (0.1)	2 (0.2)	1/3
Colon, Rectum	191 (20.4)	217 (25.1)	46.8 (191/408) (42.0, 51.7)
Head and Neck	48 (5.1)	48 (5.6)	50.0 (48/96) (40.2, 59.8)
Kidney	14 (1.5)	8 (0.9)	63.6 (14/22) (43.0, 80.3)
Liver, Bile Duct	30 (3.2)	17 (2.0)	63.8 (30/47) (49.5, 76.0)
Lung	123 (13.1)	84 (9.7)	59.4 (123/207) (52.6, 65.9)
Lymphoid Lineage	112 (12.0)	167 (19.3)	40.1 (112/279) (34.6, 46.0)
Melanocytic Lineage	5 (0.5)	2 (0.2)	71.4 (5/7) (35.9, 91.8)
Neuroendocrine Cells of Lung or other Organs	24 (2.6)	5 (0.6)	82.8 (24/29) (65.5, 92.4)
Ovary	28 (3.0)	2 (0.2)	93.3 (28/30) (78.7, 98.2)
Pancreas, Gallbladder	54 (5.8)	30 (3.5)	64.3 (54/84) (53.6, 73.7)
Plasma Cell Lineage	47 (5.0)	55 (6.4)	46.1 (47/102) (36.7, 55.7)
Prostate	89 (9.5)	68 (7.9)	56.7 (89/157) (48.9, 64.2)
Stomach, Oesophagus	64 (6.8)	35 (4.1)	64.6 (64/99) (54.8, 73.4)

**MCED CSO1 prediction**	**Positive MCED result, cancer diagnosis,** n (%)	**Positive MCED result, no cancer diagnosis,** n (%)	**PPV,[a] %** (n/N) (95% CI)
Thyroid Gland	0	1 (0.1)	0/1
Uterus	23 (2.5)	27 (3.1)	46.0 (23/50) (33.0, 59.6)

[a]PPV is not estimated for CSOs with less than 5 cases.

CSO, cancer signal origin; MCED, multi-cancer detection; PPV, positive predictive value.

Supplementary Table S10. Characteristics of First Primary Cancers in Participants.

Cancer type diagnosed	Stage,[a] n (%)						Total, n (%)
	I	II	III	IV	Unstageable[b]	Missing	
**First screening round**							
**Participants with positive MCED test results and cancer diagnosis**							
Total	88 (21.0)	78 (18.6)	123 (29.4)	103 (24.6)	4 (1.0)	23 (5.5)	419
Anus	0	0	2 (66.7)	1 (33.3)	0	0	3 (0.7)
Bladder	1 (50.0)	1 (50.0)	0	0	0	0	2 (0.5)
Breast	6 (20.7)	13 (44.8)	5 (17.2)	4 (13.8)	0	1 (3.4)	29 (6.9)
Cervix	0	0	0	1 (100)	0	0	1 (0.2)
Colon/Rectum	21 (24.7)	22 (25.9)	25 (29.4)	15 (17.6)	0	2 (2.4)	85 (20.3)
Gallbladder	0	2 (40.0)	2 (40.0)	1 (20.0)	0	0	5 (1.2)
Head and Neck	10 (62.5)	1 (6.2)	4 (25.0)	1 (6.2)	0	0	16 (3.8)
Kidney	0	3 (33.3)	2 (22.2)	3 (33.3)	0	1 (11.1)	9 (2.1)
Liver/Bile-duct	4 (30.8)	5 (38.5)	1 (7.7)	3 23.1)	0	0	13 (3.1)
Lung	4 (7.5)	4 (7.5)	24 (45.3)	21 (39.6)	0	0	53 (12.6)
Lymphoid Leukaemia	10 (71.4)	2 (14.3)	0	0	0	2 (14.3)	14 (3.3)
Lymphoma	6 (18.2)	4 (12.1)	12 (36.4)	11 (33.3)	0	0	33 (7.9)

Cancer type diagnosed	Stage,[a] n (%)						Total, n (%)
	I	II	III	IV	Unstageable[b]	Missing	
Melanoma	1 (33.3)	0	1 (33.3)	1 (33.3)	0	0	3 (0.7)
Myeloid Neoplasm	0	0	0	0	1 (100)	0	1 (0.2)
Oesophagus	1 (5.0)	2 (10.0)	12 (60.0)	4 (20.0)	0	1 (5.0)	20 (4.8)
Ovary	2 (10.5)	2 (10.5)	13 (68.4)	2 (10.5)	0	0	19 (4.5)
Pancreas	2 (11.1)	2 (11.1)	2 (11.1)	11 (61.1)	0	1 (5.6)	18 (4.3)
Plasma Cell Neoplasm	4 (19.0)	3 (14.3)	0	0	0	14 (66.7)	21 (5.0)
Prostate	11 (22.9)	3 (6.2)	16 (33.3)	18 (37.5)	0	0	48 (11.5)
Sarcoma	0	0	0	1 (50.0)	1 (50.0)	0	2 (0.5)
Stomach	0	5 (55.6)	2 (22.2)	2 (22.2)	0	0	9 (2.1)
Thyroid	0	1 (100)	0	0	0	0	1 (0.2)
Uterus	3 (42.9)	2 (28.6)	0	2 (28.6)	0	0	7 (1.7)
Other[c]	2 (50.0)	0	0	0	1 (25.0)	1 (25.0)	4 (1.0)
Multiple cancers	0	1 (100)	0	0	0	0	1 (0.2)
NCRAS Cancer of Unknown Primary[d]	0	0	0	1 (50.0)	1 (50.0)	0	2 (0.5)

Cancer type diagnosed	Stage,[a] n (%)						Total, n (%)
	I	II	III	IV	Unstageable[b]	Missing	
**Participants with negative MCED test results whose cancer diagnosis was triggered by NHS cancer screening**							
Total	51 (69.9)	18 (24.7)	2 (2.7)	2 (2.7)	0	0	73
Breast	48 (72.7)	17 (25.8)	1 (1.5)	0	0	0	66 (90.4)
Colon/Rectum	3 (50.0)	1 (16.7)	1 (16.7)	1 (16.7)	0	0	6 (8.2)
Lung	0	0	0	1 (100)	0	0	1 (1.4)
**Participants with negative MCED test results whose cancer diagnosis was not triggered by NHS cancer screening**							
Total	294 (46.3)	115 (18.1)	120 (18.9)	54 (8.5)	34 (5.4)	18 (2.8)	635
Anus	0	0	2 (100)	0	0	0	2 (0.3)
Bladder	8 (57.1)	4 (28.6)	1 (7.1)	1 (7.1)	0	0	14 (2.2)
Breast	20 (35.1)	31 (54.4)	3 (5.3)	2 (3.5)	0	1 (1.8)	57 (9.0)
Cervix	0	1 (100)	0	0	0	0	1 (0.2)
Colon/Rectum	20 (55.6)	5 (13.9)	6 (16.7)	5 (13.9)	0	0	36 (5.7)
Gallbladder	1 (25.0)	1 (25.0)	1 (25.0)	0	0	1 (25.0)	4 (0.6)
Head and Neck	5 (45.5)	2 (18.2)	2 (18.2)	2 (18.2)	0	0	11 (1.7)
Kidney	10 (45.5)	2 (9.1)	8 (36.4)	2 (9.1)	0	0	22 (3.5)
Liver/Bile-duct	1 (33.3)	1 (33.3)	0	1 (33.3)	0	0	3 (0.5)
Lung	12 (42.9)	2 (7.1)	5 (17.9)	9 (32.1)	0	0	28 (4.4)

Cancer type diagnosed	Stage,[a] n (%)						Total, n (%)
	I	II	III	IV	Unstageable[b]	Missing	
Lymphoid Leukaemia	10 (66.7)	3 (20.0)	2 (13.3)	0	0	0	15 (2.4)
Lymphoma	8 (40.0)	0	5 (25.0)	7 (35.0)	0	0	20 (3.1)
Melanoma	33 (67.3)	9 (18.4)	4 (8.2)	0	0	3 (6.1)	49 (7.7)
Myeloid Neoplasm	0	0	0	0	18 (100)	0	18 (2.8)
Oesophagus	0	1 (14.3)	3 (42.9)	3 (42.9)	0	0	7 (1.1)
Ovary	2 (25.0)	0	5 (62.5)	1 (12.5)	0	0	8 (1.3)
Pancreas	3 (20.0)	2 (13.3)	2 (13.3)	8 (53.3)	0	0	15 (2.4)
Plasma Cell Neoplasm	2 (20.0)	2 (20.0)	0	0	0	6 (60.0)	10 (1.6)
Prostate	124 (52.1)	46 (19.3)	55 (23.1)	9 (3.8)	0	4 (1.7)	238 (37.5)
Sarcoma	1 (14.3)	1 (14.3)	4 (57.1)	0	0	1 (14.3)	7 (1.1)
Stomach	0	1 (20.0)	1 (20.0)	3 (60.0)	0	0	5 (0.8)
Thyroid	4 (100)	0	0	0	0	0	4 (0.6)
Urothelial Tract	0	0	2 (100)	0	0	0	2 (0.3)
Uterus	26 (96.3)	0	1 (3.7)	0	0	0	27 (4.3)
Other[e]	4 (13.3)	1 (3.3)	8 (26.7)	1 (3.3)	14 (46.7)	2 (6.7)	30 (4.7)
NCRAS Cancer of	0	0	0	0	2 (100)	0	2 (0.3)

Cancer type diagnosed	Stage,[a] n (%)						Total, n (%)
	I	II	III	IV	Unstageable[b]	Missing	
Unknown Primary[d]							
**Second screening round**							
**Participants with positive MCED test results and cancer diagnosis**							
Total	56 (21.5)	49 (18.8)	75 (28.7)	65 (24.9)	2 (0.8)	14 (5.4)	261
Anus	2 (66.7)	0	1 (33.3)	0	0	0	3 (1.1)
Bladder	2 (66.7)	0	1 (33.3)	0	0	0	3 (1.1)
Breast	1 (6.7)	10 (66.7)	3 (20.0)	1 (6.7)	0	0	15 (5.7)
Cervix	1 (100)	0	0	0	0	0	1 (0.4)
Colon/Rectum	20 (35.7)	14 (25.0)	16 (28.6)	6 (10.7)	0	0	56 (21.5)
Gallbladder	0	1 (33.3)	0	2 (66.7)	0	0	3 (1.1)
Head and Neck	5 (62.5)	2 (25.0)	1 (12.5)	0	0	0	8 (3.1)
Kidney	0	0	1 (100)	0	0	0	1 (0.4)
Liver/Bile-duct	2 (25.0)	3 (37.5)	3 (37.5)	0	0	0	8 (3.1)
Lung	5 (13.5)	4 (10.8)	16 (43.2)	11 (29.7)	0	1 (2.7)	37 (14.2)
Lymphoid Leukaemia	5 (71.4)	1 (14.3)	0	0	0	1 (14.3)	7 (2.7)
Lymphoma	0	4 (21.1)	5 (26.3)	9 (47.4)	0	1 (5.3)	19 (7.3)

Cancer type diagnosed	Stage,[a] n (%)						Total, n (%)
	I	II	III	IV	Unstageable[b]	Missing	
Melanoma	0	0	0	1 (100)	0	0	1 (0.4)
Oesophagus	1 (10.0)	3 (30.0)	4 (40.0)	2 (20.0)	0	0	10 (3.8)
Ovary	1 (16.7)	0	2 (33.3)	3 (50.0)	0	0	6 (2.3)
Pancreas	0	1 (10.0)	2 (20.0)	7 (70.0)	0	0	10 (3.8)
Plasma Cell Neoplasm	0	1 (10.0)	0	0	0	9 (90.0)	10 (3.8)
Prostate	3 (8.6)	4 (11.4)	11 (31.4)	16 (45.7)	0	1 (2.9)	35 (13.4)
Sarcoma	0	0	0	0	0	1 (100)	1 (0.4)
Stomach	1 (16.7)	0	2 (33.3)	3 (50.0)	0	0	6 (2.3)
Urothelial Tract	0	0	1 (33.3)	2 (66.7)	0	0	3 (1.1)
Uterus	3 (50.0)	1 (16.7)	2 (33.3)	0	0	0	6 (2.3)
Other[f]	2 (25.0)	0	4 (50.0)	2 (25.0)	0	0	8 (3.1)
Multiple cancers	2 (100)	0	0	0	0	0	2 (0.8)
NCRAS Cancer of Unknown Primary[d]	0	0	0	0	2 (100)	0	2 (0.8)
**Participants with negative MCED test results whose cancer diagnosis was triggered by NHS cancer screening**							
Total	33 (58.9)	18 (32.1)	3 (5.4)	1 (1.8)	0	1 (1.8)	56

Cancer type diagnosed	Stage,[a] n (%)						Total, n (%)
	I	II	III	IV	Unstageable[b]	Missing	
Breast	24 (58.5)	15 (36.6)	1 (2.4)	0	0	1 (2.4)	41 (73.2)
Cervix	0	1 (100)	0	0	0	0	1 (1.8)
Colon/Rectum	7 (63.6)	2 (18.2)	1 (9.1)	1 (9.1)	0	0	11 (19.6)
Lung	2 (66.7)	0	1 (33.3)	0	0	0	3 (5.4)
**Participants with negative MCED test results whose cancer diagnosis was not triggered by NHS cancer screening**							
Total[g]	304 (47.1)	107 (16.6)	137 (21.2)	50 (7.7)	36 (5.6)	11 (1.7)	646
Anus	1 (100)	0	0	0	0	0	1 (0.2)
Bladder	7 (43.8)	3 (18.8)	5 (31.2)	0	0	1 (6.2)	16 (2.5)
Breast	24 (42.9)	27 (48.2)	2 (3.6)	3 (5.4)	0	0	56 (8.7)
Colon/Rectum	16 (42.1)	6 (15.8)	12 (31.6)	4 (10.5)	0	0	38 (5.9)
Gallbladder	0	1 (100)	0	0	0	0	1 (0.2)
Head and Neck	5 (33.3)	2 (13.3)	4 (26.7)	4 (26.7)	0	0	15 (2.3)
Kidney	9 (47.4)	3 (15.8)	3 (15.8)	3 (15.8)	0	1 (5.3)	19 (2.9)
Liver/Bile-duct	0	0	0	1 (100)	0	0	1 (0.2)
Lung	31 (58.5)	2 (3.8)	7 (13.2)	13 (24.5)	0	0	53 (8.2)
Lymphoid Leukaemia	7 (53.8)	0	0	0	4 (30.8)	2 (15.4)	13 (2.0)

Cancer type diagnosed	Stage,[a] n (%)						Total, n (%)
	I	II	III	IV	Unstageable[b]	Missing	
Lymphoma	6 (30.0)	1 (5.0)	7 (35.0)	5 (25.0)	0	1 (5.0)	20 (3.1)
Melanoma	30 (63.8)	11 (23.4)	6 (12.8)	0	0	0	47 (7.3)
Myeloid Neoplasm	0	0	0	0	16 (100)	0	16 (2.5)
Oesophagus	1 (20.0)	2 (40.0)	2 (40.0)	0	0	0	5 (0.8)
Ovary	2 (28.6)	0	5 (71.4)	0	0	0	7 (1.1)
Pancreas	2 (20.0)	3 (30.0)	0	5 (50.0)	0	0	10 (1.5)
Plasma Cell Neoplasm	3 (37.5)	1 (12.5)	0	0	0	4 (50.0)	8 (1.2)
Prostate	121 (49.6)	38 (15.6)	75 (30.7)	8 (3.3)	0	2 (0.8)	244 (37.8)
Sarcoma	3 (75.0)	1 (25.0)	0	0	0	0	4 (0.6)
Stomach	1 (25.0)	2 (50.0)	0	1 (25.0)	0	0	4 (0.6)
Thyroid	3 (42.9)	1 (14.3)	0	3 (42.9)	0	0	7 (1.1)
Urothelial Tract	3 (50.0)	1 (16.7)	2 (33.3)	0	0	0	6 (0.9)
Uterus	17 (81.0)	1 (4.8)	3 (14.3)	0	0	0	21 (3.3)
Other[g,h]	11 (36.7)	1 (3.3)	4 (13.3)	0	13 (43.3)	0	30 (4.6)
Multiple cancers	1 (100)	0	0	0	0	0	1 (0.2)
NCRAS	0	0	0	0	3 (100)	0	3 (0.5)

Cancer type diagnosed	Stage,[a] n (%)						Total, n (%)
	I	II	III	IV	Unstageable[b]	Missing	
Cancer of Unknown Primary[d]							
**Third screening round**							
**Participants with positive MCED test results and cancer diagnosis**							
Total	58 (22.6)	37 (14.4)	76 (29.6)	64 (24.9)	4 (1.6)	18 (7.0)	257
Anus	1 (100)	0	0	0	0	0	1 (0.4)
Bladder	2 (50.0)	2 (50.0)	0	0	0	0	4 (1.6)
Breast	3 (18.8)	6 (37.5)	3 (18.8)	4 (25.0)	0	0	16 (6.2)
Colon/Rectum	15 (28.8)	9 (17.3)	22 (42.3)	6 (11.5)	0	0	52 (20.2)
Gallbladder	0	1 (14.3)	2 (28.6)	4 (57.1)	0	0	7 (2.7)
Head and Neck	7 (63.6)	0	2 (18.2)	2 (18.2)	0	0	11 (4.3)
Kidney	1 (25.0)	0	2 (50.0)	0	0	1 (25.0)	4 (1.6)
Liver/Bile-duct	5 (38.5)	4 (30.8)	2 (15.4)	1 (7.7)	0	1 (7.7)	13 (5.1)
Lung	4 (9.5)	6 (14.3)	20 (47.6)	12 (28.6)	0	0	42 (16.3)
Lymphoid Leukaemia	4 (80.0)	0	0	0	1 (20.0)	0	5 (1.9)
Lymphoma	5 (25.0)	2 (10.0)	3 (15.0)	10 (50.0)	0	0	20 (7.8)

Cancer type diagnosed	Stage,[a] n (%)						Total, n (%)
	I	II	III	IV	Unstageable[b]	Missing	
Melanoma	1 (50.0)	0	0	1 (50.0)	0	0	2 (0.8)
Myeloid Neoplasm	0	0	0	0	1 (100)	0	1 (0.4)
Oesophagus	2 (15.4)	4 (30.8)	1 (7.7)	4 (30.8)	0	2 (15.4)	13 (5.1)
Ovary	3 (30.0)	1 (10.0)	2 (20.0)	4 (40.0)	0	0	10 (3.9)
Pancreas	0	1 (12.5)	2 (25.0)	5 (62.5)	0	0	8 (3.1)
Plasma Cell Neoplasm	0	0	0	0	0	13 (100)	13 (5.1)
Prostate	2 (9.1)	1 (4.5)	10 (45.5)	8 (36.4)	1 (4.5)	0	22 (8.6)
Stomach	0	0	3 (75.0)	1 (25.0)	0	0	4 (1.6)
Uterus	2 (40.0)	0	1 (20.0)	2 (40.0)	0	0	5 (1.9)
Other[i]	1 (33.3)	0	1 (33.3)	0	0	1 (33.3)	3 (1.2)
NCRAS Cancer of Unknown Primary[d]	0	0	0	0	1 (100)	0	1 (0.4)
**Participants with negative MCED test results whose cancer diagnosis was triggered by NHS cancer screening**							
Total	38 (61.3)	17 (27.4)	6 (9.7)	1 (1.6)	0	0	62
Breast	27 (62.8)	14 (32.6)	2 (4.7)	0	0	0	43 (69.4)
Cervix	1 (100)	0	0	0	0	0	1 (1.6)

Cancer type diagnosed	Stage,[a] n (%)						Total, n (%)
	I	II	III	IV	Unstageable[b]	Missing	
Colon/Rectum	5 (45.5)	3 (27.3)	3 (27.3)	0	0	0	11 (17.7)
Lung	5 (71.4)	0	1 (14.3)	1 (14.3)	0	0	7 (11.3)
**Participants with negative MCED test results whose cancer diagnosis was not triggered by NHS cancer screening**							
Total	292 (45.5)	126 (19.6)	106 (16.5)	59 (9.2)	39 (6.1)	20 (3.1)	642
Bladder	11 (78.6)	2 (14.3)	0	1 (7.1)	0	0	14 (2.2)
Breast	22 (42.3)	28 (53.8)	1 (1.9)	1 (1.9)	0	0	52 (8.1)
Colon/Rectum	16 (57.1)	4 (14.3)	4 (14.3)	3 (10.7)	0	1 (3.6)	28 (4.4)
Gallbladder	0	1 (33.3)	0	2 (66.7)	0	0	3 (0.5)
Head and Neck	4 (36.4)	3 (27.3)	2 (18.2)	2 (18.2)	0	0	11 (1.7)
Kidney	9 (60.0)	0	5 (33.3)	1 (6.7)	0	0	15 (2.3)
Lung	25 (55.6)	4 (8.9)	8 (17.8)	8 (17.8)	0	0	45 (7.0)
Lymphoid Leukaemia	3 (50.0)	1 (16.7)	0	0	1 (16.7)	1 (16.7)	6 (0.9)
Lymphoma	4 (17.4)	4 (17.4)	5 (21.7)	10 (43.5)	0	0	23 (3.6)
Melanoma	42 (71.2)	14 (23.7)	3 (5.1)	0	0	0	59 (9.2)
Myeloid Neoplasm	0	0	0	0	27 (100)	0	27 (4.2)
Oesophagus	2 (20.0)	2 (20.0)	3 (30.0)	1 (10.0)	0	2 (20.0)	10 (1.6)

Cancer type diagnosed	Stage,[a] n (%)						Total, n (%)
	I	II	III	IV	Unstageable[b]	Missing	
Ovary	2 (28.6)	1 (14.3)	3 (42.9)	1 (14.3)	0	0	7 (1.1)
Pancreas	2 (11.8)	4 (23.5)	3 (17.6)	8 (47.1)	0	0	17 (2.6)
Plasma Cell Neoplasm	1 (9.1)	1 (9.1)	0	0	0	9 (81.8)	11 (1.7)
Prostate	119 (49.8)	50 (20.9)	57 (23.8)	13 (5.4)	0	0	239 (37.2)
Sarcoma	3 (30.0)	1 (10.0)	4 (40.0)	0	0	2 (20.0)	10 (1.6)
Stomach	0	0	0	1 (100)	0	0	1 (0.2)
Thyroid	5 (62.5)	3 (37.5)	0	0	0	0	8 (1.2)
Urothelial Tract	0	1 (25.0)	1 (25.0)	1 (25.0)	0	1 (25.0)	4 (0.6)
Uterus	13 (76.5)	0	1 (5.9)	2 (11.8)	0	1 (5.9)	17 (2.6)
Other[j]	9 (28.1)	2 (6.2)	5 (15.6)	4 (12.5)	9 (28.1)	3 (9.4)	32 (5.0)
Multiple cancers	0	0	1 (100)	0	0	0	1 (0.2)
NCRAS Cancer of Unknown Primary[d]	0	0	0	0	2 (100)	0	2 (0.3)

For participants with multiple cancer diagnoses, cancer type and stage were based on the earliest diagnosed cancer only.

[a]The cancer type and stage are based on the earliest diagnosed cancer if a participant had multiple cancers. The stage distribution is calculated based on the total number of cancers per cancer type.

[b]Unstageable = no valid staging system exists for the combination of topo/morpho codes. Binet stages are mapped as follows: A=I, B=II, C=III; Chang stages are mapped as follows: M0=I, M1=II, M2/3=III, M4=IV.

[c]Other cancer types in this group include brain (n=1), germ cell (n=1), mesothelioma (n=1), Vulva (n=1).

[d]NCRAS Cancer of Unknown Primary is defined as an invasive cancer with ICD-O-3 behaviour code 6 or 9; or an invasive cancer with behaviour code 3, topographical code C809R and unmapped cancer label.

[e]Other cancer types in this group include brain (n=12), mesothelioma (n=6), small intestine (n = 4), vulva (n=3), ampulla of Vater (n = 1), eyelid (n=1), penis (n=1), thymus (n=1), trachea (n=1).

[f]Other cancer types in this group include small intestine (n=4), mesothelioma (n=2), germ cell (n=1), penis (n=1).

[g]One stage 0 cancer was diagnosed and included in totals but not shown here. Sums of the columns for this row are therefore less than the total.

[h]Other cancer types in this group include brain (n=11), mesothelioma (n=7), vulva (n=3), germ cell (n=2), small intestine (n=2), thymus (n=2), carotid body (n=1), skin cancer (n=1), vagina (n=1).

[i]Other cancer types in this group include mesothelioma (n=1), vagina (n=1), vulva (n=1).

[j]Other cancer types in this group include brain (n=8), mesothelioma (n=7), small intestine (n=6), skin cancer (n=3), adrenal (n=2), ampulla of Vater (n=2), penis (n=2), vulva (n=2).

MCED, multi-cancer early detection; NCRAS, National Cancer Registration and Analysis Service; NHS, National Health Service.

Supplementary Table S11. Distribution of MCED-Detected Cancers by AJCC Cancer Types.

**Cancer type diagnosed**	**First screening round,** n (%) (n[a]=439)	**Second screening round,** n (%) (n[a]=275)	**Third screening round,** n (%) (n[a]=265)	**Aggregate,** n (%) (n[a]=979)
Anus	3 (0.7)	3 (1.1)	1 (0.4)	7 (0.7)
Appendix Carcinoma	2 (0.5)	1 (0.4)	0	3 (0.3)
Brain and Spinal Cord	1 (0.2)	0	0	1 (0.1)
Breast	29 (6.6)	17 (6.2)	17 (6.4)	63 (6.4)
Cervix Uteri	1 (0.2)	1 (0.4)	0	2 (0.2)
Colon and Rectum	86 (19.6)	57 (20.7)	53 (20.0)	196 (20.0)
Corpus Uteri - Carcinoma and Carcinosarcoma	7 (1.6)	6 (2.2)	6 (2.3)	19 (1.9)
Distal Bile Duct	1 (0.2)	0	0	1 (0.1)
Exocrine Pancreas	19 (4.3)	10 (3.6)	8 (3.0)	37 (3.8)
Gallbladder	2 (0.5)	3 (1.1)	5 (1.9)	10 (1.0)
Hodgkin and Non-Hodgkin Lymphomas	45 (10.3)	26 (9.5)	23 (8.7)	94 (9.6)
HPV-Mediated (p16+) Oropharyngeal Cancer	15 (3.4)	6 (2.2)	9 (3.4)	30 (3.1)
Intrahepatic Bile Ducts	6 (1.4)	4 (1.5)	2 (0.8)	12 (1.2)
Kidney	10 (2.3)	1 (0.4)	4 (1.5)	15 (1.5)
Larynx	1 (0.2)	0	0	1 (0.1)
Leukaemia	1 (0.2)	0	2 (0.8)	3 (0.3)
Liver	7 (1.6)	5 (1.8)	11 (4.2)	23 (2.3)
Lung	57 (13.0)	37 (13.5)	42 (15.8)	136 (13.9)
Major Salivary Glands	0	1 (0.4)	0	1 (0.1)
Malignant Pleural Mesothelioma	1 (0.2)	2 (0.7)	1 (0.4)	4 (0.4)
Melanoma of the Skin	3 (0.7)	2 (0.7)	3 (1.1)	8 (0.8)

**Cancer type diagnosed**	**First screening round,** n (%) (n[a]=439)	**Second screening round,** n (%) (n[a]=275)	**Third screening round,** n (%) (n[a]=265)	**Aggregate,** n (%) (n[a]=979)
Neuroendocrine Tumours of the Jejunum and Ileum	0	1 (0.4)	0	1 (0.1)
Neuroendocrine Tumours of the Pancreas	1 (0.2)	0	0	1 (0.1)
Oesophagus and Oesophagogastric Junction	16 (3.6)	8 (2.9)	10 (3.8)	34 (3.5)
Oral Cavity	1 (0.2)	0	0	1 (0.1)
Oropharynx (p16-) and Hypopharynx	0	2 (0.7)	2 (0.8)	4 (0.4)
Ovary, Fallopian Tube, and Primary Peritoneal Carcinoma	19 (4.3)	6 (2.2)	10 (3.8)	35 (3.6)
Penis	0	1 (0.4)	0	1 (0.1)
Perihilar Bile Ducts	2 (0.5)	0	2 (0.8)	4 (0.4)
Plasma Cell Myeloma and Plasma Cell Disorders	24 (5.5)	10 (3.6)	14 (5.3)	48 (4.9)
Prostate	52 (11.8)	38 (13.8)	24 (9.1)	114 (11.6)
Renal Pelvis and Ureter	1 (0.2)	5 (1.8)	0	6 (0.6)
Small Intestine	0	3 (1.1)	0	3 (0.3)
Soft Tissue Sarcoma - Unusual Histologies and Sites	1 (0.2)	0	0	1 (0.1)
Soft Tissue Sarcoma of the Abdomen and Thoracic Visceral Organs	1 (0.2)	0	0	1 (0.1)
Stomach	8 (1.8)	5 (1.8)	2 (0.8)	15 (1.5)
Testis	1 (0.2)	1 (0.4)	1 (0.4)	3 (0.3)

**Cancer type diagnosed**	**First screening round,** n (%) (n[a]=439)	**Second screening round,** n (%) (n[a]=275)	**Third screening round,** n (%) (n[a]=265)	**Aggregate,** n (%) (n[a]=979)
Thyroid - Differentiated and Anaplastic Carcinoma	1 (0.2)	1 (0.4)	1 (0.4)	3 (0.3)
Urinary Bladder	3 (0.7)	4 (1.5)	4 (1.5)	11 (1.1)
Vagina	0	0	1 (0.4)	1 (0.1)
Vulva	1 (0.2)	0	1 (0.4)	2 (0.2)
Cancer of Unknown Primary[b]	3 (0.7)	3 (1.1)	1 (0.4)	7 (0.7)
Other/Unknown	2 (0.5)	2 (0.7)	0	4 (0.4)
Missing	5 (1.1)	3 (1.1)	5 (1.9)	13 (1.3)

[a]n represents individual cancers, not participants; participants with multiple cancers may appear in multiple rows.

[b]Cancer of Unknown Primary is defined as an invasive cancer with ICD-O-3 behaviour code 6 or 9; or an invasive cancer with behaviour code 3, topographical code C809 and unmapped cancer label.

AJCC, American Joint Committee on Cancer; MCED, multi-cancer early detection.

Supplementary Table S12. Distribution of Stage for Cancers per First-Cancer Diagnosed.

	**Positive MCED test result, cancer diagnosis,** n (%)	**Negative MCED test result, cancer diagnosis,** n (%)	**Total,** n (%)
**First screening round**			
Total, N	419	708	1127
Stage I	88 (21.0)	345 (48.7)	433 (38.4)
Stage II	78 (18.6)	133 (18.8)	211 (18.7)
Stage III	123 (29.4)	122 (17.2)	245 (21.7)
Stage IV	103 (24.6)	56 (7.9)	159 (14.1)
Stage I-II	166 (39.6)	478 (67.5)	644 (57.1)
Stage I-III	289 (69.0)	600 (84.7)	889 (78.9)
Unstageable[a]	4 (1.0)	34 (4.8)	38 (3.4)
Missing	23 (5.5)	18 (2.5)	41 (3.6)
**Second screening round**			
Total, N	261	702	963
Stage 0	0	1 (0.1)	1 (0.1)
Stage I	56 (21.5)	337 (48.0)	393 (40.8)
Stage II	49 (18.8)	125 (17.8)	174 (18.1)
Stage III	75 (28.7)	140 (19.9)	215 (22.3)
Stage IV	65 (24.9)	51 (7.3)	116 (12.0)
Stage I-II	105 (40.2)	462 (65.8)	567 (58.9)
Stage I-III	180 (69.0)	602 (85.8)	782 (81.2)
Unstageable[a]	2 (0.8)	36 (5.1)	38 (3.9)
Missing	14 (5.4)	12 (1.7)	26 (2.7)
**Third screening round**			
Total, N	257	704	961
Stage I	58 (22.6)	330 (46.9)	388 (40.4)

	**Positive MCED test result, cancer diagnosis,** n (%)	**Negative MCED test result, cancer diagnosis,** n (%)	**Total,** n (%)
Stage II	37 (14.4)	143 (20.3)	180 (18.7)
Stage III	76 (29.6)	112 (15.9)	188 (19.6)
Stage IV	64 (24.9)	60 (8.5)	124 (12.9)
Stage I-II	95 (37.0)	473 (67.2)	568 (59.1)
Stage I-III	171 (66.5)	585 (83.1)	756 (78.7)
Unstageable[a]	4 (1.6)	39 (5.5)	43 (4.5)
Missing	18 (7.0)	20 (2.8)	38 (4.0)

[a]Unstageable = no valid staging system exists for the combination of topo/morpho codes. Binet stages are mapped as follows: A=I, B=II, C=III; Chang stages are mapped as follows: M0=I, M1=II, M2/3=III, M4=IV.

Supplementary Table S13. Sensitivity Analysis for PPV by Screening Round and in Aggregate Accounting for Additionally Identified Recurrent Cancers.

**Screening round**	**Number of positive tests,** n	**Number of participants with true-positive results accounting for only primary cancer,** n	**Number of participants with true-positive results accounting for additionally identified recurrent cancer,** n	**PPV for cancer signal detection accounting for additionally identified recurrent cancer,** % (95% CI)
**All test analysable participants**				
First	722	419	436	60.4 (56.8, 63.9)
Second	518	261	268	51.7 (47.4, 56.0)
Third	561	257	269	48.0 (43.8, 52.1)
Aggregate	1801	937	973	54.0 (51.7, 56.3)
**Participants with a prior cancer history**				
First	96	35	52	54.2 (44.2, 63.8)
Second	74	32	39	52.7 (41.5, 63.7)
Third	62	14	26	41.9 (30.5, 54.3)
Aggregate	232	81	117	50.4 (44.0, 56.8)

PPV, positive predictive value.

Supplementary Table S14. Exploratory Analysis for Participants With False-positive Results with Additional Follow-up.

	Period during which cancer was diagnosed		
	**Second screening round follow-up period**	**Third screening round follow-up period**	**Combined second and third round follow-up period**
**Number of false-positive participants in first screening round (n=303)**			
Participants later diagnosed with cancer, n (%)	36 (11.9)	18 (5.9)	54 (17.8)
**CSO concordance (for participants with cancer diagnosed, n=18)**			
Participants with correct CSO1/2, n (%)	29 (80.6)	15 (83.3)	44 (81.5)
Participants with correct CSO1, n (%)	27 (75.0)	14 (77.8)	41 (75.9)
**Number of false-positive participants in second screening round (n=257)**			
Participants later diagnosed with cancer, n (%)	NA	25 (9.7)	
**CSO concordance (for participants with cancer diagnosed, n=25)**			
Participants with correct CSO1/2, n (%)	NA	18 (72.0)	
Participants with correct CSO1, n (%)	NA	17 (68.0)	

CSO, cancer signal origin; NA, not applicable.

Supplementary Table S15. Definitions of Screening Performance Metrics.

Term	Definition
Participant with a false-negative result	Participant who receives a “no cancer signal detected” (negative) test result and a cancer diagnosis
Participant with a false-positive result	Participant who receives a “cancer signal detected” (positive) test result and no cancer diagnosis
Participant with a true-negative result	Participant who receives a “no cancer signal detected” (negative) test result and no cancer diagnosis
Participant with a true-positive result	Participant who receives a “cancer signal detected” (positive) test result and cancer diagnosis

Metric	Calculations	
	**Numerator**	**Denominator**
Cancer detection rate	Number of participants with a true-positive result within a given screening round	Total number of participants with an MCED test result within that screening round
Episode sensitivity	Number of participants with a true-positive result within a given screening round	Sum of participants with true-positive results and participants with false-negative results within that screening round
False positive rate	Number of participants with a false-positive result within a given screening round	Sum of participants with true-negative results and participants with false-positive results within that screening round
Negative likelihood ratio	The probability that a participant with a cancer diagnosis has a negative test result within a given screening round	The probability that a participant with no cancer diagnosis has a negative test result within that screening round
Negative predictive value (NPV)	Number of participants with true-negative results within a given screening round	Sum of participants with true-negative results and participants with false-negative results within that screening round
Positive likelihood ratio	The probability that a participant with cancer	The probability that a participant with no cancer

	diagnosis has a positive test result within a given screening round	diagnosis has a positive test result within that screening round
Positive predictive value (PPV)	Number of participants with true-positive results within a given screening round	Sum of participants with true-positive results and participants with false-positive results within that screening round
Specificity	Number of participants with true-negative results within a given screening round	Sum of participants with true-negative results and participants with false-positive results within that screening round

Other metrics have been defined elsewhere. Please refer to Nabavizadeh et al.[1]

1. Nabavizadeh, N. *et al.* Performance and Safety of a Multi-Cancer Early Detection Test: the PATHFINDER 2 Study. *Nat. Med.* (2026).

Figures

Supplementary Figure S1. Aggregate Episode Sensitivity by Cancer Type Diagnosed.

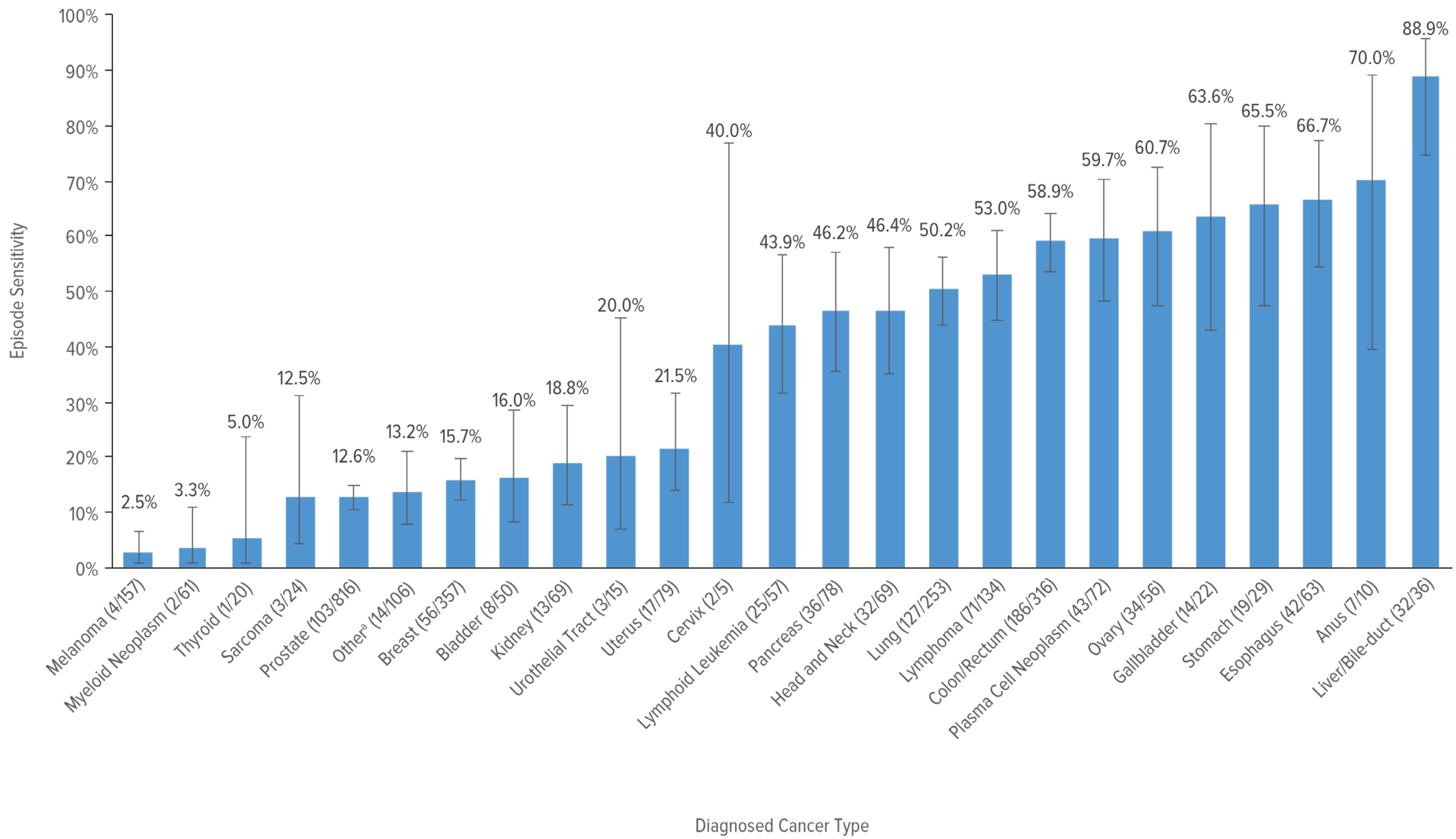

Episode sensitivity data are presented as observed percentages, calculated as defined in **Supplementary Table S15**; error bars denote two-sided 95% CIs. Cancer types with less than 5 cases are not shown.

[a]Other cancer types include brain (n=32), mesothelioma (n=24), small intestine (n=16), vulva (n=10), penis (n=4), skin cancer (n=4), ampulla of Vater (n=3), germ cell (n=3), thymus (n=3), adrenal (n=2), vagina (n=2), carotid body (n=1), eyelid (n=1), trachea (n=1).

Supplementary Figure S2. Confusion Matrix of Predicted CSO vs Clinical CSO by Sex by Round and in Aggregate.

First Screening Round
Female

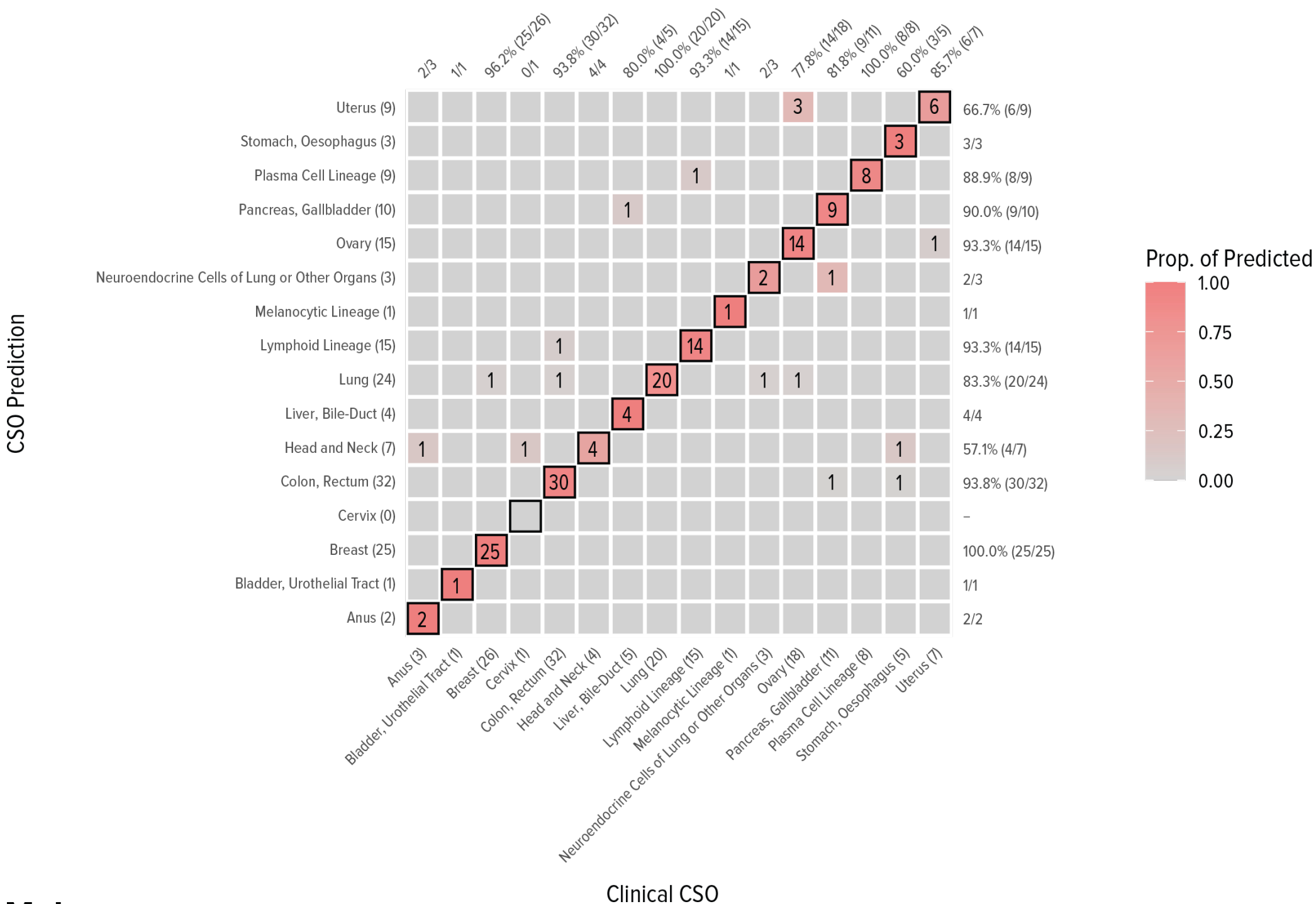

Male

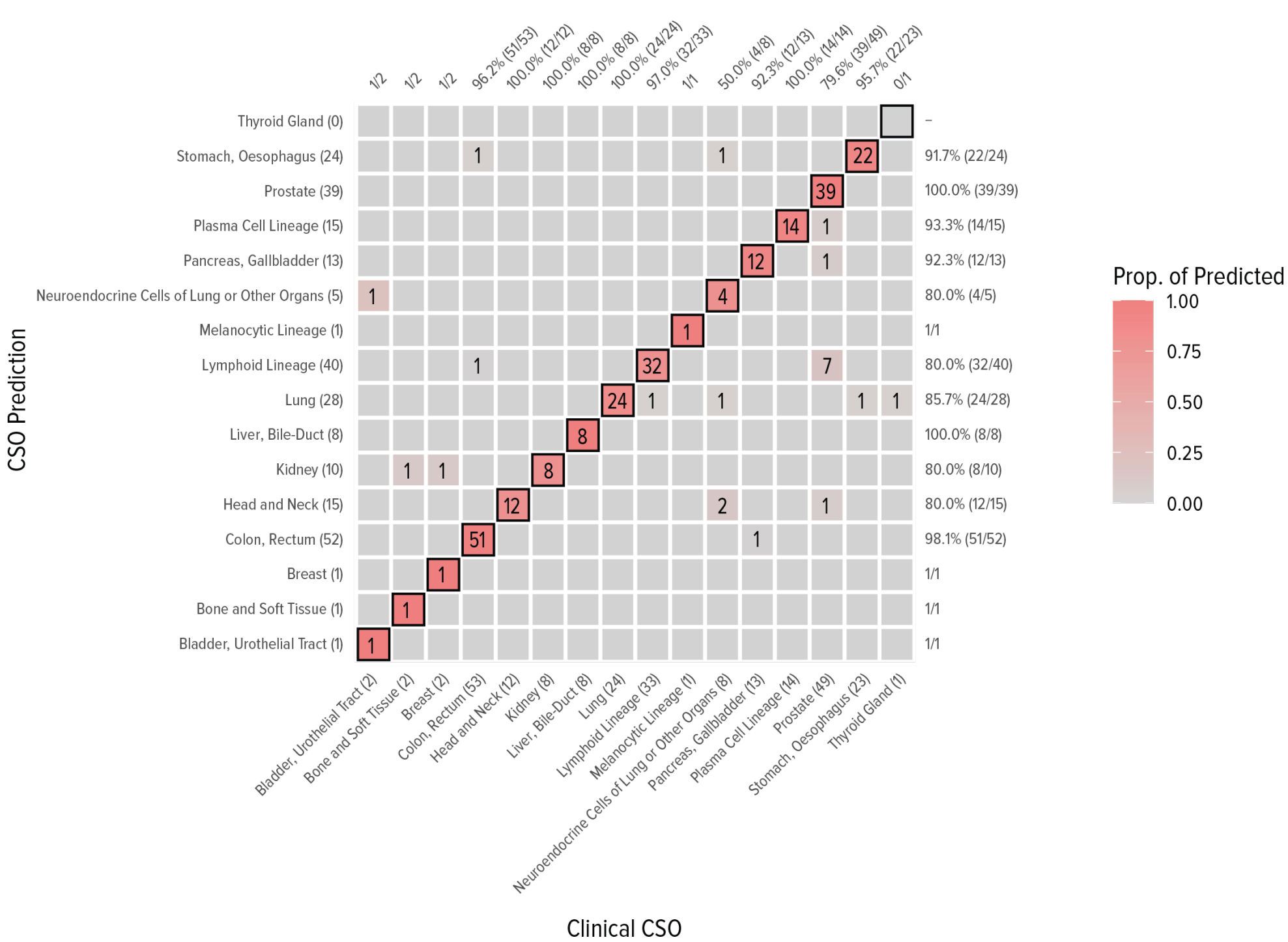

Second Screening Round
Female

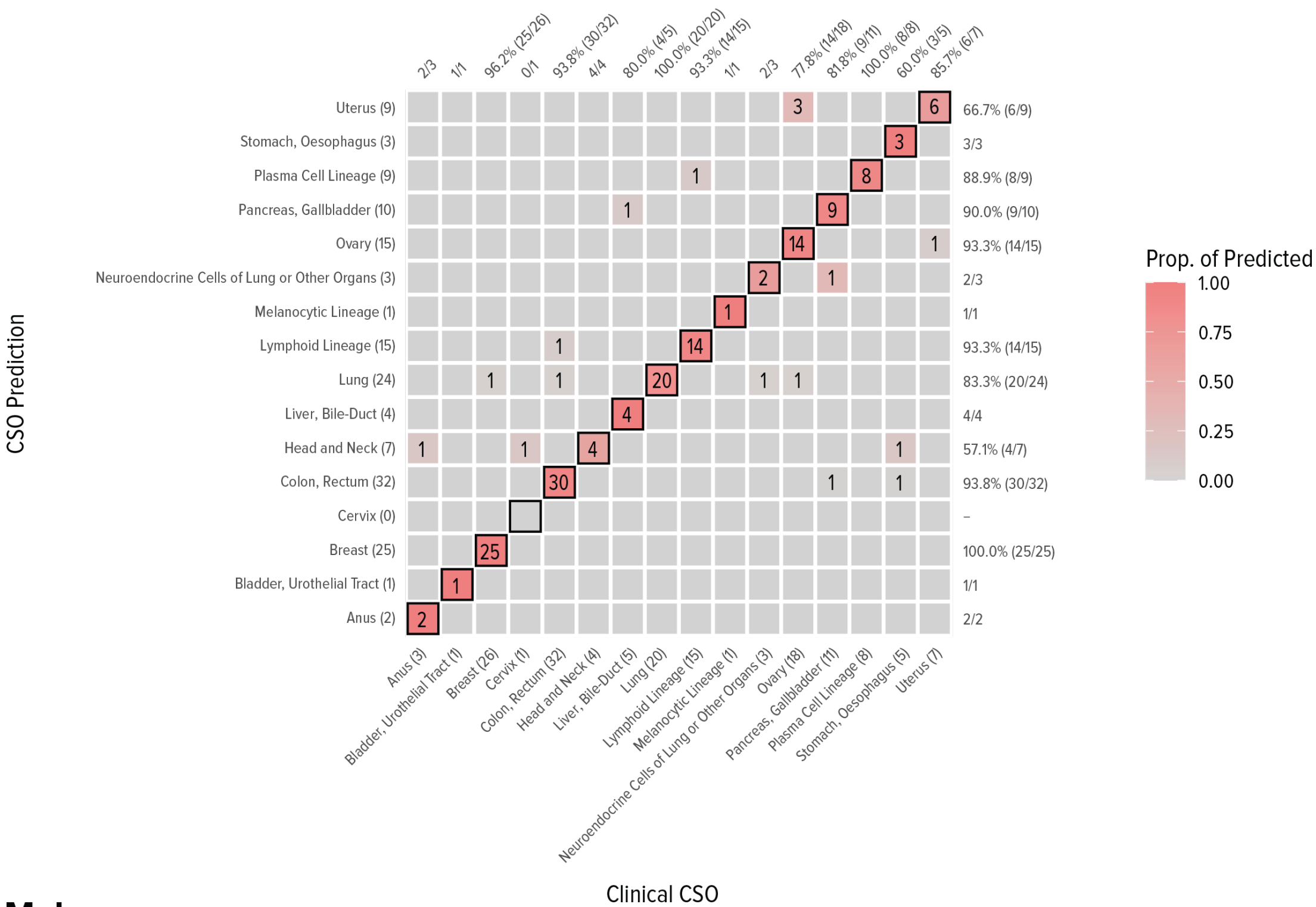

Male

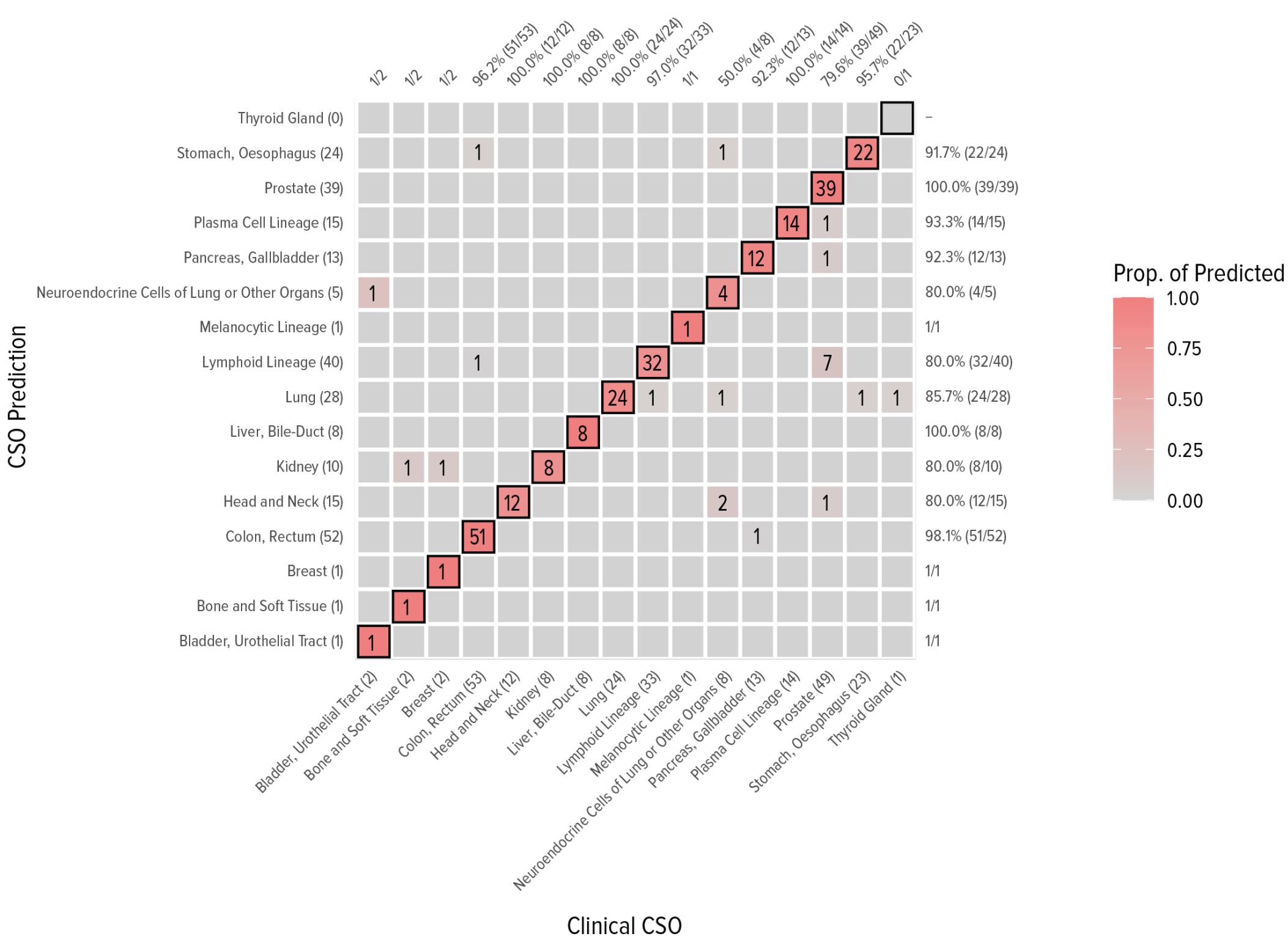

Third Screening Round

Female

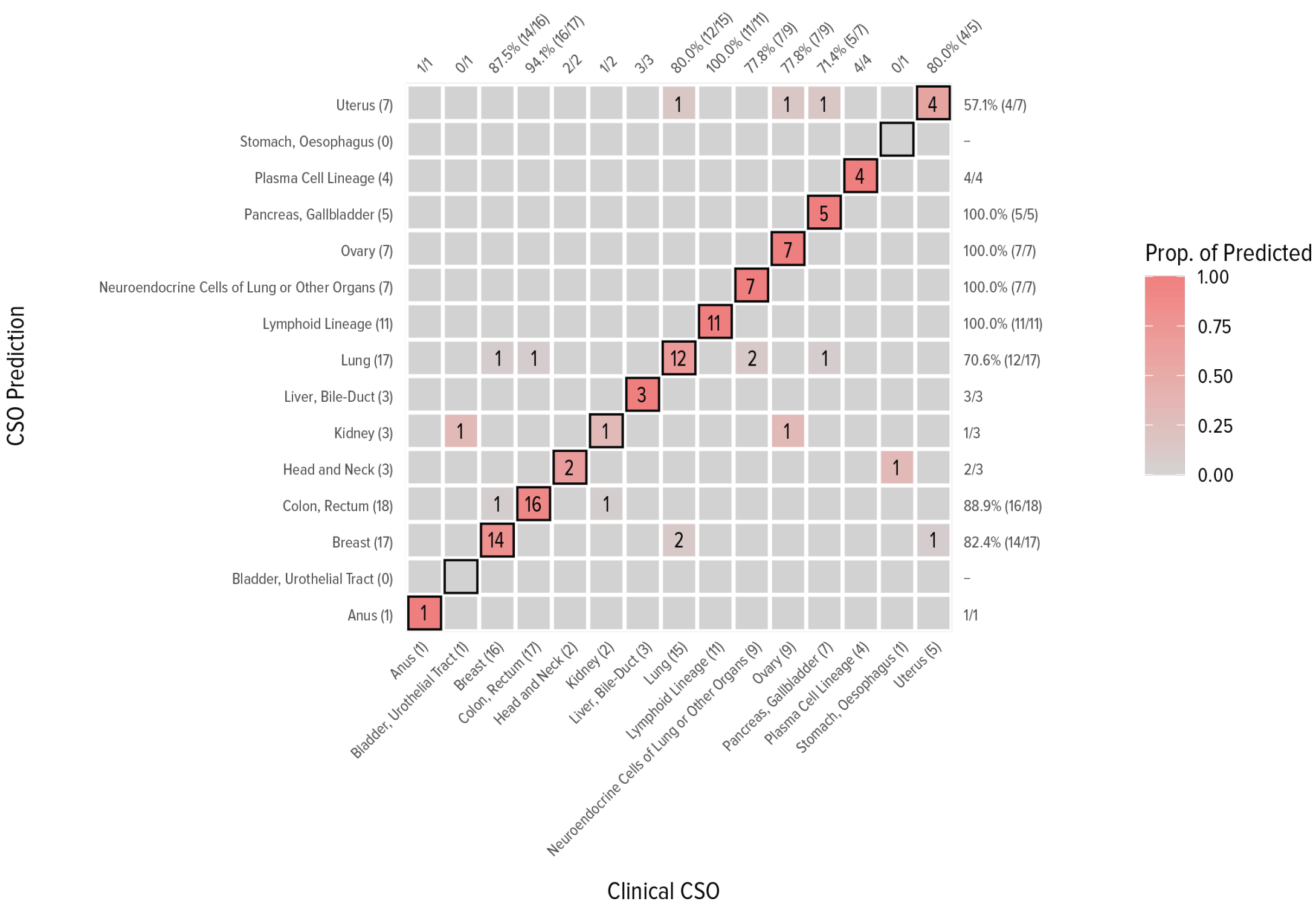

Male

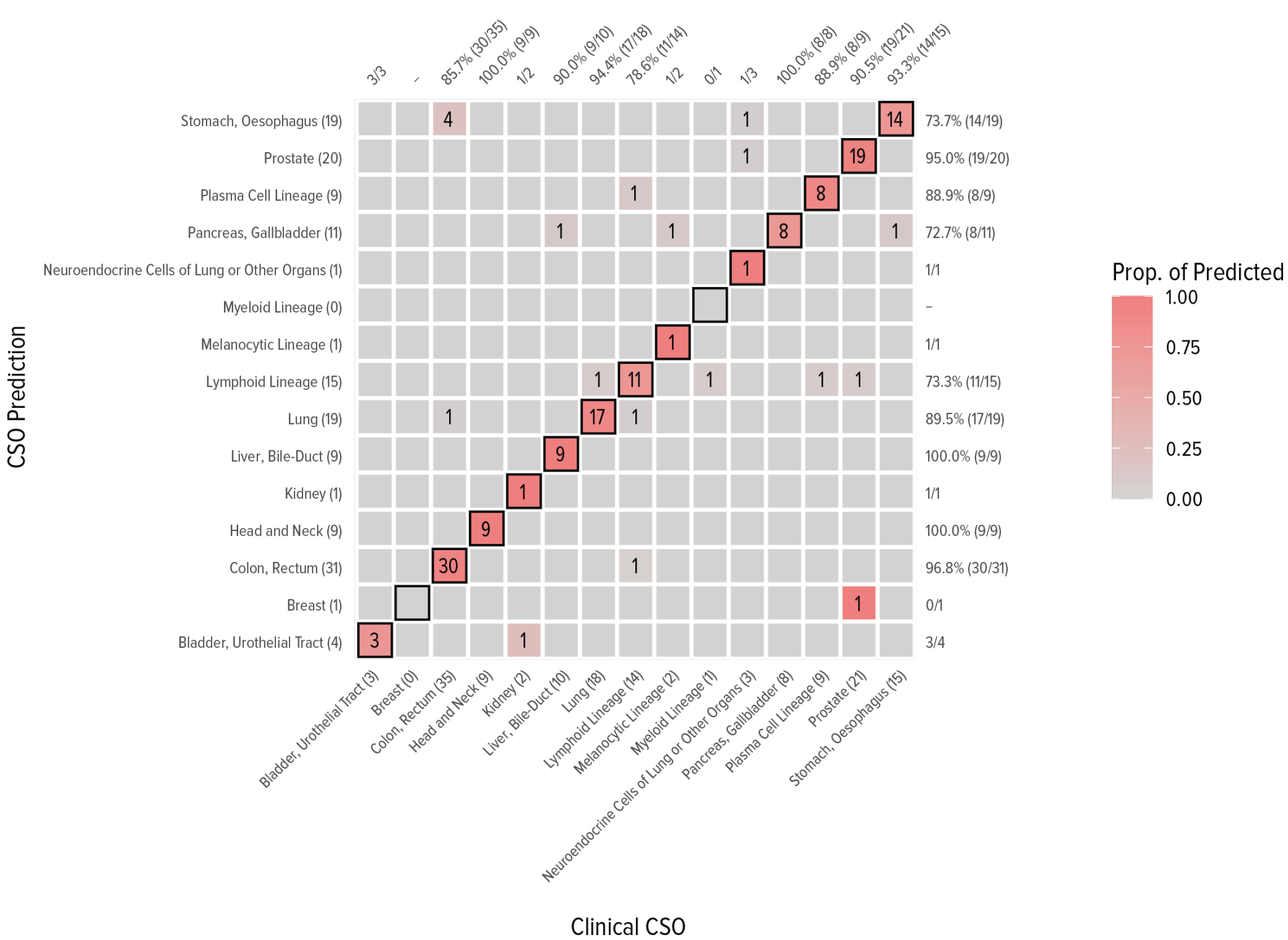

Aggregate

Female

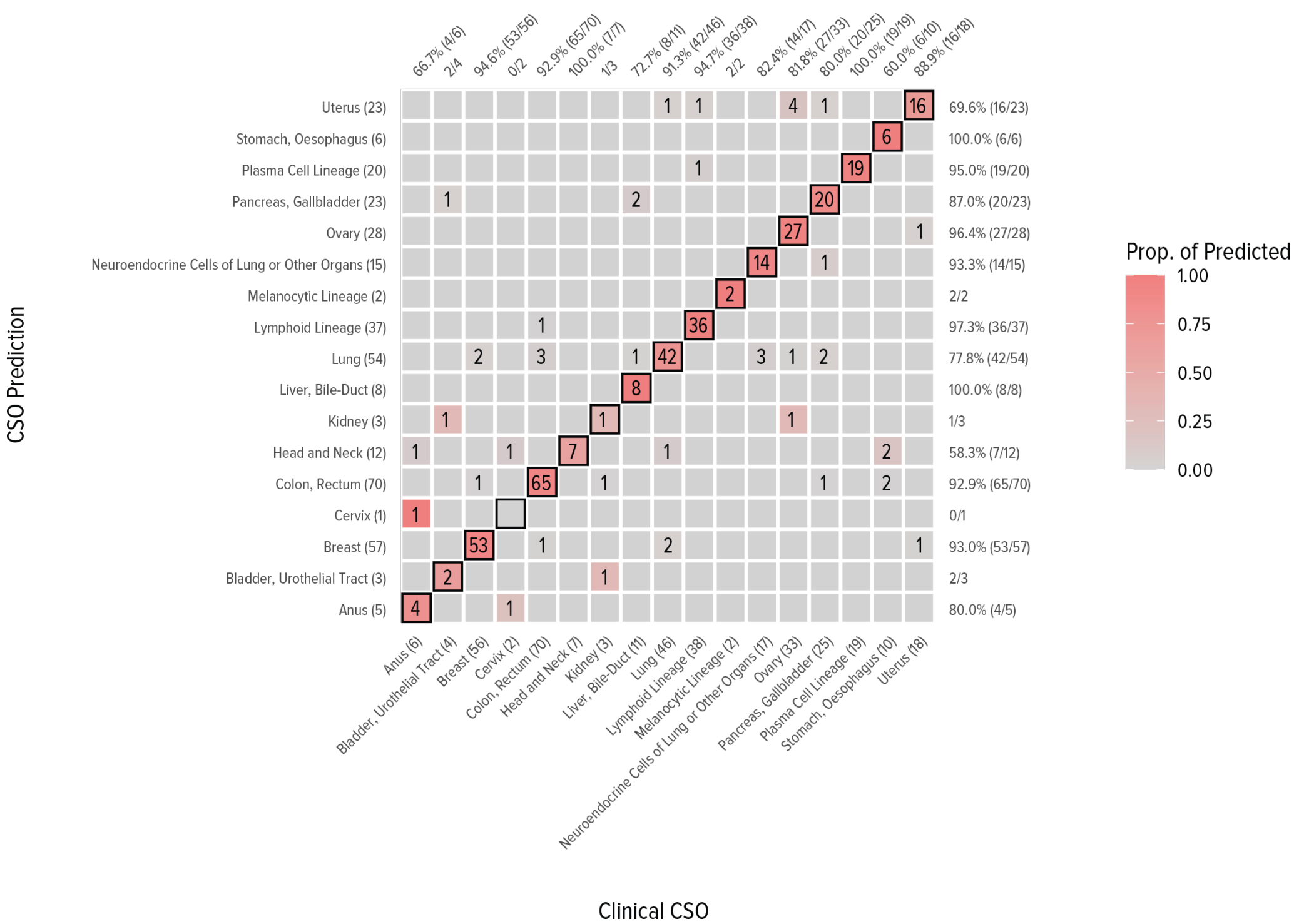

Male

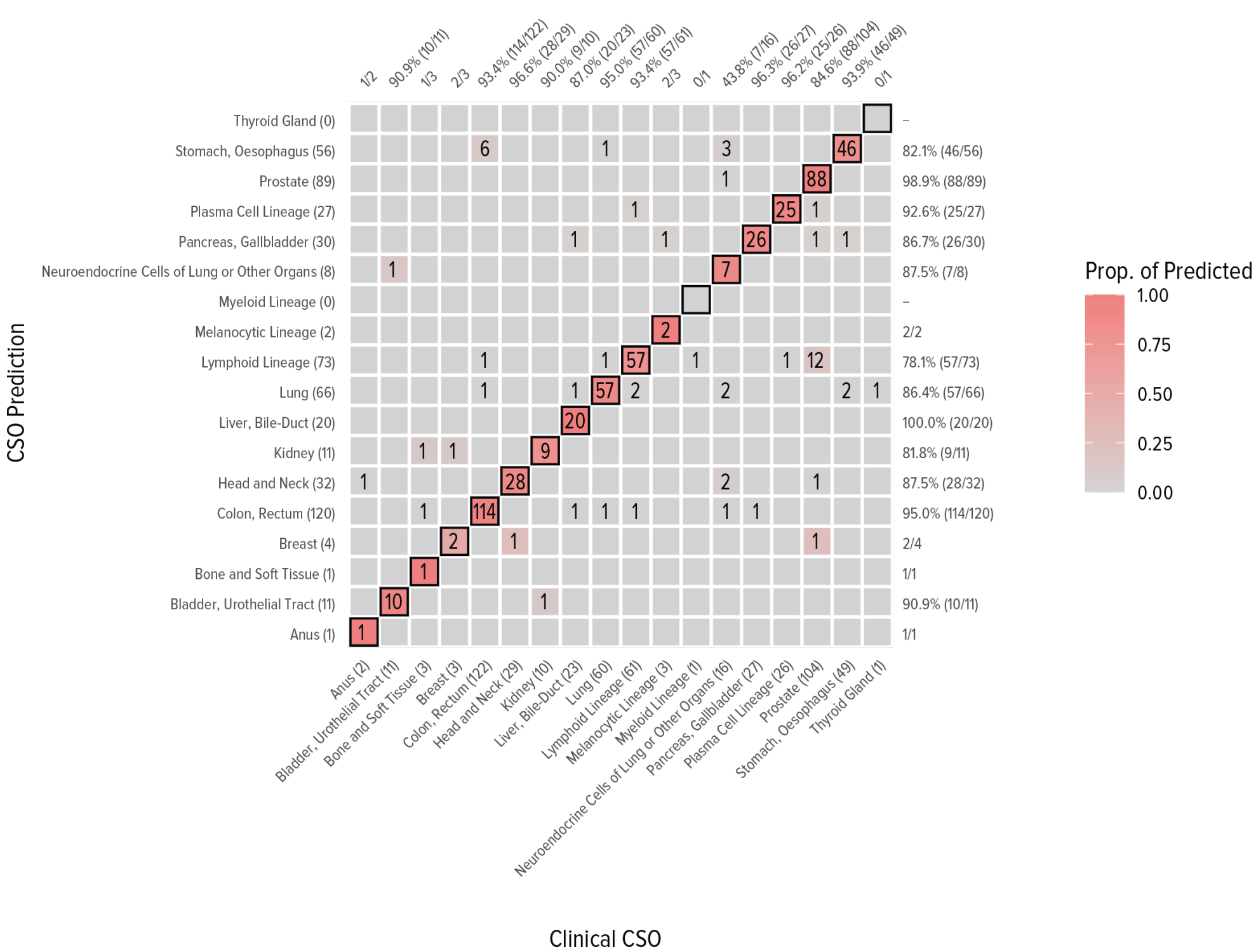

Each confusion matrix plots the number of participants for each combination of CSO prediction (y-axis) and clinical CSO (x-axis) for that round or in aggregate. Diagonal cells indicate correct CSO1 assignments (accuracy), and off-diagonal cells indicate misclassifications (errors). CSO1 prediction accuracy by clinical CSO (estimated by the proportion of the diagonal cell in each column) is indicated at the top of the matrix, and precision of each CSO prediction (estimated by the proportion of the diagonal cell in each row) along the right side. The colour is associated with the proportions of matched predicted and clinical CSOs. Metrics are not estimated for subgroups with less than 5 cases. Only CSOs with at least one prediction or diagnosed cancer are included in each matrix.

CSO, cancer signal origin; Prop, proportion.

Supplementary Figure S3. Aggregate PPV by Predicted CSO.

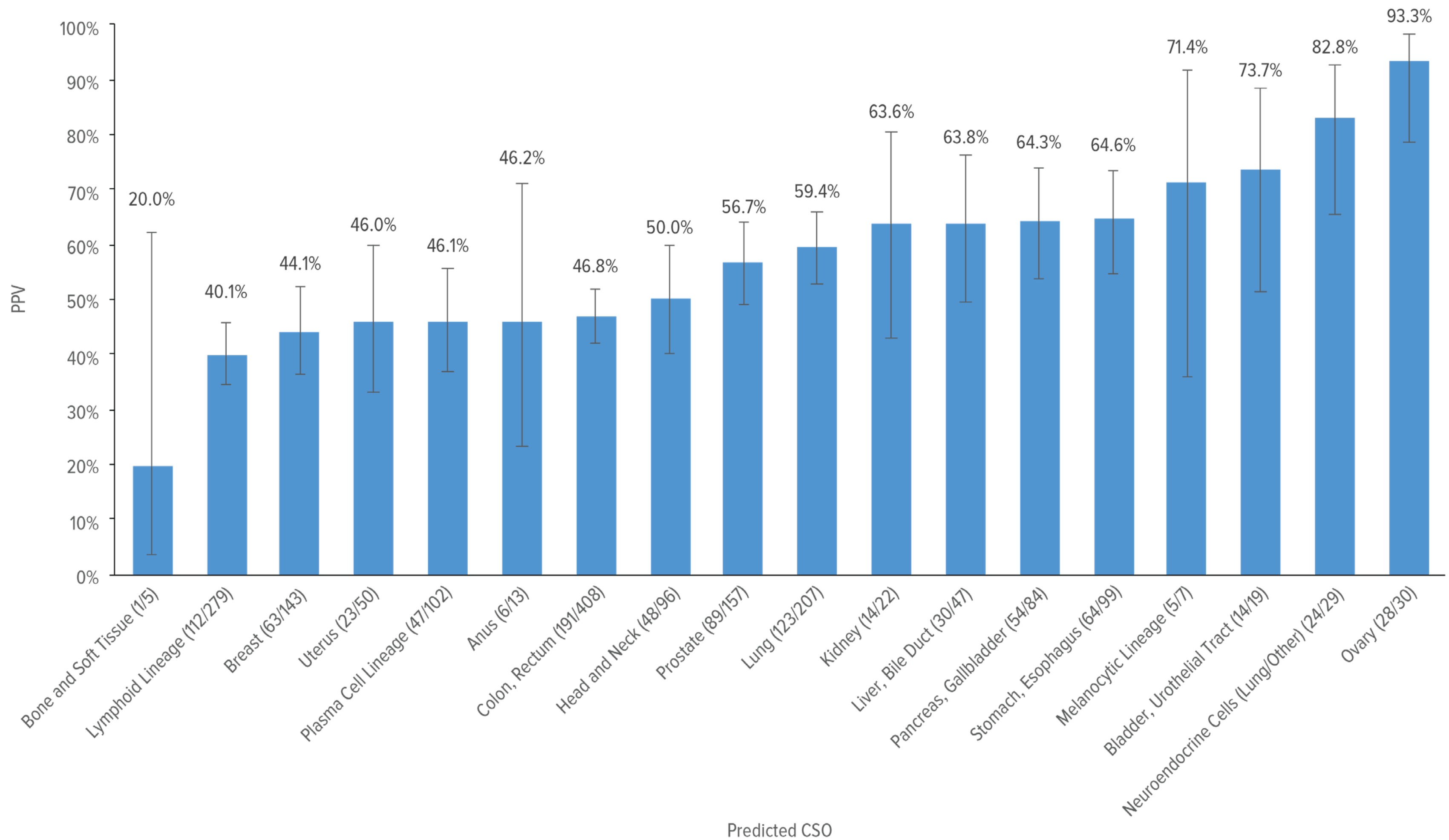

PPV data are presented as observed percentages, calculated as defined in **Supplementary Table S15**, for each predicted CSO; error bars denote two-sided 95% CIs. Predicted CSOs with less than 5 cases are not shown.

CSO, cancer signal origin; PPV, positive predictive value.

Supplementary Figure S4. Stage and Type of Cancer Diagnosed Among Participants With True-Positive and False-Negative MCED Test Results.

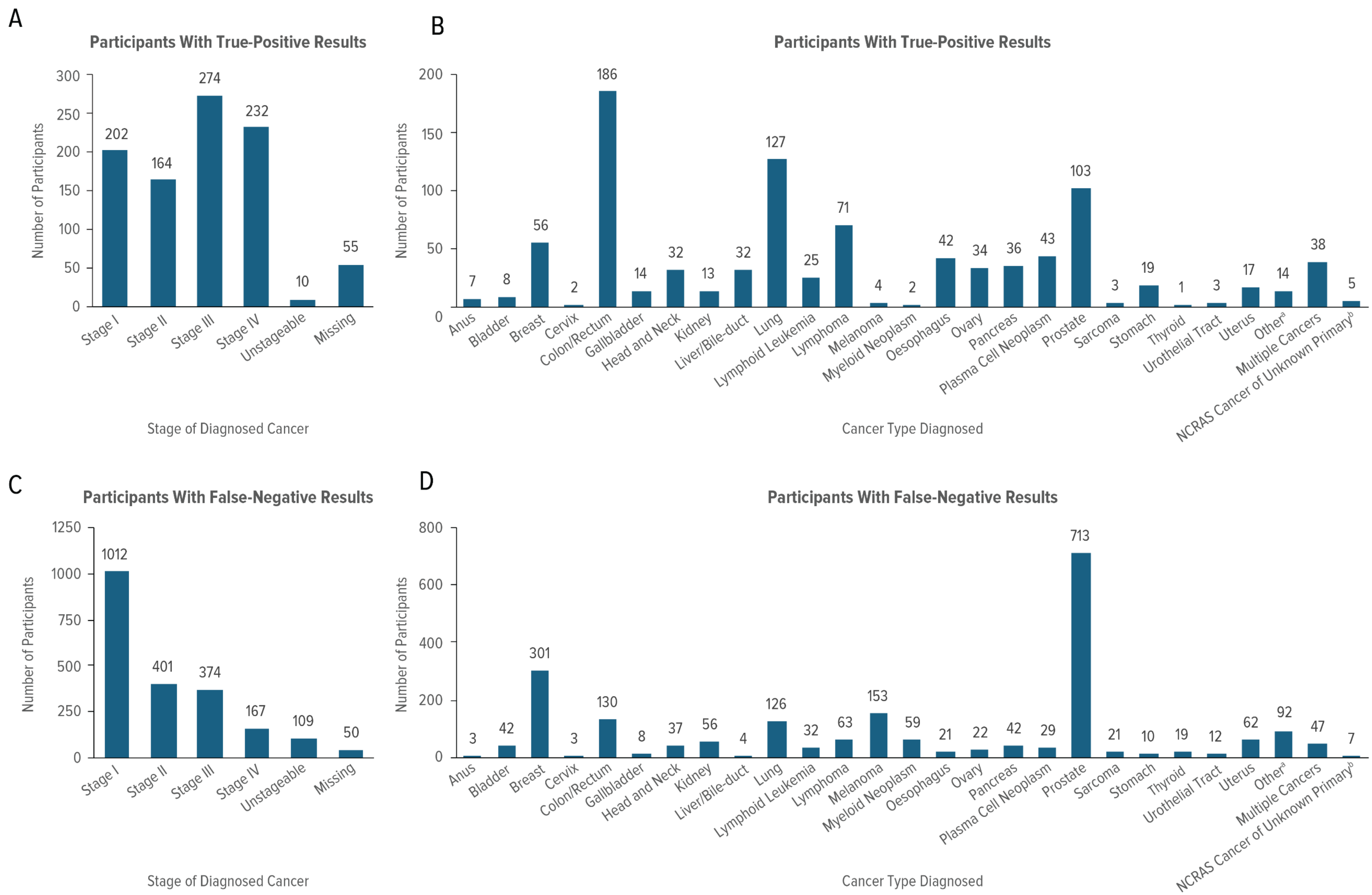

A. Number of participants across all three rounds of screening with a given stage of cancer at diagnosis who received a positive MCED test result for that round.

B. Number of participants across all three rounds of screening diagnosed with a given type of cancer who received a positive MCED test result for that round.

C. Number of participants across all three rounds of screening with a given stage of cancer at diagnosis who received a negative MCED test result for that round.

D. Number of participants across all three rounds of screening diagnosed with a given type of cancer who received a negative MCED test result for that round.

[a]Other cancer types include brain (n=32), mesothelioma (n=24), small intestine (n=16), vulva (n=10), penis (n=4), skin cancer (n=4), ampulla of Vater (n=3), germ cell (n=3), thymus (n=3), adrenal (n=2), vagina (n=2), carotid body (n=1), eyelid (n=1), trachea (n=1).

[b]NCRAS Cancer of Unknown Primary is defined as an invasive cancer with ICD-O-3 behaviour code 6 or 9; or an invasive cancer with behaviour code 3, topographical code C809 and unmapped cancer label.

ICD-O-3; International Classification of Diseases for Oncology, 3rd Edition; MCED, multi-cancer early detection; NCRAS, National Cancer Registration and Analysis Service.
