# Supplementary material for: Performance of a multi-cancer early detection test in the randomized controlled NHS-Galleri trial
Source: Nat Med. 2026 Sep 22;32(10):3770–80. doi: 10.1038/s41591-026-04652-8 (PMC13668664; doi:10.1038/s41591-026-04652-8)
Supplement: Supplementary file 2 [file 41591_2026_4652_MOESM2_ESM.pdf]

nature portfolio

Corresponding author(s): Peter Sasieni

Last updated by author(s): Aug 7, 2026

Reporting Summary

Nature Portfolio wishes to improve the reproducibility of the work that we publish. This form provides structure for consistency and transparency in reporting. For further information on Nature Portfolio policies, see our Editorial Policies and the Editorial Policy Checklist.

Statistics

For all statistical analyses, confirm that the following items are present in the figure legend, table legend, main text, or Methods section.

n/a	Confirmed	
☐	☒	The exact sample size (*n*) for each experimental group/condition, given as a discrete number and unit of measurement
☐	☒	A statement on whether measurements were taken from distinct samples or whether the same sample was measured repeatedly
☒	☐	The statistical test(s) used AND whether they are one- or two-sided<br>*Only common tests should be described solely by name; describe more complex techniques in the Methods section.*
☐	☒	A description of all covariates tested
☐	☒	A description of any assumptions or corrections, such as tests of normality and adjustment for multiple comparisons
☐	☒	A full description of the statistical parameters including central tendency (e.g. means) or other basic estimates (e.g. regression coefficient) AND variation (e.g. standard deviation) or associated estimates of uncertainty (e.g. confidence intervals)
☒	☐	For null hypothesis testing, the test statistic (e.g. *F*, *t*, *r*) with confidence intervals, effect sizes, degrees of freedom and *P* value noted<br>*Give P values as exact values whenever suitable.*
☒	☐	For Bayesian analysis, information on the choice of priors and Markov chain Monte Carlo settings
☒	☐	For hierarchical and complex designs, identification of the appropriate level for tests and full reporting of outcomes
☒	☐	Estimates of effect sizes (e.g. Cohen's *d*, Pearson's *r*), indicating how they were calculated

Our web collection on statistics for biologists contains articles on many of the points above.

Software and code

Policy information about availability of computer code

Data collection	Clinical data were captured in the GRAIL electronic data capture system, a validated and 21 CFR Part 11-compliant system, and supplemented with data from NCRAS datasets.
Data analysis	Analyses were conducted in Stata (Version 18; StataCorp LLC, College Station, TX) and independently validated in R (Version 4.3.2, R Core Team).

For manuscripts utilizing custom algorithms or software that are central to the research but not yet described in published literature, software must be made available to editors and reviewers. We strongly encourage code deposition in a community repository (e.g. GitHub). See the Nature Portfolio guidelines for submitting code & software for further information.

Data

Policy information about availability of data

All manuscripts must include a data availability statement. This statement should provide the following information, where applicable:

- Accession codes, unique identifiers, or web links for publicly available datasets
- A description of any restrictions on data availability
- For clinical datasets or third party data, please ensure that the statement adheres to our policy

Individual-level participant cancer outcome data are not publicly available and cannot be shared by the authors due to confidentiality requirements and restrictions under the applicable data-sharing agreements with National Cancer Registration and Analysis Service data providers. Aggregated, anonymised data supporting the

findings are provided in the manuscript and supplementary materials. The trial protocol and statistical analysis plan with amendments are provided as a supplement in Sasieni et al, 2026, N Eng J Med.

Research involving human participants, their data, or biological material

Policy information about studies with human participants or human data. See also policy information about sex, gender (identity/presentation), and sexual orientation and race, ethnicity and racism.

Reporting on sex and gender	Distribution of biological sex is provided in Table 1. Prespecified analysis of data stratified by sex is included in this manuscript (Fig 2, Extended Data Fig 2, and Supplementary Fig 2). Enrollment targets for sex distribution were specified in the protocol to ensure a representative study population. Sex was self-reported by the participants.
Reporting on race, ethnicity, or other socially relevant groupings	Distribution of race and ethnicity is included in the analysis (Table 1). Prespecified analyses of data stratified by race and ethnicity are included in the manuscript (Fig 2 and Extended Data Fig 5). Race/ethnicity categories were self-reported by participants at enrolment and grouped according to guidance from the Office of National Statistics as "White," "Black, African, Caribbean or Black British," "Asian or Asian British," "Mixed or Multiple ethnic groups," or "Other ethnic group.
Population characteristics	Distribution of demographics and baseline characteristics by screening round are shown in Table 1. Briefly, the analysis population comprised 70,325 participants in the first screening round, 64,498 in the second round, and 62,323 in the third round.<br><br>The median age of test performance analysable participants at enrolment was 66 years (IQR: 59-71) and 35,332 (50.2%) were female. Most participants were White (65,827 [93.6%]), reflecting England's population of the relevant age group. There was greater representation in the test performance analysable set from the more deprived quintile of the Index of Multiple Deprivation distribution: 22.6% (15,863) were in the most deprived quintile and 16.1% (11,348) were in the least deprived quintile 5. Most participants reported their smoking status as never smoked (38,574 [54.9%]) and 4680 (6.7%) reported they currently smoke. Based on body mass index calculated using self-reported height and weight at enrolment, 27,708 (39.4%) of participants were overweight (25 to <30 kg/m2) and 19,492 (27.7%) were obese (≥30 kg/m2). Baseline demographics of participants who returned for the second and third screening rounds were broadly similar to those of the first-round analysable set.
Recruitment	Participants from 8 Cancer Alliance regions in England were invited to enrol in NHS-Galleri. Invitations were sent primarily through centralized health service lists targeting older and more deprived groups, with some being invited through their general practice surgery (primary care provider) and a small number of participants self-referring following targeted engagement.<br><br>Participants were enrolled between 31 August 2021 and 16 July 2022. The event cutoff date for data included in this manuscript was 12 July 2025 with a lock date of 28 January 2026 for analysis.
Ethics oversight	The trial was conducted in accordance with the Declaration of Helsinki, Council for International Organizations of Medical Sciences International Ethical Guidelines, applicable ICH Good Clinical Practice guidance, and relevant laws and regulations. The protocol was approved by Wales Research Ethics Committee 1 (Ref: 21/WA/0141). Health Research Authority approval was granted with support from the Confidentiality Advisory Group (Ref: 21/CAG/0056), under Regulation 5 of the Health Service Regulations 2002 ("Section 251" support). An Independent Data Monitoring Committee oversaw trial progress, reviewed clinical and safety data, and provided recommendations to the sponsor regarding trial conduct. All participants provided written informed consent.

Note that full information on the approval of the study protocol must also be provided in the manuscript.

Field-specific reporting

Please select the one below that is the best fit for your research. If you are not sure, read the appropriate sections before making your selection.

☒ Life sciences ☐ Behavioural & social sciences ☐ Ecological, evolutionary & environmental sciences

For a reference copy of the document with all sections, see nature.com/documents/nr-reporting-summary-flat.pdf

Life sciences study design

All studies must disclose on these points even when the disclosure is negative.

Sample size	The sample size of the NHS-Galleri trial was based on microsimulations that indicated approximately 140,000 participants were necessary for ≥90% power for the between-arm primary endpoint (stage III/IV reduction) after 3 rounds of screening, with 12 months of follow-up after the last participant's third visit, including various natural history assumptions.
Data exclusions	Data from individuals for whom valid informed consent was not available were excluded from all research analyses and were not included in the consented analysis set. All participants in the consented analysis set were retained in the participant-disposition diagram and safety analyses. Test-performance analyses were conducted in the prespecified test performance analysable set, defined as intervention-arm participants who were clinically eligible for the relevant screening round, had an evaluable MCED test result, and were successfully linked to the National Cancer Registration and Analysis Service (NCRAS) to ascertain cancer status.
Replication	This study was not designed as a replication study.

Randomization

Following informed consent and peripheral blood sample collection, participants were randomised 1:1, with allocation concealed at the point of randomisation, to either the intervention arm (blood tested with the MCED test, with positive results returned to the participant) or to the control arm (blood stored).

Blinding

Study staff and participants were initially blinded. Unblinded trial nurses communicated positive MCED test results to intervention-arm participants who had a positive MCED test result. Thus these participants were unblinded for the rest of the trial. Unblinded independent IDMC statisticians and the medical monitor reviewed safety data during the study.

Reporting for specific materials, systems and methods

We require information from authors about some types of materials, experimental systems and methods used in many studies. Here, indicate whether each material, system or method listed is relevant to your study. If you are not sure if a list item applies to your research, read the appropriate section before selecting a response.

Materials & experimental systems

n/a	Involved in the study
☒	☐ Antibodies
☒	☐ Eukaryotic cell lines
☒	☐ Palaeontology and archaeology
☒	☐ Animals and other organisms
☐	☒ Clinical data
☒	☐ Dual use research of concern
☒	☐ Plants

Methods

n/a	Involved in the study
☒	☐ ChIP-seq
☒	☐ Flow cytometry
☒	☐ MRI-based neuroimaging

Clinical data

Policy information about clinical studies

All manuscripts should comply with the ICMJE guidelines for publication of clinical research and a completed CONSORT checklist must be included with all submissions.

Clinical trial registration

NCT05611632 and ISRCTN91431511

Study protocol

The complete study protocol and statistical analysis plan with amendments are published in Sasieni et al, 2026, N Eng J Med.

Data collection

Participants from 8 Cancer Alliance regions in England were invited to enrol in NHS-Galleri. Invitations were sent primarily through centralized health service lists targeting older and more deprived groups, with some being invited through their general practice surgery (primary care provider) and a small number of participants self-referring following targeted engagement.

Participants were enrolled between 31 August 2021 and 16 July 2022. The event cutoff date for data included in this manuscript was 12 July 2025 with a lock date of 28 January 2026 for analysis.

Outcomes

The trial outcome data, including cancer diagnoses, staging, and mortality, were obtained from NHS sources by NCRAS. Cancer diagnosis data were passively collected for all participants throughout the trial. The clinical outcome of cancer was defined as a confirmed diagnosis of a haematologic malignancy (with behaviour code 3 based on ICD-O-3) or an invasive solid tumour, excluding basal cell carcinoma and squamous cell carcinoma of the skin.

Primary, secondary and exploratory endpoints were predefined in the study protocol and statistical analysis plan (published in Sasieni et al, 2026, N Eng J Med). A description of endpoints and how each were assessed is in the Methods.

Plants

Seed stocks

Report on the source of all seed stocks or other plant material used. If applicable, state the seed stock centre and catalogue number. If plant specimens were collected from the field, describe the collection location, date and sampling procedures.

Novel plant genotypes

Describe the methods by which all novel plant genotypes were produced. This includes those generated by transgenic approaches, gene editing, chemical/radiation-based mutagenesis and hybridization. For transgenic lines, describe the transformation method, the number of independent lines analyzed and the generation upon which experiments were performed. For gene-edited lines, describe the editor used, the endogenous sequence targeted for editing, the targeting guide RNA sequence (if applicable) and how the editor was applied.

Authentication

Describe any authentication procedures for each seed stock used or novel genotype generated. Describe any experiments used to assess the effect of a mutation and, where applicable, how potential secondary effects (e.g. second site T-DNA insertions, mosiacism, off-target gene editing) were examined.
